# Exercise Training in Obese Rats Does Not Induce Browning at Thermoneutrality and Induces a Muscle-Like Signature in Brown Adipose Tissue

**DOI:** 10.3389/fendo.2020.00097

**Published:** 2020-03-20

**Authors:** Peter Aldiss, Jo E. Lewis, Irene Lupini, Ian Bloor, Ramyar Chavoshinejad, David J. Boocock, Amanda K. Miles, Francis J. P. Ebling, Helen Budge, Michael E. Symonds

**Affiliations:** ^1^The Early Life Research Unit, Division of Child Health, Obstetrics and Gynaecology, School of Medicine, University of Nottingham, Nottingham, United Kingdom; ^2^School of Life Sciences, Queen's Medical Centre, University of Nottingham, Nottingham, United Kingdom; ^3^School of Biosciences and Veterinary Medicine, University of Camerino, Camerino, Italy; ^4^John van Geest Cancer Research Centre, Nottingham Trent University, Nottingham, United Kingdom; ^5^Nottingham Digestive Disease Centre and Biomedical Research Unit, School of Medicine, University of Nottingham, Nottingham, United Kingdom

**Keywords:** adipose tissue, exercise training, obesity, housing temperature, browning

## Abstract

**Aim:** Exercise training elicits diverse effects on brown (BAT) and white adipose tissue (WAT) physiology in rodents housed below their thermoneutral zone (i.e., 28–32°C). In these conditions, BAT is chronically hyperactive and, unlike human residence, closer to thermoneutrality. Therefore, we set out to determine the effects of exercise training in obese animals at 28°C (i.e., thermoneutrality) on BAT and WAT in its basal (i.e., inactive) state.

**Methods:** Sprague-Dawley rats (*n* = 12) were housed at thermoneutrality from 3 weeks of age and fed a high-fat diet. At 12 weeks of age half these animals were randomized to 4-weeks of swim-training (1 h/day, 5 days per week). Following a metabolic assessment interscapular and perivascular BAT and inguinal (I)WAT were taken for analysis of thermogenic genes and the proteome.

**Results:** Exercise attenuated weight gain but did not affect total fat mass or thermogenic gene expression. Proteomics revealed an impact of exercise training on 2-oxoglutarate metabolic process, mitochondrial respiratory chain complex IV, carbon metabolism, and oxidative phosphorylation. This was accompanied by an upregulation of multiple proteins involved in skeletal muscle physiology in BAT and an upregulation of muscle specific markers (i.e., Myod1, CkM, Mb, and MyoG). UCP1 mRNA was undetectable in IWAT with proteomics highlighting changes to DNA binding, the positive regulation of apoptosis, HIF-1 signaling and cytokine-cytokine receptor interaction.

**Conclusion:** Exercise training reduced weight gain in obese animals at thermoneutrality and is accompanied by an oxidative signature in BAT which is accompanied by a muscle-like signature rather than induction of thermogenic genes. This may represent a new, UCP1-independent pathway through which BAT physiology is regulated by exercise training.

## Introduction

During obesity, the accumulation of excess lipid and subsequent hypertrophy of adipocytes leads to adipose tissue (AT) dysfunction (1). These deleterious alterations in obese AT include macrophage infiltration and apoptosis, an increase in, and secretion of, inflammatory cytokines, hypoxia, and insulin resistance, all of which contribute to systemic cardiometabolic risk (1–3).

Given that sustainable weight loss is hard to achieve, improving the AT phenotype is one potential avenue to preventing the onset of diseases associated with obesity. Exercise training elicits diverse effects on both general metabolic parameters (i.e., improved insulin sensitivity) and on the AT phenotype (4–7). Following exercise training, there is a switch from a pro-inflammatory M1 to a M2 macrophage phenotype where inflammation is inhibited (8). Increased vascular endothelial growth factor A and reduced AT lactate following exercise suggest an induction of AT angiogenesis and reduction in AT hypoxia whilst improving AT adipokine secretion, oxidative stress, mitochondrial biogenesis and insulin sensitivity (4–6, 9).

More recently, there has been a focus on the role of exercise training to regulate the thermogenic programme in brown (BAT) and white AT (WAT) (10). BAT has a high oxidative capacity similar to skeletal muscle, utilizing glucose and free fatty acids (FFA) as substrates for cold and diet-induced thermogenesis following the activation of uncoupling protein (UCP)1 (11). Yet, how exercise training regulates BAT physiology is unclear. Exercise has been shown to induce a “whitening” of BAT and reduce insulin-stimulated glucose uptake, whilst promoting the appearance of “beige/brite” adipocytes in classical WAT (12–14). This adaptation has been attributed to a range of mechanisms including the downstream actions of various myokines (e.g., meteorin-like) (15), hepatokines (e.g., fibroblast growth factor 21) (16), and metabolites (e.g., β-aminoisobutyric acid) (17). Importantly, this occurs regardless of exercise modality (i.e., treadmill, swim training, and voluntary wheel running) (18).

An important caveat, however, is that rodents subjected to exercise are typically housed at c.20°C, a temperature well below their thermoneutral zone (19). This impacts on a number of physiological processes including adaptive thermogenesis, cardiovascular function, and immune cell metabolism (19, 20). In particular, it is an important consideration when studying BAT, which is chronically active at 20°C with UCP1+ adipocytes readily seen in WAT (21). Importantly, BAT from obese mice housed chronically at thermoneutrality, closer resembles human BAT and this model of “physiologically humanized BAT” represents the best choice for modeling BAT physiology (22). Therefore, we used chronic thermoneutrality (i.e., 28°C from weaning) to closer mimic human physiology and study AT in the basal state (i.e., when UCP1 is inactive). We analyzed the effects of exercise training on animals kept at thermoneutrality on both interscapular (BAT) and perivascular (PVAT) BAT, having previously shown these depots to exhibit a divergent response to brief nutrient excess (23), as well as IWAT hypothesizing that exercise induced “browning” would be absent under these conditions. Finally, we sought to identify how the AT proteome responds to exercise training to better understand the molecular adaptations of BAT to training at thermoneutrality.

## Methods

### Animals, Exercise Protocol, and Metabolic Assessment

All studies were approved by the University of Nottingham Animal Welfare and Ethical Review Board, were carried out in accordance with the UK Animals (Scientific Procedures) Act of 1986. Twelve male Sprague-Dawley rats aged 3 weeks were obtained from Charles River (Kent, UK) and housed (3 per cage) immediately at thermoneutrality (c.28°C) under a 12:12-h reverse light-dark cycle (lights off at 08:00 a.m./ZT12, on at 20:00 p.m./ZT0) so as to closer mimic human physiology (21), minimize animal stress and maximize data quality and translatability (24). Animals were fed a high-fat diet (45%, 824018 SDS, Kent, UK) *ad-libitum* with body weight monitored weekly throughout. Half of the animals were then randomized (http://www.graphpad.com/quickcalcs/randomize1.cfm) to 4 weeks of exercise training (Ex) at 12 weeks of age. Then, the Ex group were acclimatized to water (c.35°C) for a 3-day period (10–20 min per day) at the beginning of the dark phase (i.e., ZT13). After acclimatization, the Ex group underwent the 4-week swim training programme (1 h/day for 5 days/week at ZT13). As described by the American Physiological Society, “Continuous swimming involves continuous movement of the rat's forelimbs and hindlimbs while maintaining its snout above the waterline” (25). We confirmed this behavior, and the ability of each animal to swim, prior to commencing the training programme. Following each session, animals were towel dried and placed back in their home cage underneath a heat lamp.

Animals were individually placed in an open-circuit calorimeter (CLAMS: Columbus Instruments, Linton Instrumentation, UK) for 48 h following training and prior to tissue collection. Assessment of whole body metabolism was performed as previously described (23), after which all animals were weighed and fasted overnight prior to euthanasia at ZT12-ZT15 by rising CO_2_ gradient. BAT, IWAT, PVAT from the thoracic aorta and portion of the central liver lobe were then rapidly dissected, weighed, snap-frozen in liquid nitrogen and stored at −80°C for subsequent analysis. All fat depots were excised and weighed to calculate total fat mass.

### Histology

Brown and inguinal adipose tissue samples were fixed in formalin for 96 h and embedded in paraffin wax using an Excelsior ES tissue processor (Thermo-Fisher). Sections were cut from each sample at 8 μm, mounted on Superfrost Plus slides (Fisher Scientific) and stained using haematoxylin and eosin (Sigma-Aldrich). Three to five randomly selected sections per sample were imaged and calibrated using an Olympus BX40 microscope with a charge-coupled device high-speed color camera (Micropublisher 3.3RTV; QImaging) at 10x magnification using Volocity v6.1 software (Perkin Elmer). BAT and WAT cell area was determined using Adiposoft (26), an automated image analyzing java plugin for Image J (Fiji).

### Gene Expression Analysis

Total RNA was extracted from each fat depot using the RNeasy Plus Micro extraction kit (Qiagen, West Sussex, UK) following an adapted version of the single step acidified phenol-chloroform method. RT-qPCR was carried out as previously described (23) using rat-specific oligonucleotide primers (Sigma-Aldrich) or FAM-MGB Taqman probes (see Supplementary Table 1 for primer list). Gene expression was determined using the GeNorm algorithm against two selected reference genes; *RPL19:RPL13a in BAT* and IWAT (stability value M = 0.26 in BAT and 0.224 in IWAT) and RPL19:HPRT1 in PVAT (stability value M = 0.209).

### Serum and Liver Analysis

Blood was taken by cardiac puncture and allowed to clot for ~30 min at room temperature. Samples were then centrifuged at 2000G for 10 min and the serum removed and stored at −80°C until use. Serum was thawed gently on ice. Concentrations of glucose (GAGO-20, Sigma-Aldrich, Gillingham, UK), triglycerides (LabAssay™ Triglyceride, Wako, Neuss, Germany), non-esterified fatty acids (NEFA-HR(2), Wako, Neuss, Germany), insulin (80-INSRT-E01, Alpco, Salem, NH, USA), and leptin (EZRL-83K, Merck, Darmstadt, Germany) were measured following manufacturers' instructions. HOMA-IR was determined by calculating fasting insulin (μ U/mL) x fasting glucose (mg/dl)/405. Hepatic triglycerides were quantified using the Triglyceride Quantification Assay Kit (Colorimetric/Fluorometric) (ab65336).

### Adipose Tissue Proteomics

Protein extraction, clean up and trypsinisation was carried out as previously described (23). Briefly, 50–100 mg of frozen BAT and IWAT was homogenized in 500 μL CellLytic MT cell lysis buffer (Sigma-Aldrich, C3228) prior to removal of lipid and other non-protein components using the ReadyPrep 2D clean up Kit (Biorad, 1632130). Samples (*n* = 4/group) were then subjected to reduction, alkylation and overnight trypsinisation, following which they were dried down at 60°C for 4 h and stored at −80°C before resuspension in LCMS grade 5% acetonitrile in 0.1% formic acid for subsequent analysis. Analysis by mass spectrometry was performed on a SCIEX TripleTOF 6600 instrument as previously described (27). Briefly, samples were analyzed in both SWATH (Data Independent Acquisition) and IDA (Information Dependent Acquisition) modes for quantitation and spectral library generation respectively. IDA data was searched using ProteinPilot 5.0.2 to generate a spectral library and SWATH data was analyzed using Sciex OneOmics software (28) extracted against the locally generated library as described previously (23). The mass spectrometry proteomics data have been deposited to the ProteomeXchange Consortium via the PRIDE partner repository with the dataset identifier PXD017306 (29).

### Statistical Analyses

Statistical analyses were performed in GraphPad Prism version 8.0 (GraphPad Software, San Diego, CA). Data are expressed as Mean ± SEM with details of specific statistical tests in figure legends. Functional analysis of the proteome (fold change ± 0.5 and OneOmics confidence score cut-off of 0.75) was performed using the Advaita Bioinformatic iPathwayGuide software (www.advaitabio.com/ipathwayguide.html). Significantly-impacted biological processes, molecular interactions, and pathways were analyzed in the context of pathways obtained from the Kyoto Encyclopedia of Genes and Genomes database (Release 84.0+/10-26, Oct 17) (30) and the Gene Ontology Consortium database (2017-Nov) (31). The Elim pruning method, which removes genes mapped to a significant GO term from more general (higher level) GO terms, was used to overcome the limitation of errors introduced by considering genes multiple times (32). Analysis of protein-protein interactions (PPI) and GO term enrichment of these PPI networks was performed using NetworkAnalyst (www.networkanalyst.ca). Differentially regulated proteins were imported into NetworkAnalyst (https://www.networkanalyst.ca/NetworkAnalyst/home.xhtml) and protein-protein interactions were determined using the STRING interactome database with a confidence score cut-off of 900 and requirement of experimental evidence.

## Results

### Exercise Training Increases BAT Mass and Regulates Thermogenic Genes in Perivascular BAT and Inguinal WAT

**Figure 1:**
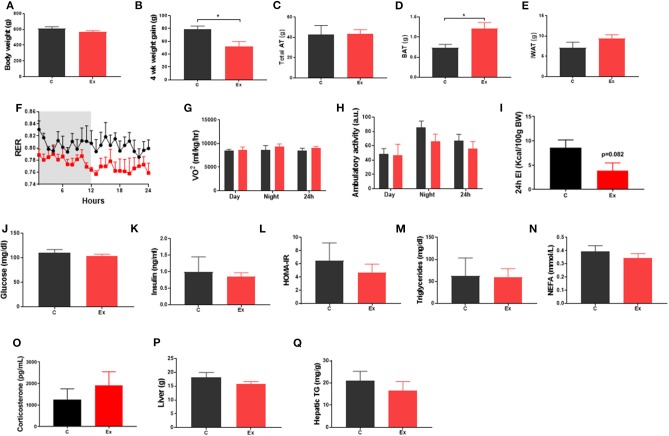
Exercise training (Ex) attenuated weight gain and increased brown adipose tissue (BAT) mass but had no effect on insulin, glucose, triglycerides, or non-esterified fatty acids compared to no training **(C)**. **(A)** Final body weight, **(B)** 4 week intervention weight gain, **(C)** total fat mass, **(D)** BAT mass, **(E)** inguinal white adipose tissue (IWAT) mass, **(F)** respiratory exchange ratio (RER), **(G)** oxygen consumption (VO_2_), **(H)** ambulatory activity. **(I)** 24h energy intake **(J)** serum glucose, **(K)** insulin, **(L)** HOMA-IR, **(M)** triglycerides, **(N)** NEFA and **(O)** Corticosterone, **(P)** liver weight and **(Q)** hepatic triglycerides. Data **(F–I)** obtained from CLAMs metabolic cages. All data expressed as mean ± SEM, *n* = 4–5 per group. For comparison, data was analyzed by either Students *t*-test **(A–E, I–P)** or two-way ANOVA **(F–H)** and Sidak *post-hoc* tests. Significance denoted as ^*^p < 0.05.

**Figure 2:**
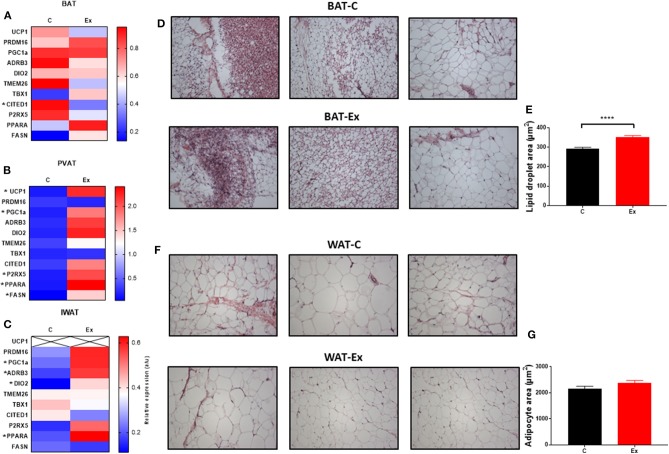
Exercise training (Ex) regulates thermogenic genes in a depot-specific manner. **(A–C)** thermogenic mRNA in BAT, PVAT, and IWAT, **(D,E)** histological analysis of BAT and lipid droplet size and **(F,G)** histological analysis of WAT and adipocyte size. Data expressed as mean ± SEM, *n* = 4–5 per group. Adipocyte/lipid droplet area quantified using Adiposoft (BAT-C, *n* = 8,949 and Ex, *n* = 8,234; WAT-C, *n* = 750 and Ex – *n* = 555). For comparison, data was analyzed by Students *t*-test. Significance denoted as ^*^p < 0.05 and ^****^p < 0.0001.

Swim training in diet-induced obesity did not affect body weight, subcutaneous fat mass or total fat mass (i.e., the weight of all dissected fat depots) but significantly attenuated weight gain during the 4-week intervention period (HFD: 78.6 ± 5.3 vs. Ex: 52 ± 7.7g, *p* = 0.03; Figures 1A–C, E) without effect on serum insulin or metabolites (Figures 1J–O), hepatic weight or hepatic triglycerides (Figures 1P,Q) (Supplementary Data). We attribute this attenuation in weight-gain to a trend in both EI (*p* = 0.08, Figure 1I) and RER (*p* = 0.09, Figure 1F) to decline given there is no effect on VO_2_ or ambulatory activity (Figures 1G,H). Despite attenuated weight gain, BAT mass increased along with a significant increase in lipid droplet size (Figures 1D and 2D,E) although no difference in key thermogenic (e.g., UCP1) or lipogenic (FASN) mRNA levels (Figure 2A) was detected. Interestingly, UCP1 was upregulated in PVAT along with PGC1α, a marker of mitochondrial biogenesis and P2RX5, a purinergic receptor and brown/beige adipocyte cell surface marker (Figure 2B). Similarly, mRNA's governing fatty-acid oxidation (PPARA) and lipogenesis (FASN) were upregulated in PVAT. With regards to “browning,” UCP1 mRNA was undetectable in IWAT and was not induced with exercise training despite an upregulation of PGC1a, ADRB3, DIO2 and PPARA (Figure 2C). Morphologically, BAT was characterized by a heterogeneous mixture of classic, BAT-like tissue, small multilocular lipid droplets and small to large adipocytes with exercise training driving a significant increase in lipid droplet size (Figures 2D,E) whereas there was no discernable difference in IWAT (Figures 2F,G) which was characterized by large adipocytes and no sign of multilocular, beige adipocytes as is evident in WAT at lower ambient temperatures.

### Identification of Differentially Regulated Proteins in BAT and IWAT in Response to Swim-Training

We then sought to determine the exercise-induced effects on the proteome of these BAT and IWAT depots. We identified 353 differentially regulated proteins in BAT (Table 1: Top 20 proteins; Supplementary Table 1: Full list). The most significantly altered proteins were involved in mitochondrial ATP synthesis (ATP5E), nucleopore (NUP35), ADP ribosylation (ARF1 and SCOC), and progesterone binding (PGMRC2). Among the proteins most upregulated in BAT were those involved in assembly of the skeletal muscle cytoskeleton (PDLIM3 and MYH4), muscle contraction (TNNI2), and muscle-specific phosphoglycerate mutase metabolism (PGAM2). Proteins involved in calcium sensing in the lumen of the sarcoplasmic reticulum (CASQ1) and beta adrenergic signaling (CAPN1 and PSMB7) were found to be the most downregulated in BAT. Conversely, only 189 proteins were differentially regulated in IWAT after exercise. The most significantly altered proteins (Table 2: Top 20 proteins; Supplementary Table 2: Full list) were involved in the trafficking of GLUT4 (TUSC5), the mitochondrial electron transport chain (NDUFS6), beta adrenergic signaling (PSMB7), TLR4 signaling (LRRFIP2), and apoptosis (ATG7 and BIN1). Proteins with the greatest fold change were those involved in cell adhesion (CD44 and VCAN), FFA and lipoprotein metabolism (FABP3 and APOC1), TGF-beta signaling (TSC22D1), purine and mitochondrial metabolism (LHPP and COX6A1), and the acetylation of nucleosomes and DNA binding (HET and HMGB1).

**Table 1:** Top 20 differentially regulated proteins in BAT.

**Symbol**	**Entrez**	**Logfc**	**adjpv**
Picalm	89816	−1.77605	3.83E-06
Scoc	364981	−1.84724	7.37E-05
Trim72	365377	0.773012	0.000139
Arf1	64310	−2.72857	0.00015
Rbbp9	29459	−1.79555	0.000168
Rps26	27139	−0.57111	0.000217
Pgrmc2	361940	−1.29142	0.00027
Atp5e	245958	1.29928	0.000345
Ruvbl1	65137	−2.89092	0.000414
Serpinb1a	291091	−0.6765	0.000424
Rnmt	291534	2.861109	0.000447
Nup35	295692	1.47585	0.000704
Ggcx	81716	0.762945	0.00088
Rpl21	79449	−3.79095	0.000976
Farsa	288917	−2.23967	0.001057
Pon1	84024	1.269484	0.001358
Vars	25009	0.905263	0.001382
Slc3a2	50567	1.68137	0.001386
Acox1	50681	−1.05358	0.001481
Rab7a	29448	−0.56428	0.001598

*Entrez gene ID (Entrez), log fold change (Logfc) where minus symbol equals downregulation, adjusted P value (adjPval)*.

**Table 2:** Top 20 differentially regulated proteins in IWAT.

**Symbol**	**Entrez**	**Logfc**	**adjpv**
Lrrfip2	301035	−1.62311	7.61E-06
RGD1311739	311428	0.886759	0.000023
Psmb7	85492	2.732771	0.000195
Ndufs6	29478	2.213416	0.000496
Ezr	54319	−0.7888	0.0005
Orm1	24614	1.11547	0.001359
Bin1	117028	−3.21998	0.001856
Stk3	65189	−1.51626	0.001979
Gna11	81662	0.563094	0.002852
Sult1a1	83783	0.868093	0.003031
Sod3	25352	0.639222	0.003225
Atg7	312647	−2.31741	0.004631
Rpl38	689284	−1.10403	0.006447
Pip4k2a	116723	−1.89092	0.006578
Tusc5	360576	0.802222	0.007389
Usp7	360471	−0.81645	0.00841
Cd14	60350	0.942401	0.008533
Pdcd10	494345	−0.80063	0.009051
Timm8a1	84383	−1.10352	0.010498
EGF	25313	3.510509	0.01074

*Entrez gene ID (Entrez), log fold change (Logfc) where minus symbol equals downregulation, adjusted P value (adjPval)*.

### Exercise Training Enriches Mitochondrial and Skeletal Muscle Related GO Terms and Pathways in BAT

**Figure 3:**
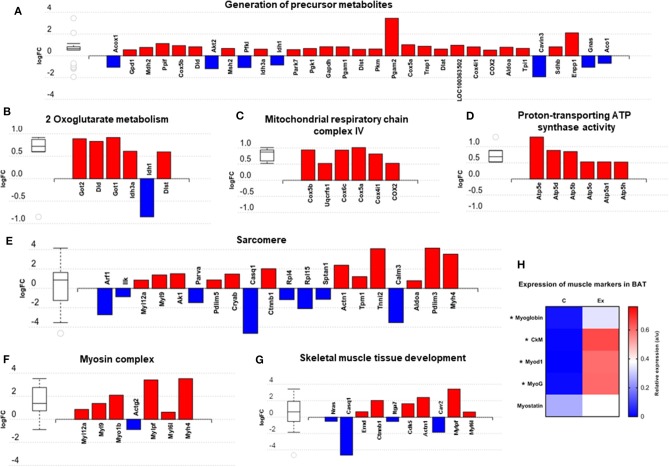
Overview of enriched metabolic and skeletal muscle related gene ontology (GO) terms in brown adipose tissue **(A–G)** and expression of myogenic markers **(H)**. Figures created with AdvaitaBio IPathway Guide. *N* = 4–5/group, for analysis see methods section.

**Figure 4:**
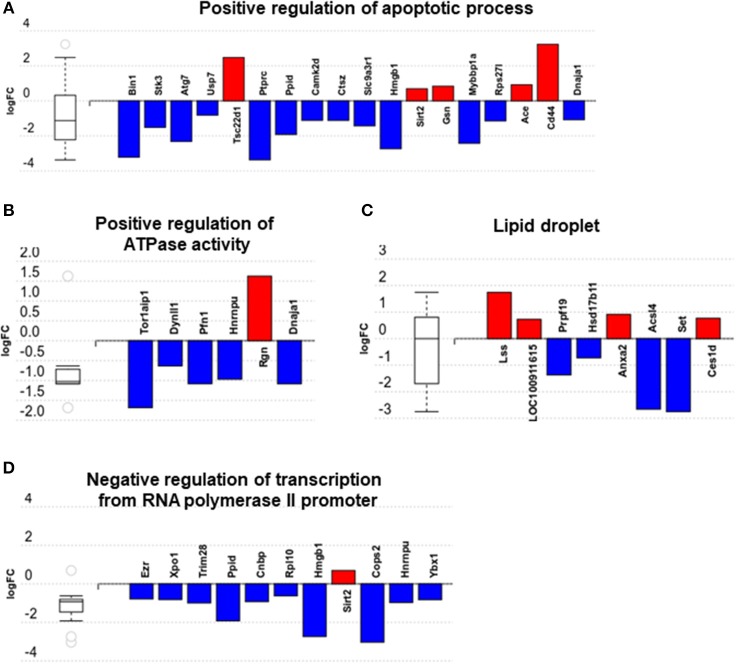
Overview of enriched gene ontology (GO) terms in inguinal white adipose tissue **(A–D)**. Figures created with AdvaitaBio IPathwayGuide. *N* = 4/group, for analysis see methods section.

We then carried out functional analysis of the BAT and IWAT proteome. The differentially regulated proteins in BAT enriched GO terms (Table 3; Supplementary Table 3: Full list) including *2*-*oxoglutarate metabolic process, generation of precursor metabolites, cytochrome-c oxidase activity, mitochondrial respiratory chain complex IV and proton transporting ATP synthase activity* (Figures 3A–D). There was also an enrichment of GO terms related to skeletal muscle physiology including *sarcomere, myosin complex, and skeletal muscle tissue development* (Figures 3E–G). This enrichment was associated with a significant upregulation of skeletal muscle markers (Figure 3H) including Myoglobin, Myogenic differentiation 1 (MYOD1) and Myogenin (MYOG). In IWAT, (Table 4; Supplementary Table 4 for full list) the differentially regulated proteins enriched GO terms including *positive regulation of apoptotic process, positive regulation of ATPase activity* and *lipid droplet* (Figures 4A–C). In addition, a number of GO terms associated with RNA processing were enriched including *spliceosomal complex and negative regulation of transcription from RNA polymerase II promoter* (Figure 4D).

**Table 3:** GO terms enriched in BAT.

**Go Id**	**goName**	**countDE**	**countAll**	**pv_elim**
**BIOLOGICAL PROCESS**				
GO:0003151	Outflow tract morphogenesis	4	4	0.0053
GO:0006103	2-oxoglutarate metabolic process	6	8	0.0064
GO:0051304	Chromosome separation	5	6	0.0067
GO:0007093	Mitotic cell cycle checkpoint	6	9	0.0148
GO:0014075	Response to amine	6	9	0.0148
GO:0014044	Schwann cell development	5	7	0.0182
GO:0018198	Peptidyl-cysteine modification	5	7	0.0182
GO:2000273	Positive regulation of receptor activity	5	7	0.0182
GO:0006091	Generation of precursor metabolites and energy	31	82	0.0189
GO:0000132	Establishment of mitotic spindle orientation	4	5	0.0209
**MOLECULAR FUNCTION**				
GO:0004129	Cytochrome-c oxidase activity	6	7	0.002
GO:0004029	Aldehyde dehydrogenase (NAD) activity	6	8	0.0061
GO:0035255	Ionotropic glutamate receptor binding	5	6	0.0064
GO:0019905	Syntaxin binding	6	9	0.0142
GO:0046933	Proton-transporting ATP synthase activity, rotational mechanism	6	9	0.0142
GO:0070628	Proteasome binding	3	3	0.0192
GO:0072341	Modified amino acid binding	10	20	0.022
GO:0008026	ATP-dependent helicase activity	6	11	0.0471
GO:0030170	Pyridoxal phosphate binding	6	11	0.0471
GO:0016769	Transferase activity, transferring nitrogenous groups	4	6	0.0479
**MOLECULAR FUNCTION**				
GO:0005751	Mitochondrial respiratory chain complex IV	6	8	0.0065
GO:0030017	Sarcomere	20	47	0.0147
GO:0002080	Acrosomal membrane	3	3	0.0199
GO:0000275	Mitochondrial proton-transporting ATP synthase complex, catalytic core F(1)	4	5	0.0211
GO:0031201	SNARE complex	4	5	0.0211
GO:0005774	Vacuolar membrane	17	40	0.0241
GO:0016459	Myosin complex	7	13	0.0369
GO:0000922	Spindle pole	8	16	0.0424
GO:0090543	Flemming body	3	4	0.0635
GO:0005765	Lysosomal membrane	14	35	0.0655

*GO term ID (goId), GO term name (goName), number of differentially regulated proteins (countDE), total number of proteins associated with GO term (countAll) and p-value based on Elim pruning method (pv_elim)*.

**Table 4:** GO terms enriched in IWAT.

**Go Id**	**goName**	**countDE**	**countAll**	**pv_elim**
**BIOLOGICAL PROCESS**				
GO:0000381	Regulation of alternative mRNA splicing, via spliceosome	6	12	0.0029
GO:0032781	Positive regulation of ATPase activity	6	12	0.0029
GO:0032760	Positive regulation of tumor necrosis factor production	5	10	0.0066
GO:0042742	Defense response to bacterium	6	14	0.0073
GO:0043065	Positive regulation of apoptotic process	18	73	0.0074
GO:0000122	Negative regulation of transcription from RNA Polymerase II promoter	11	37	0.0083
GO:0000077	DNA damage checkpoint	3	4	0.0093
GO:0001960	Negative regulation of cytokine-mediated Signaling pathway	3	4	0.0093
GO:0002828	Regulation of type 2 immune response	3	4	0.0093
GO:0010799	Regulation of peptidyl-threonine phosphorylation	3	4	0.0093
**MOLECULAR FUNCTION**				
GO:0070573	Metallodipeptidase activity	3	3	0.0026
GO:0019955	Cytokine binding	4	6	0.0043
GO:0051015	Actin filament binding	12	40	0.0058
GO:0060590	ATPase regulator activity	4	7	0.009
GO:0005080	Protein kinase C binding	6	15	0.0112
GO:0003727	Single-stranded RNA binding	6	16	0.0159
GO:0004180	Carboxypeptidase activity	4	9	0.0258
GO:0036002	Pre-mRNA binding	4	9	0.0258
GO:0003697	Single-stranded DNA binding	5	14	0.0341
GO:0003725	Double-stranded RNA binding	6	19	0.0376
**CELLULAR COMPONENT**				
GO:0071013	Catalytic step 2 spliceosome	7	16	0.0032
GO:0031528	Microvillus membrane	4	6	0.0041
GO:0005811	Lipid droplet	8	24	0.0113
GO:0005681	Spliceosomal complex	10	22	0.0346
GO:0030315	T-tubule	4	10	0.037
GO:0005885	Arp2/3 protein complex	3	6	0.0372
GO:0016604	Nuclear body	11	46	0.0412
GO:0000776	Kinetochore	5	15	0.0437
GO:0016607	Nuclear speck	7	26	0.0548
GO:0022626	Cytosolic ribosome	12	54	0.0562

*GO term ID (goId), GO term name (goName), number of differentially regulated proteins (countDE), total number of proteins associated with GO term (countAll) and p-value based on Elim pruning method (pv_elim)*.

**Figure 5:**
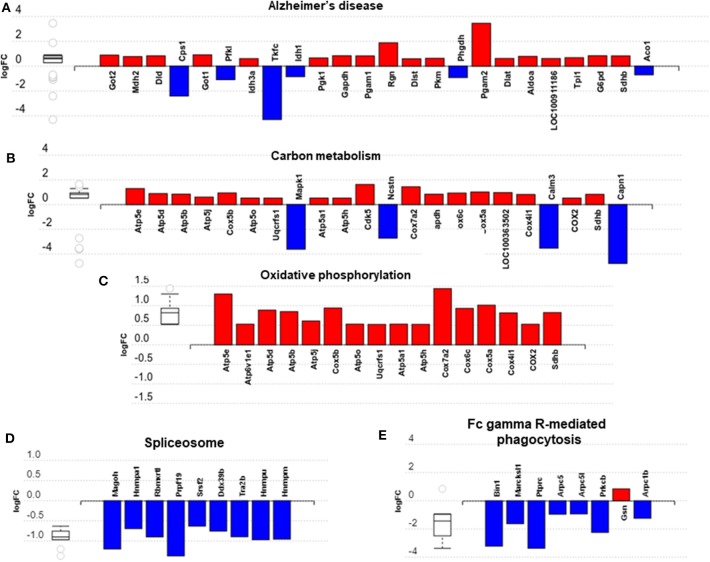
Overview of significantly impacted pathways in brown adipose tissue **(A–C)** and inguinal white adipose tissue **(D,E)**. **(A–E)** created with AdvaitaBio IPathwayGuide. *N* = 4/group, for analysis see methods section.

Impact analysis, which combines classical overrepresentation analysis with the perturbation of a pathway, highlighted several metabolic pathways modified by exercise (Supplementary Table 5) in BAT *carbon metabolism, Alzheimer's disease*, and *oxidative phosphorylation* (Figures 5A–C). In IWAT, the impacted pathways (Figures 5D,E) included the *spliceosome and Fc gamma R-mediated phagocytosis*.

### Characterization of the “Interactome” in Exercise-Trained Brown and White Adipose Tissues

**Figure 6:**
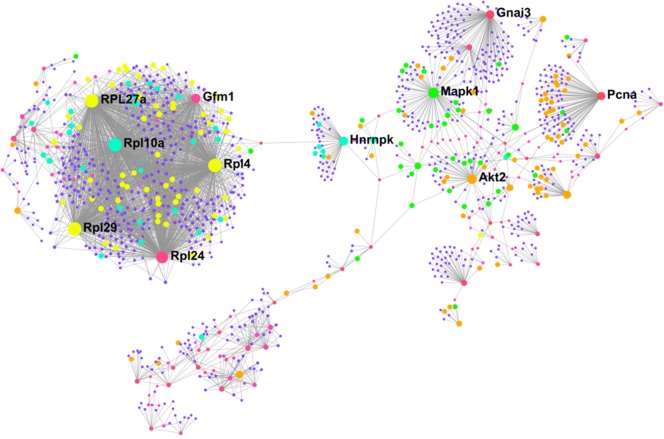
Protein-protein interaction network in BAT created using NetworkAnalyst showing hub proteins (bold) and proteins involved in *RNA binding* (yellow), *steroid dehydrogenase activity* (green), *neuropeptide hormone activity* (orange), and *transcription cofactor activity* (light blue). The pink/purple dots represent topological representation of other hub/seed proteins. *N* = 4/group, for analysis see methods section.

To better understand how our differentially altered proteins affect downstream signaling pathways, we characterized the “interactome” of BAT and IWAT through analysis of protein-protein interactions. NetworkAnalyst generated 28 sub-networks (i.e., the main “continent” and 27 “islands”) in BAT with the main network consisting of 1,091 proteins (Figure 6). Hub proteins (pink unless specified and labeled if high ranking) in this main network included the ribosomal proteins (i.e., RPL27a and RPL10a), the mitochondrial elongation factor GFM1, AKT Serine/Threonine Kinase 2 (Akt2) and mitogen-activated protein kinase 1 (MAPK1). Further analysis demonstrated interacting proteins (purple unless specified) in this main network enriched 30 biological processes (Supplementary Data PPI, Table 2) including *chromatin assembly or disassembly, developmental growth and muscle organ development* and 30 molecular functions (Figure 5) including *RNA binding* (yellow), *steroid dehydrogenase activity* (green), *neuropeptide hormone activity* (orange), and *transcription cofactor activity* (light blue).

**Figure 7:**
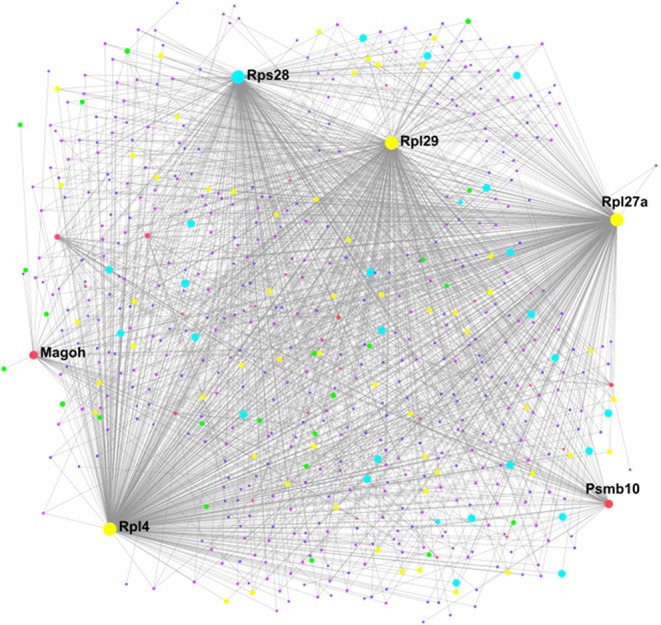
Protein-protein interaction network in WAT created using NetworkAnalyst showing hub proteins (bold) and proteins involved in *RNA binding (yellow), transcription cofactor activity (light blue)*, and nucleotide *binding (green)*. The pink/purple dots represent topological representation of other hub/seed proteins. *N* = 4/group, for analysis see methods section.

In IWAT, the “interactome” was smaller, consisting of 19 sub-networks (i.e., 1 “continent” and 18 “islands”) with the main network made up of 488 proteins (Figure 7). Hub proteins (pink unless specified and labeled if high ranking) in the main network again included multiple ribosomal proteins (i.e., RPL27a and RPL4) in addition to Proteasome subunit B10 (PSMB10) and the spliceosomal protein mago homolog, exon junction complex subunit (MAGOH). Further analysis demonstrated that the interacting proteins (purple unless specified) in this main network were involved in 6 biological processes (Supplementary data PPI, Supplementary Table 6) including *chromatin assembly or disassembly, sensory taste perception* and *RAS protein signal transduction* and 5 molecular functions (Figure 6) including *RNA binding (yellow), transcription cofactor activity (light blue)*, and nucleotide *binding (green)*.

## Discussion

The vast majority of studies investigating the function of BAT in rodents have been carried out at temperatures (i.e., c.20–22°C) which are well below thermoneutrality (i.e., c.28–33°C). These environmental differences have diverse effects on physiology, immunity and metabolism (19, 20). Whilst the use of thermoneutrality has been suggested as the optimal environment to mimic human physiology there is ongoing debate as to “how high” we should go (19, 33, 34). It was recently demonstrated that BAT from mice housed chronically at thermoneutrality and fed an obesogenic diet closer resembles human BAT with it suggested that this model of “physiologically humanized BAT” represents the ideal model of BAT physiology (22). Here, under these conditions (i.e., chronic thermoneutrality and diet induced obesity), we show exercise training induces an oxidative phenotype in BAT of obese animals that is associated with an enrichment of GO terms involved in skeletal muscle physiology, pathways associated with altered mitochondrial metabolism (i.e., oxidative phosphorylation) and increased lipid content. Unlike studies conducted at sub-thermoneutrality (18, 35), we show UCP1 mRNA is absent in IWAT of obese animals raised at thermoneutrality and is not induced with exercise training. Instead, IWAT in exercise trained animals exhibits a reduction in apoptotic proteins and perturbations in the spliceosomal pathway.

### A Thermogenic Response in PVAT and Adipocyte-to-Myocyte Switch in BAT

Despite no induction of thermogenic mRNA's in BAT, an upregulation of these markers in PVAT suggests an uncoupling of the response to exercise training in anatomically distinct BAT depots. A downregulation of thermogenic genes and “whitening” of thermogenic AT has previously been attributed to increases in core body temperature with exercise training (10). This would seem not to be the case, however, given an increase of these genes in PVAT which plays a critical role in heating blood prior to circulation (36). The physiological role of these alterations in PVAT is unclear but may represent depot-specific adaptations to exercise training which are potentially linked to cardiovascular alterations occurring with training. A “whitening” of BAT is evident however and is associated with a shift toward a muscle-like signature.

A myogenic signature in brown adipocytes was first established in 2007 when it was shown that the expression of myogenic genes in differentiating brown adipocytes was a characteristic that clearly distinguished them from white adipocytes (37). It was subsequently shown that both BAT and skeletal muscle derive from the same Pax7+ / Myf5+ progenitor cells and that the transcription factor PRDM16 drives the fate of these progenitors to committed brown adipocytes (38). Despite these shared characteristics, a definitive physiological role for these skeletal muscle associated proteins in BAT has not been demonstrated. In WAT, blockade of the β3-receptors induces myogenesis with (a) the emergence of MyoD+ mononucleated cells which undergo myogenesis even under adipogenic conditions and (b) the fusion of stromal cells which form multinucleated myotubes that “twitch” and express myosin-heavy chain (MHC) (39). A subset of UCP1+ beige adipocytes (c.15% of total beige adipocytes) in Myod1-CreERT2 reporter mice are derived from MyoD+ cells located adjacent to the microvasculature, and this “glycolytic” beige fat exhibits enhanced glucose metabolism compared to typical beige adipocytes.

Given that exercise training drives myogenesis in skeletal muscle, it may have a similar impact on BAT given their shared developmental origins and, whilst the prevalence of muscle cells (i.e., Pax7+) residing in BAT needs to be directly assessed in future studies an upregulation of markers including MYOD1, MYOG, and Myoglobin points toward an induction of myogenesis in BAT following exercise training. The enrichment of pathways involved in amino acid metabolism (i.e., *biosynthesis of amino acids*; *glycine, serine, threonine, arginine and proline metabolism*), and their known role in protein synthesis and muscle hypertrophy may point toward this and explain, in part, the increase in BAT mass though this could be attributed solely to increased lipid content. Mice lacking interferon regulatory factor 4 (IRF4) in BAT (BATI4KO) exhibit reduced exercise capacity at both low and high-intensity treadmill running, and display selective myopathy (40). Interscapular BAT of these exercise intolerant mice is characterized by an upregulation of genes governing skeletal muscle physiology, including MyoD1, troponin T1, and myostatin. This suggests that myogenesis in BAT may have an adverse effect on whole body physiology. Alongside the induction of muscle-related proteins was an upregulation of proteins involved in the *generation of precursor metabolites* and *energy and mitochondrial respiration*. Phosphoglycerate mutase 2 is a key muscle-specific enzyme in which mutations (i.e., muscle phosphoglycerate deficiency) cause tubular aggregates and exercise intolerance. An upregulation of this protein in BAT further strengthens the idea of a switch toward a muscle phenotype. An enrichment of multiple mitochondria associated metabolic pathways including OXPHOS further suggests that, despite no demonstrable impact on UCP1 in BAT, the metabolic activity of this tissue has potentially increased. On the other hand, no change in UCP1 mRNA alongside a significant increase in tissue mass could also suggest a net increase in the thermogenic capacity of the depot i.e., 50% more BAT expressing similar levels of UCP1. Structural and mechanical proteins, such as the actomyosin machinery in BAT, are crucial for the induction of oxidative metabolism and thermogenesis (41). BAT responds mechanically to adrenergic stimulation and actomyosin mediated tension with type-II myosins in particular facilitating uncoupled respiration. The induction of structural, and actomyosin related proteins seen in BAT with training could therefore facilitate an increased metabolic capacity in this tissue, which would support the upregulation of mitochondrial proteins. A downregulation of CASQ1, calcium sensor and regulator in the mitochondria and CALM3, a calcium binding protein suggest that exercise training may perturb calcium signaling in BAT which may be of particular functional importance for the SERCA2b-RyR2 pathway which can drive UCP1-independent thermogenesis (42–44). Whilst further work is needed to validate and corroborate this data, and to determine the functional role of specific proteins, we propose this muscle-like signature as a novel, UCP1-independent pathway through which exercise regulates BAT metabolism.

### A Role for Exercise in the Attenuation of Adipose Tissue Apoptosis

Whilst prior work has demonstrated an induction of thermogenic genes in WAT following exercise training, we show that UCP1 mRNA is absent in IWAT of animals raised at thermoneutrality from weaning. Instead, there are alterations to apoptotic and spliceosomal proteins. Dysregulated apoptotic processes are associated with AT inflammation and insulin resistance (45, 46). Here, we show one potential benefit of exercise training on IWAT is a downregulation of multiple proteins governing the “*positive regulation of apoptotic process.”* Bridging integrator 1, for instance, is a MYC proto-oncogene interacting factor that activates caspase-independent apoptosis in cancer cells, though its role in AT immunometabolism is unknown (47). Autophagy-related (ATG)7 is a ubiquitin activating enzyme which forms a complex with caspase-9 to cross-regulate autophagy and apoptosis (48). Adipocyte specific ATG7 k/o mice are lean with reduced fat mass, increased insulin sensitivity, an increase in BAT thermogenesis and are resistant to diet-induced obesity (49, 50). Other proteins of particular interest include MYB binding protein (MYBBP)1a and CD44. MYBBP1a is a SIRT7 interacting protein which regulates nucleolar stress and ribosome biogenesis that is increased in visceral AT of obese mice and negatively regulates adipogenesis (51, 52). Downregulation of MYBBP1a by exercise training may be a putative mechanism whereby physical activity and/or exercise regulates adipocyte number and size. Finally, CD44 mRNA is 3-fold higher in AT of insulin-resistant humans and correlates with CD68 and IL6, whilst CD44 k/o mice are phenotypically healthier and exhibit reduced AT inflammation (53, 54). These changes in apoptotic proteins occurred alongside an enrichment of proteins involved in the regulation of lipid droplets, including Lanosterol Synthase (LSS) and Carboxylesterase 1 (CES1) which regulate the synthesis of cholesterol, and the metabolism of cholesterol esters. Alongside a downregulation of Acyl-CoA Synthetase Long Chain Family Member 4 (ACSL4) this could point to exercise induced changes to the lipidome of WAT though how the lipidome of physiologically humanized animals following exercise training differs to animals at standard housing conditions remains to be determined (55).

Impact analysis demonstrated a number of significantly perturbed pathways. Epidermal growth factor (EGF) was the single protein differentially regulated in pathways including melanoma, phospholipase D signaling and PI3K-Akt signaling pathways. The role of EGF in adipogenesis is well-described with EGF receptor (ErbB1) abundance reduced in insulin resistant women with Type 2 diabetes. Importantly, EGF exerts insulin-like effects on adipocytes and skeletal muscle and this exercise-induced increase may be one way in which physical exercise potentiates insulin-sensitizing effects in these tissues (56). The most impacted pathway, however, was the spliceosome. This multi-megadalton ribonucleoprotein complex removes introns from RNA polymerase II transcripts (pre mRNAs) and is a crucial step in mRNA synthesis (57). Given that c.95% of genes are subject to alternative splicing, a downregulation of proteins involved in the spliceosome pathway would likely have major downstream effects on AT function, and may be driving the alterations observed in the proteome (58). Perturbation of the spliceosomal pathway was associated with an enrichment of the GO term “*regulation of RNA metabolic process*” in which 36 of 44 proteins were reduced. Why exercise training downregulates large numbers of proteins involved in pre-mRNA synthesis and in RNA metabolism is, however, unclear and merits further investigation.

### Characterizing the Exercise Interactome in BAT and WAT

Analysis of protein-protein interactions is an important step in understanding the communication between proteins and identification of putative signaling pathways occurring in specific tissues following an intervention. Here, we have identified multiple ribosomal proteins (i.e., RPL27a and RPL10a), AKT2 and MAPK1 as important hub proteins controlling regulation of chromatin assembly, muscle organ development and steroid hormone dehydrogenase activity in exercised BAT. It has been suggested that increased ribosomal biogenesis plays a major role in skeletal muscle hypertrophy and though a number of these ribosomal proteins were downregulated (i.e., RPL21, RPL29, RPL15, RPL10a, RPL27a, and RPL4) we propose that these specific ribosomal hub proteins may play an important role in the shift toward a muscle-like signature in BAT through their interacting proteins (59). Further, ribosomal proteins are regulated by growth factors, and that IGF1 specifically regulates muscle hypertrophy through both the PI3K-AKT-mTOR and MAPK signaling pathways, with the latter playing a key role in regulating ribosomal biogenesis (59–61). These adaptations could also be a response to physiological stress, with ribosomal proteins involved in numerous functions beyond the ribosome including immune signaling, inflammation, and development (62). It is not clear at present whether the changes to ribosomal proteins in BAT impacts on ribosomal biogenesis or, extra ribosomal functions however, proteostasis is essential for the adaptation of BAT, and mice to cold and obesity and the same may apply to exercise training. PCNA, is a cell cycle protein, whose upregulation suggests satellite cells have entered the cell cycle (63, 64). That AKT2, MAPK1, and PCNA are identified as hub proteins suggests that both of them, and the proteins interacting with them, could be important to the induction of muscle-related proteins in BAT. These proteins and their downstream targets offer novel insight into the induction of muscle-related proteins in BAT and the regulation of BAT physiology with exercise. The identification of ribosomal proteins as both hub and interacting proteins in WAT suggests they are also important in the general regulation of adipose tissue physiology by exercise training, though their role in WAT is less clear. To date, no functional role for these hub proteins in AT has been shown though the identification of MAGOH as a hub protein suggests it may play a key role in the regulation of the spliceosome pathway. Functional studies of these hub proteins and signaling pathways in AT is needed to better understand how exercise regulates WAT biology.

### Strengths, Limitations, and Future Perspectives

We acknowledge that there are several limitations to this work. First, despite showing a trend toward reduced energy intake and RER we recognize that at ~500–600 g these animals are likely to be near the maximum capacity of the metabolic cages, and that home cage systems would obtain more accurate physiological data (65). Second, whether the effects we see here occur in lean animals remains to be established. Whilst not directly comparable, recent work by Raun et al. and McKie et al. (66, 67) shows an attenuated effect of exercise on thermogenic genes in lean 8 to 10 week-old mice acclimated to Tn for just 1–2 weeks. Whilst it needs testing, it is feasible to suggest the lack of effect on UCP1 seen here would also be absent in lean animals raised at Tn from weaning given the extended time spent at thermoneutrality. Thermoneutrality also impacts on running volume, the diurnal rhythm of RER and attenuates metabolic adaptations including glucose homeostasis and insulin action and it could also be suggested that the complete lack of an effect on multiple metabolites and physiological parameters seen here could be attributed to chronic thermoneutrality from weaning (66, 67). It will also need to be determined whether the effects we see here are applicable to other modalities of exercise training such as wheel and treadmill running. However, given “browning” is not restricted to a single exercise type it is not a given that other modalities would induced thermogenic genes in IWAT (18). Further, we opted for swim training as there is a reduced risk of injuries (i.e., foot/leg) and, whilst there is evidence to suggest that rats “float” in water when air is caught in their fur we noted that fur thickness was greatly reduced compared to what we typically see in animals of a similar age housed at 20°C. Though we did not place the control group in shallow water to control for any specific effect of water temperature, or stress, on thermogenesis this would be unlikely to mimic many of the effects seen in fully submerged, swimming animals but is worthy of future consideration. Furthermore, adipose tissue proteomics was a secondary, unbiased analysis in the absence of a thermogenic effect and, whilst validation is needed in larger studies, we consider this to be an important data set given it is the first work to analyse the proteome in animals raised, and trained under these conditions.

## Conclusion

We propose that BAT is dormant at thermoneutrality and that exercise training drives a muscle-like signature. The physiological relevance of this adaptation is unclear and BAT-muscle crosstalk, in particular, merits further investigation. Meanwhile, WAT exhibits a reduction in apoptotic proteins and a wholesale downregulation of proteins involved in pre-mRNA synthesis and RNA metabolism.

## Data Availability Statement

The datasets used and analyzed during the current study are available from the corresponding author on reasonable request.

## Ethics Statement

The animal study was reviewed and approved by University of Nottingham Animal Welfare and Ethical Review Board.

## Author Contributions

PA, HB, and MS conceived the study and attained the funding. PA and MS developed and designed the experiments. PA, JL, IL, AM, IB, RC, and DB performed the experiments. PA, AM, and DB analyzed the data. PA and MS wrote the paper which was revised critically by DB, HB, FE, and JL for important intellectual content. All authors read and approved the final manuscript.

### Conflict of Interest

The authors declare that the research was conducted in the absence of any commercial or financial relationships that could be construed as a potential conflict of interest.
