# Supplementary material for: Exercise Training in Obese Rats Does Not Induce Browning at Thermoneutrality and Induces a Muscle-Like Signature in Brown Adipose Tissue
Source: Front Endocrinol (Lausanne). 2020 Mar 20;11:97. doi: 10.3389/fendo.2020.00097 (PMC7099615; doi:10.3389/fendo.2020.00097)
Supplement: Supplementary file 1 [file Data_Sheet_1.docx]

## Supplementary data

| **Supplementary Table 1:** Serum parameters, liver weight and hepatic TG | | |
| --- | --- | --- |
|  | **HFD** | **Ex** |
| **Glucose (mg/dl)** | 109.4 ± 7.25 | 103.8 ± 3.5 |
| **Insulin (ng/ml)** | 1 ± 0.45 | 0.85 ± 0.12 |
| **Homa-IR** | 6.8 ± 2.9 | 6 ± 1 |
| **Triglycerides (TG, mg/dl)** | 63 ± 41 | 60.6 ± 19 |
| **NEFA (mmol/L)** | 0.39 ± 0.04 | 0.34 ± 0.03 |
| **Liver weight (g)** | 18.1 ± 1.8 | 15.7 ± 0.95 |
| **Hepatic TG (mg/dl)** | 21 ± 4.3 | 16.5 ± 4.2 |

| **Supplementary Table 2:** Differentially regulated proteins in BAT | | | |
| --- | --- | --- | --- |
| **symbol** | **entrez** | **logfc** | **adjpv** |
| Picalm | 89816 | -1.78 | 0.000004 |
| Scoc | 364981 | -1.85 | 0.000074 |
| Trim72 | 365377 | 0.77 | 0.000139 |
| Arf1 | 64310 | -2.73 | 0.000150 |
| Rbbp9 | 29459 | -1.80 | 0.000168 |
| Rps26 | 27139 | -0.57 | 0.000217 |
| Pgrmc2 | 361940 | -1.29 | 0.000270 |
| Atp5e | 245958 | 1.30 | 0.000345 |
| Ruvbl1 | 65137 | -2.89 | 0.000414 |
| Serpinb1a | 291091 | -0.68 | 0.000424 |
| Rnmt | 291534 | 2.86 | 0.000447 |
| Nup35 | 295692 | 1.48 | 0.000704 |
| Ggcx | 81716 | 0.76 | 0.000880 |
| Rpl21 | 79449 | -3.79 | 0.000976 |
| Farsa | 288917 | -2.24 | 0.001057 |
| Pon1 | 84024 | 1.27 | 0.001358 |
| Vars | 25009 | 0.91 | 0.001382 |
| Slc3a2 | 50567 | 1.68 | 0.001386 |
| Acox1 | 50681 | -1.05 | 0.001481 |
| Rab7a | 29448 | -0.56 | 0.001598 |
| Akr1b7 | 116463 | 1.15 | 0.001708 |
| Ybx1 | 500538 | -0.56 | 0.001734 |
| Rpl27a | 293418 | -1.67 | 0.001752 |
| Gdi1 | 25183 | -0.57 | 0.001925 |
| Lypla1 | 25514 | 0.78 | 0.001931 |
| Ckb | 24264 | 1.15 | 0.001942 |
| Twf1 | 315265 | -1.78 | 0.002050 |
| Ilk | 170922 | -0.87 | 0.002684 |
| F11r | 116479 | 4.34 | 0.002740 |
| Myl12a | 501203 | 0.86 | 0.002753 |
| Sec13 | 297522 | 2.87 | 0.003027 |
| Smu1 | 117541 | -2.69 | 0.003096 |
| Aldh16a1 | 361571 | 2.47 | 0.003332 |
| Arf4 | 79120 | -0.84 | 0.003462 |
| Mrfap1 | 282585 | 3.02 | 0.003490 |
| Mat2a | 171347 | -1.05 | 0.003748 |
| LOC100359498 | 100359498 | -3.78 | 0.003871 |
| Myl9 | 296313 | 1.38 | 0.004054 |
| Vdac1 | 83529 | 0.69 | 0.004226 |
| Atp6v1e1 | 297566 | 0.53 | 0.004351 |
| Fabp3 | 79131 | -0.95 | 0.004662 |
| Csnk2a1 | 116549 | -0.83 | 0.004812 |
| Safb | 64196 | -1.90 | 0.005003 |
| Tst | 25274 | -0.58 | 0.005274 |
| Kirrel1 | 310695 | 2.09 | 0.005493 |
| Gpd1 | 60666 | 0.55 | 0.005949 |
| Psmb8 | 24968 | -0.79 | 0.006059 |
| Flot2 | 83764 | 2.59 | 0.006085 |
| Ssbp1 | 54304 | 0.84 | 0.006147 |
| Gnl1 | 309593 | 0.87 | 0.006282 |
| Steap4 | 499991 | -3.82 | 0.006402 |
| Hnrnpl | 80846 | -2.97 | 0.006430 |
| Prdx4 | 85274 | -1.51 | 0.006529 |
| Gnb2 | 81667 | -2.07 | 0.007102 |
| Cnbp | 64530 | 2.51 | 0.007689 |
| Adrm1 | 65138 | -0.79 | 0.007875 |
| Lipe | 25330 | -0.74 | 0.008475 |
| Gnai1 | 25686 | -3.93 | 0.009086 |
| Got2 | 25721 | 0.89 | 0.009448 |
| Oxct1 | 690163 | 1.08 | 0.009519 |
| Mdh2 | 81829 | 0.77 | 0.010040 |
| Pcyt2 | 89841 | 2.33 | 0.010292 |
| Atp5d | 245965 | 0.89 | 0.010468 |
| Ccdc93 | 304743 | -0.85 | 0.010497 |
| Acy1 | 300981 | -0.86 | 0.010542 |
| Enpp3 | 54410 | 1.30 | 0.010658 |
| Fhl1 | 25177 | 3.00 | 0.010919 |
| Atp5b | 171374 | 0.85 | 0.011316 |
| Rpl29 | 29283 | -2.55 | 0.011633 |
| Ssr3 | 81784 | 2.93 | 0.011777 |
| Jpt1 | 287828 | 2.32 | 0.011813 |
| Atp5j | 94271 | 0.61 | 0.012402 |
| Mapre1 | 114764 | 3.45 | 0.012522 |
| Rsl24d1 | 363099 | 0.79 | 0.012559 |
| Hnrnpk | 117282 | -0.52 | 0.013031 |
| Ak1 | 24183 | 1.51 | 0.013130 |
| Prdx3 | 64371 | 0.95 | 0.013174 |
| Caprin1 | 362173 | -2.55 | 0.013285 |
| Lrrc59 | 287633 | -3.26 | 0.013322 |
| Pnpo | 64533 | 3.73 | 0.013417 |
| Mfge8 | 25277 | 2.41 | 0.013605 |
| Ikbip | 314730 | 0.95 | 0.013649 |
| Parva | 57341 | -1.48 | 0.013685 |
| Canx | 29144 | -0.67 | 0.013691 |
| Actr2 | 289820 | -0.99 | 0.013761 |
| RGD1564664 | 499839 | -2.64 | 0.013794 |
| Scgb2a1 | 25010 | -1.17 | 0.014211 |
| Impact | 497198 | -1.83 | 0.014980 |
| Ptk2 | 25614 | -1.62 | 0.015614 |
| LOC103689947 | 103689947 | -0.96 | 0.015631 |
| Eepd1 | 315500 | 1.78 | 0.016096 |
| Hmgn5b | 681284 | -2.24 | 0.016327 |
| Snap23 | 64630 | -0.97 | 0.016460 |
| Ppif | 282819 | 1.13 | 0.016485 |
| Stmn1 | 29332 | 1.08 | 0.016813 |
| Nedd4 | 25489 | -1.15 | 0.017773 |
| Rabep2 | 80754 | 2.46 | 0.018227 |
| Rwdd1 | 259218 | 0.79 | 0.018511 |
| Ostf1 | 259275 | -2.88 | 0.019652 |
| Cox5b | 94194 | 0.94 | 0.020079 |
| Acadsb | 25618 | -1.95 | 0.020232 |
| Dld | 298942 | 0.83 | 0.020561 |
| Akt2 | 25233 | -1.19 | 0.021849 |
| Tomm22 | 300075 | 2.52 | 0.021870 |
| Dnm2 | 25751 | -1.34 | 0.022571 |
| LOC100909840 | 100909840 | 0.57 | 0.026078 |
| Ufc1 | 445268 | -0.63 | 0.026505 |
| Pecam1 | 29583 | 3.40 | 0.027233 |
| Rab2a | 65158 | -0.55 | 0.028258 |
| Fkbp9 | 297123 | -2.09 | 0.028266 |
| Ddx46 | 245957 | 2.43 | 0.028396 |
| Tmed9 | 361207 | 1.91 | 0.029775 |
| Retsat | 246298 | -1.78 | 0.030198 |
| Nras | 24605 | -0.52 | 0.031192 |
| Pdlim5 | 64353 | 0.87 | 0.031521 |
| Aldh2 | 29539 | -0.52 | 0.032339 |
| Samm50 | 300111 | 2.52 | 0.032735 |
| Pcyox1 | 246302 | 1.84 | 0.033267 |
| Rps18 | 294282 | -0.60 | 0.033608 |
| Tsc22d1 | 498545 | -2.60 | 0.034233 |
| Gsta4 | 300850 | -0.62 | 0.035195 |
| Atp5o | 192241 | 0.53 | 0.035395 |
| Cps1 | 497840 | -2.41 | 0.036115 |
| Rps25 | 122799 | -0.52 | 0.036500 |
| Paics | 140946 | -1.21 | 0.037037 |
| Ppp1r14b | 259225 | 2.90 | 0.037840 |
| Uqcrfs1 | 291103 | 0.52 | 0.037957 |
| Aoc3 | 29473 | -1.10 | 0.038141 |
| Oat | 64313 | -2.43 | 0.039004 |
| Scarb1 | 25073 | 2.61 | 0.039553 |
| Sardh | 114123 | -0.75 | 0.039737 |
| Gfm1 | 114017 | 1.64 | 0.041349 |
| Tmc5 | 365360 | 5.71 | 0.042368 |
| Asns | 25612 | -2.47 | 0.042482 |
| Msh2 | 81709 | 0.68 | 0.042956 |
| Rpl22 | 81768 | 4.13 | 0.043960 |
| Add3 | 25230 | 3.15 | 0.045349 |
| Elavl1 | 363854 | -0.77 | 0.045888 |
| Ddx39a | 89827 | -1.82 | 0.046585 |
| Cdipt | 192260 | 1.56 | 0.047053 |
| Arcn1 | 300674 | -0.87 | 0.047360 |
| RGD1303003 | 294326 | 1.05 | 0.049330 |
| Mb | 59108 | 5.31 | 0.052570 |
| Got1 | 24401 | 0.92 | 0.052958 |
| Mapk1 | 116590 | -3.62 | 0.053239 |
| Cryab | 25420 | 1.48 | 0.053648 |
| Atp5a1 | 65262 | 0.53 | 0.053811 |
| Sult1a1 | 83783 | -1.26 | 0.054189 |
| Sfswap | 304431 | 1.71 | 0.055680 |
| Timm9 | 171139 | 0.97 | 0.055722 |
| Pfkl | 25741 | -1.09 | 0.056291 |
| Mgst1 | 171341 | -0.72 | 0.056293 |
| Smarcad1 | 312398 | -1.80 | 0.056638 |
| Khdrbs1 | 117268 | -1.85 | 0.056680 |
| C1qb | 29687 | -2.19 | 0.057651 |
| Pdcd6ip | 501083 | -3.60 | 0.059487 |
| Ssrp1 | 81785 | 2.00 | 0.060232 |
| Pafah1b1 | 83572 | -1.99 | 0.060508 |
| Ubr4 | 313658 | -1.28 | 0.062780 |
| Manf | 315989 | 0.66 | 0.063485 |
| Psmb5 | 29425 | 2.20 | 0.064190 |
| Pdxk | 83578 | 0.62 | 0.065427 |
| Cdkl3 | 60396 | -3.87 | 0.066289 |
| LOC100364435 | 100364435 | 0.74 | 0.066647 |
| Aebp1 | 305494 | -2.30 | 0.067465 |
| Nubp2 | 287125 | -1.40 | 0.069635 |
| Sar1b | 287276 | -0.63 | 0.071615 |
| Casq1 | 686019 | -4.64 | 0.073187 |
| LOC100360117 | 100360117 | -1.20 | 0.073724 |
| Prdx5 | 113898 | 0.71 | 0.075140 |
| Emd | 25437 | 0.64 | 0.075803 |
| Hsp90ab1 | 301252 | -0.70 | 0.076030 |
| LOC103689992 | 103689992 | -2.04 | 0.076706 |
| Napa | 140673 | -1.09 | 0.079278 |
| Ctnnb1 | 84353 | 2.02 | 0.080415 |
| Rpl4 | 64302 | -1.17 | 0.081163 |
| Sh3gl1 | 81922 | -0.55 | 0.082156 |
| Pcna | 25737 | 1.76 | 0.082465 |
| Hprt1 | 24465 | -0.74 | 0.084325 |
| Pir | 363465 | 0.82 | 0.085585 |
| Ugt1a6 | 113992 | -1.81 | 0.085618 |
| Pdia4 | 116598 | -0.63 | 0.089608 |
| Cttn | 60465 | 1.51 | 0.089611 |
| St13 | 81800 | -0.72 | 0.089875 |
| Cops4 | 360915 | -1.96 | 0.089984 |
| Aldh1l1 | 64392 | -0.59 | 0.092890 |
| Idh3a | 114096 | 0.61 | 0.093937 |
| Top2a | 360243 | -0.59 | 0.094134 |
| Itga7 | 81008 | -0.54 | 0.094593 |
| Rpl35 | 296709 | 4.40 | 0.096043 |
| Eif3d | 362952 | -1.44 | 0.096584 |
| Hmgcs1 | 29637 | -3.66 | 0.097233 |
| Dctn1 | 29167 | -1.90 | 0.098515 |
| Atp5h | 641434 | 0.52 | 0.099922 |
| Gnai3 | 25643 | -2.11 | 0.100267 |
| Tkfc | 361730 | -4.30 | 0.100892 |
| Bsg | 25246 | 0.67 | 0.101244 |
| Gstm2 | 24424 | -0.91 | 0.101246 |
| Ddx1 | 84474 | 0.88 | 0.102323 |
| F13a1 | 60327 | -1.92 | 0.102787 |
| Txnrd1 | 58819 | -1.75 | 0.104505 |
| Hspb6 | 192245 | 5.06 | 0.104759 |
| Pdcd4 | 64031 | -1.97 | 0.106124 |
| Hnrnpa3 | 362152 | -0.95 | 0.107001 |
| Vapb | 60431 | -1.71 | 0.107677 |
| Wdr1 | 360950 | -0.51 | 0.108763 |
| Idh1 | 24479 | -0.85 | 0.108862 |
| Rdh16 | 299511 | -3.14 | 0.109968 |
| Cdk5 | 140908 | 1.63 | 0.110065 |
| Ykt6 | 64351 | 0.60 | 0.110104 |
| Cald1 | 25687 | 2.01 | 0.111411 |
| Hspa13 | 29734 | -2.13 | 0.113215 |
| LOC684988 | 684988 | -0.70 | 0.113256 |
| Nes | 25491 | 0.82 | 0.113860 |
| Fcgrt | 29558 | -2.38 | 0.114757 |
| Ncstn | 289231 | -2.73 | 0.115743 |
| Eif4a2 | 303831 | -0.92 | 0.122267 |
| Rpl10a | 81729 | -1.92 | 0.122600 |
| Zw10 | 363059 | 2.02 | 0.124740 |
| Orm1 | 24614 | 1.14 | 0.127099 |
| Nme1 | 191575 | -2.79 | 0.128373 |
| LOC100364457 | 100364457 | 3.51 | 0.131740 |
| Ndel1 | 170845 | 2.18 | 0.131759 |
| Rpl15 | 245981 | -2.09 | 0.132344 |
| Psmb9 | 24967 | 0.61 | 0.135388 |
| Snd1 | 64635 | 1.85 | 0.136424 |
| LOC108348260 | 108348260 | 3.39 | 0.138472 |
| Hspd1 | 63868 | 1.00 | 0.138850 |
| Park7 | 117287 | 0.57 | 0.139941 |
| Timm8b | 64372 | 0.88 | 0.141464 |
| Myo1b | 117057 | 2.10 | 0.141822 |
| Pgk1 | 24644 | 0.67 | 0.141840 |
| Pcyt1a | 140544 | -1.41 | 0.142742 |
| Crip1 | 691657 | 2.15 | 0.143393 |
| Wdr61 | 363064 | 1.74 | 0.143844 |
| Bag6 | 94342 | -3.54 | 0.144015 |
| Cox7a2 | 29507 | 1.44 | 0.144661 |
| Rad23b | 298012 | -2.45 | 0.146032 |
| B2m | 24223 | -0.52 | 0.146645 |
| Stoml2 | 298203 | 0.64 | 0.147220 |
| C1qc | 362634 | -3.57 | 0.147757 |
| Esyt1 | 29579 | -0.65 | 0.147895 |
| Letm1 | 305457 | 0.95 | 0.147977 |
| Aacs | 65984 | 3.31 | 0.148642 |
| Cpne1 | 362249 | -2.06 | 0.149046 |
| Npas4 | 266734 | -2.93 | 0.149382 |
| Ace | 24310 | -1.49 | 0.149461 |
| Rab6a | 84379 | 0.66 | 0.152650 |
| Gapdh | 24383 | 0.84 | 0.152864 |
| Ppp2r2a | 117104 | 3.43 | 0.153227 |
| Mesd | 308796 | 1.38 | 0.155213 |
| Arl6ip5 | 66028 | 5.19 | 0.156194 |
| Pgam1 | 24642 | 0.83 | 0.157461 |
| Vps26a | 361846 | -0.58 | 0.159039 |
| Fahd2a | 296131 | 0.57 | 0.159065 |
| Clic4 | 83718 | -1.92 | 0.159544 |
| Hnrnpdl | 305178 | -0.74 | 0.160951 |
| Actg2 | 25365 | -0.89 | 0.161060 |
| Rgn | 25106 | 1.88 | 0.162047 |
| Tollip | 361677 | -2.64 | 0.162653 |
| Abhd5 | 316122 | -0.71 | 0.164264 |
| Dlst | 299201 | 0.60 | 0.166679 |
| Ist1 | 307833 | -0.97 | 0.166705 |
| Pdap1 | 64527 | 0.60 | 0.166984 |
| Pck1 | 362282 | -1.16 | 0.168424 |
| Ifi30 | 290644 | 1.11 | 0.170114 |
| Hsdl2 | 313200 | 0.67 | 0.170726 |
| Hadh | 113965 | 0.54 | 0.171038 |
| Cox6c | 54322 | 0.93 | 0.171268 |
| Cpne9 | 297516 | -1.95 | 0.172196 |
| Pkm | 25630 | 0.63 | 0.172311 |
| Tmsb4x | 81814 | 1.15 | 0.173148 |
| Mpc2 | 100359982 | 1.78 | 0.175528 |
| Phgdh | 58835 | -0.92 | 0.175599 |
| Vps4a | 246772 | -1.33 | 0.175994 |
| Pgam2 | 24959 | 3.45 | 0.176098 |
| Cox5a | 252934 | 1.01 | 0.176128 |
| Sptan1 | 64159 | -1.13 | 0.176749 |
| Wdr77 | 310769 | 2.02 | 0.177932 |
| Adprh | 25371 | -1.62 | 0.183279 |
| Mlec | 304543 | -2.97 | 0.185597 |
| Ecm1 | 116662 | 1.51 | 0.186983 |
| Slc2a4 | 25139 | -0.94 | 0.188195 |
| P4ha1 | 64475 | -1.73 | 0.188534 |
| Trap1 | 287069 | 0.88 | 0.189767 |
| Rnpep | 81761 | 3.76 | 0.189955 |
| Gusb | 24434 | 1.64 | 0.190759 |
| Ik | 291659 | 1.88 | 0.191120 |
| Eif3i | 682390 | 0.81 | 0.193399 |
| Rpl24 | 64307 | 0.98 | 0.195425 |
| Acaa2 | 170465 | 0.70 | 0.195993 |
| Actn1 | 81634 | 2.39 | 0.196673 |
| Psmb7 | 85492 | -3.46 | 0.198010 |
| Fads2 | 83512 | -3.03 | 0.198071 |
| Eif3b | 288516 | 2.49 | 0.198331 |
| Dpysl3 | 25418 | -1.70 | 0.198904 |
| Dlat | 81654 | 0.62 | 0.200109 |
| Csad | 60356 | -1.42 | 0.204017 |
| Prpf19 | 246216 | -1.70 | 0.204243 |
| Aldh9a1 | 64040 | -0.52 | 0.206158 |
| Tpm1 | 24851 | 1.21 | 0.206304 |
| Tnni2 | 29389 | 4.09 | 0.207299 |
| Lamtor1 | 308869 | -2.14 | 0.207568 |
| Xpnpep1 | 170751 | -1.47 | 0.207898 |
| LOC108350501 | 108350501 | -2.43 | 0.210098 |
| Ces1c | 24346 | -0.58 | 0.210303 |
| Prep | 83471 | -1.55 | 0.210574 |
| LOC100363502 | 100363502 | 0.97 | 0.210978 |
| Cav2 | 363425 | -1.87 | 0.212581 |
| Rab14 | 94197 | 1.30 | 0.212835 |
| Cox4i1 | 29445 | 0.82 | 0.213019 |
| Mylpf | 24584 | 3.42 | 0.213227 |
| Dhfr | 24312 | -1.32 | 0.213228 |
| Calm3 | 24244 | -3.54 | 0.215148 |
| Aldh3a2 | 65183 | -0.91 | 0.215474 |
| Myl6l | 362816 | 0.62 | 0.217432 |
| COX2 | 26198 | 0.53 | 0.218492 |
| Sod1 | 24786 | 0.63 | 0.218695 |
| Aldoa | 24189 | 0.79 | 0.222836 |
| LOC100359574 | 100359574 | 2.21 | 0.224398 |
| Ddah2 | 294239 | -0.88 | 0.226279 |
| Ninj1 | 25338 | 2.01 | 0.226636 |
| S100a10 | 81778 | -1.20 | 0.227322 |
| Ugdh | 83472 | 2.11 | 0.227992 |
| LOC100911186 | 100911186 | 0.62 | 0.228664 |
| Tpi1 | 24849 | 0.69 | 0.229888 |
| Cand1 | 117152 | -2.49 | 0.230824 |
| Timeless | 83508 | 3.85 | 0.231260 |
| Rps8 | 65136 | 1.29 | 0.232789 |
| Cavin3 | 85332 | -1.94 | 0.233017 |
| Snrpn | 81781 | 2.58 | 0.234247 |
| Cd48 | 245962 | 0.85 | 0.234946 |
| Eef1a1 | 171361 | -0.57 | 0.234976 |
| G6pd | 24377 | 0.84 | 0.235912 |
| Marcksl1 | 81520 | 2.83 | 0.236207 |
| Sdhb | 298596 | 0.83 | 0.239584 |
| Pdlim3 | 114108 | 4.15 | 0.240689 |
| Sars | 266975 | -3.39 | 0.242342 |
| Myh4 | 360543 | 3.53 | 0.242512 |
| Enpp1 | 85496 | 2.11 | 0.243007 |
| Ccdc43 | 360637 | -3.78 | 0.243166 |
| Map4 | 367171 | 0.61 | 0.243767 |
| Ccdc22 | 317381 | -1.44 | 0.244454 |
| Capn1 | 29153 | -4.77 | 0.245231 |
| Cygb | 170520 | -1.53 | 0.245820 |
| Gnas | 24896 | -1.05 | 0.246029 |
| Sec22b | 310710 | -2.77 | 0.246288 |
| Dhx30 | 367172 | 0.89 | 0.246383 |
| Aco1 | 50655 | -0.70 | 0.246476 |
| Tcp1 | 24818 | 3.51 | 0.246974 |
| Lamb2 | 25473 | -2.82 | 0.247789 |
| Aldh1a1 | 24188 | 2.66 | 0.249144 |
| Ca5b | 302669 | -1.41 | 0.249964 |

| **Supplementary Table 3:** GO terms enriched in BAT | | | | |
| --- | --- | --- | --- | --- |
| **goId** | **goName** | **countDE** | **countAll** | **pv_elim** |
| **Biological Process** | |  |  |  |
| GO:0003151 | outflow tract morphogenesis | 4 | 4 | 0.0053 |
| GO:0006103 | 2-oxoglutarate metabolic process | 6 | 8 | 0.0064 |
| GO:0051304 | chromosome separation | 5 | 6 | 0.0067 |
| GO:0007093 | mitotic cell cycle checkpoint | 6 | 9 | 0.0148 |
| GO:0014075 | response to amine | 6 | 9 | 0.0148 |
| GO:0014044 | Schwann cell development | 5 | 7 | 0.0182 |
| GO:0018198 | peptidyl-cysteine modification | 5 | 7 | 0.0182 |
| GO:2000273 | positive regulation of receptor activity | 5 | 7 | 0.0182 |
| GO:0006091 | generation of precursor metabolites and energy | 31 | 82 | 0.0189 |
| GO:0000132 | establishment of mitotic spindle orientation | 4 | 5 | 0.0209 |
| GO:0006071 | glycerol metabolic process | 4 | 5 | 0.0209 |
| GO:2000008 | regulation of protein localization to cell surface | 4 | 5 | 0.0209 |
| GO:0006906 | vesicle fusion | 8 | 15 | 0.0272 |
| GO:0006165 | nucleoside diphosphate phosphorylation | 12 | 26 | 0.0276 |
| GO:0015986 | ATP synthesis coupled proton transport | 9 | 13 | 0.0279 |
| GO:0055081 | anion homeostasis | 6 | 10 | 0.0287 |
| GO:0071482 | cellular response to light stimulus | 6 | 10 | 0.0287 |
| GO:0090317 | negative regulation of intracellular protein transport | 6 | 10 | 0.0287 |
| GO:0006090 | pyruvate metabolic process | 13 | 29 | 0.0289 |
| GO:0043087 | regulation of GTPase activity | 14 | 32 | 0.0297 |
| GO:0030516 | regulation of axon extension | 9 | 18 | 0.0313 |
| GO:1902600 | hydrogen ion transmembrane transport | 16 | 26 | 0.0335 |
| GO:0007519 | skeletal muscle tissue development | 10 | 21 | 0.0343 |
| GO:0030278 | regulation of ossification | 7 | 13 | 0.0365 |
| GO:0034308 | primary alcohol metabolic process | 7 | 13 | 0.0365 |
| GO:0048538 | thymus development | 5 | 8 | 0.0378 |
| GO:0043603 | cellular amide metabolic process | 62 | 191 | 0.0443 |
| GO:0046364 | monosaccharide biosynthetic process | 9 | 19 | 0.0457 |
| GO:1990138 | neuron projection extension | 15 | 26 | 0.0466 |
| GO:0055085 | transmembrane transport | 45 | 109 | 0.0469 |
| GO:0051345 | positive regulation of hydrolase activity | 29 | 81 | 0.048 |
| GO:0006081 | cellular aldehyde metabolic process | 10 | 22 | 0.0482 |
| GO:0043010 | camera-type eye development | 10 | 22 | 0.0482 |
| GO:0006739 | NADP metabolic process | 6 | 11 | 0.0491 |
| GO:0009308 | amine metabolic process | 4 | 6 | 0.0494 |
| GO:0032770 | positive regulation of monooxygenase activity | 4 | 6 | 0.0494 |
| GO:0050771 | negative regulation of axonogenesis | 4 | 6 | 0.0494 |
| GO:0090151 | establishment of protein localization to mitochondrial membrane | 4 | 6 | 0.0494 |
| **Molecular Function** | |  |  |  |
| GO:0004129 | cytochrome-c oxidase activity | 6 | 7 | 0.002 |
| GO:0004029 | aldehyde dehydrogenase (NAD) activity | 6 | 8 | 0.0061 |
| GO:0035255 | ionotropic glutamate receptor binding | 5 | 6 | 0.0064 |
| GO:0019905 | syntaxin binding | 6 | 9 | 0.0142 |
| GO:0046933 | proton-transporting ATP synthase activity, rotational mechanism | 6 | 9 | 0.0142 |
| GO:0070628 | proteasome binding | 3 | 3 | 0.0192 |
| GO:0072341 | modified amino acid binding | 10 | 20 | 0.022 |
| GO:0008026 | ATP-dependent helicase activity | 6 | 11 | 0.0471 |
| GO:0030170 | pyridoxal phosphate binding | 6 | 11 | 0.0471 |
| GO:0016769 | transferase activity, transferring nitrogenous groups | 4 | 6 | 0.0479 |
| GO:0019888 | protein phosphatase regulator activity | 4 | 6 | 0.0479 |
| GO:0042805 | actinin binding | 4 | 6 | 0.0479 |
| **Cellular Component** | | |  |  |
| GO:0005751 | mitochondrial respiratory chain complex IV | 6 | 8 | 0.0065 |
| GO:0030017 | sarcomere | 20 | 47 | 0.0147 |
| GO:0002080 | acrosomal membrane | 3 | 3 | 0.0199 |
| GO:0000275 | mitochondrial proton-transporting ATP synthase complex, catalytic core F(1) | 4 | 5 | 0.0211 |
| GO:0031201 | SNARE complex | 4 | 5 | 0.0211 |
| GO:0005774 | vacuolar membrane | 17 | 40 | 0.0241 |
| GO:0016459 | myosin complex | 7 | 13 | 0.0369 |
| GO:0000922 | spindle pole | 8 | 16 | 0.0424 |

| **Supplementary Table 4:** Impacted pathways in BAT | | | |  | |
| --- | --- | --- | --- | --- | --- |
| **pName** | | | | **pv** | |
| Alzheimer's disease | | | | 0.000999661 | |
| Adrenergic signaling in cardiomyocytes | | | | 0.001756899 | |
| Ascorbate and aldarate metabolism | | | | 0.00213192 | |
| Pertussis | | | | 0.004326391 | |
| Circadian entrainment | | | | 0.007909145 | |
| Alcoholism | | | | 0.008018337 | |
| Chagas disease (American trypanosomiasis) | | | | 0.010055862 | |
| Dopaminergic synapse | | | | 0.012724393 | |
| Biosynthesis of amino acids | | | | 0.014096695 | |
| Glycine, serine and threonine metabolism | | | | 0.01522383 | |
| Glycolysis / Gluconeogenesis | | | | 0.016627608 | |
| Arginine and proline metabolism | | | | 0.01706711 | |
| GABAergic synapse | | | | 0.017074995 | |
| Glycerophospholipid metabolism | | | | 0.018579947 | |
| Mismatch repair | | | | 0.020082392 | |
| Parkinson's disease | | | | 0.021279866 | |
| Sphingolipid signaling pathway | | | | 0.023392627 | |
| Carbon metabolism | | | | 0.023928025 | |
| Systemic lupus erythematosus | | | | 0.024367056 | |
| Oxidative phosphorylation | | | | 0.024454565 | |
| Leukocyte transendothelial migration | | | | 0.025964118 | |
| Gastric acid secretion | | | | 0.031732861 | |
| Staphylococcus aureus infection | | | | 0.032662385 | |
| Retrograde endocannabinoid signaling | | | | 0.040233625 | |
| RIG-I-like receptor signaling pathway | | | | 0.042793343 | |
| Metabolic pathways | | | | 0.044301877 | |
| Cholinergic synapse | | | | 0.045060684 | |
| Chemokine signaling pathway | | | | 0.046066085 | |
| Ras signaling pathway | | | | 0.048576277 | |
| **Supplementary Table 5:** Differentially regulated proteins in IWAT | | | | |  |
| **symbol** | **entrez** | **logfc** | **adjpv** | |  |
| Lrrfip2 | 301035 | -1.62 | 0.000008 | |  |
| RGD1311739 | 311428 | 0.89 | 0.000023 | |  |
| Psmb7 | 85492 | 2.73 | 0.000195 | |  |
| Ndufs6 | 29478 | 2.21 | 0.000496 | |  |
| Ezr | 54319 | -0.79 | 0.000500 | |  |
| Orm1 | 24614 | 1.12 | 0.001359 | |  |
| Bin1 | 117028 | -3.22 | 0.001856 | |  |
| Stk3 | 65189 | -1.52 | 0.001979 | |  |
| Gna11 | 81662 | 0.56 | 0.002852 | |  |
| Sult1a1 | 83783 | 0.87 | 0.003031 | |  |
| Sod3 | 25352 | 0.64 | 0.003225 | |  |
| Atg7 | 312647 | -2.32 | 0.004631 | |  |
| Rpl38 | 689284 | -1.10 | 0.006447 | |  |
| Pip4k2a | 116723 | -1.89 | 0.006578 | |  |
| Tusc5 | 360576 | 0.80 | 0.007389 | |  |
| Usp7 | 360471 | -0.82 | 0.008410 | |  |
| Cd14 | 60350 | 0.94 | 0.008533 | |  |
| Pdcd10 | 494345 | -0.80 | 0.009051 | |  |
| Timm8a1 | 84383 | -1.10 | 0.010498 | |  |
| Egf | 25313 | 3.51 | 0.010740 | |  |
| Rpl29 | 29283 | -0.67 | 0.011947 | |  |
| Marcksl1 | 81520 | -1.64 | 0.012738 | |  |
| Tor1aip1 | 246314 | -1.69 | 0.014289 | |  |
| Vac14 | 307842 | -0.69 | 0.014635 | |  |
| Por | 29441 | 0.39 | 0.015849 | |  |
| Cd74 | 25599 | -3.33 | 0.016453 | |  |
| Plek | 364206 | -1.18 | 0.018541 | |  |
| Tsc22d1 | 498545 | 2.48 | 0.020115 | |  |
| Abcd3 | 25270 | -1.46 | 0.020545 | |  |
| Arhgap17 | 63994 | -1.50 | 0.021408 | |  |
| Xpo1 | 85252 | -0.81 | 0.022951 | |  |
| Dek | 306817 | -2.22 | 0.025033 | |  |
| Scarb1 | 25073 | 0.78 | 0.028908 | |  |
| Bckdhb | 29711 | -2.62 | 0.031066 | |  |
| Lgmn | 63865 | 0.41 | 0.031760 | |  |
| Akr1c14 | 191574 | 0.70 | 0.032774 | |  |
| Rps25 | 122799 | -0.58 | 0.034593 | |  |
| Prps1 | 29562 | -0.53 | 0.036195 | |  |
| Rps26 | 27139 | -0.55 | 0.036434 | |  |
| Kdelr2 | 304290 | -0.69 | 0.038378 | |  |
| Gcs1 | 78947 | 0.32 | 0.046354 | |  |
| Lhpp | 361663 | -3.90 | 0.047177 | |  |
| Lss | 81681 | 1.74 | 0.047278 | |  |
| Prdx6 | 94167 | 0.47 | 0.048182 | |  |
| Vcan | 114122 | 2.79 | 0.050063 | |  |
| Nudcd2 | 287199 | 0.42 | 0.050985 | |  |
| Vamp3 | 29528 | 0.76 | 0.051092 | |  |
| Cox6a1 | 25282 | -3.45 | 0.051458 | |  |
| Bcam | 78958 | 1.64 | 0.051481 | |  |
| LOC100360087 | 100360087 | -0.49 | 0.052279 | |  |
| Magoh | 298385 | -1.20 | 0.052385 | |  |
| Vcl | 305679 | 0.67 | 0.053180 | |  |
| Ptprc | 24699 | -3.37 | 0.053283 | |  |
| Uqcrfs1 | 291103 | -0.28 | 0.054115 | |  |
| Gpx3 | 64317 | 1.02 | 0.055570 | |  |
| Lrpprc | 313867 | -0.50 | 0.056488 | |  |
| Eepd1 | 315500 | 1.32 | 0.056613 | |  |
| Stxbp1 | 25558 | 2.27 | 0.057076 | |  |
| Rab8a | 117103 | -0.80 | 0.057651 | |  |
| Ilf2 | 310612 | -0.86 | 0.057972 | |  |
| LOC100911615 | 100911615 | 0.72 | 0.058561 | |  |
| Trim28 | 116698 | -1.00 | 0.060789 | |  |
| Hnrnpa1 | 29578 | -0.70 | 0.060813 | |  |
| Ppid | 361967 | -1.92 | 0.061759 | |  |
| Necap2 | 298598 | -2.01 | 0.064150 | |  |
| Grpel1 | 79563 | -0.70 | 0.064308 | |  |
| Camk2d | 24246 | -1.11 | 0.065373 | |  |
| Gstm5 | 64352 | 0.80 | 0.068868 | |  |
| Tbca | 366995 | -0.63 | 0.069016 | |  |
| Alb | 24186 | 0.44 | 0.069672 | |  |
| Src | 83805 | 0.55 | 0.069673 | |  |
| Bcat2 | 64203 | -1.14 | 0.071248 | |  |
| Vps29 | 288666 | 0.26 | 0.071307 | |  |
| Cnbp | 64530 | -0.93 | 0.071444 | |  |
| Otc | 25611 | 1.20 | 0.073488 | |  |
| ND4 | 26201 | -0.24 | 0.074632 | |  |
| Timm44 | 29635 | -0.51 | 0.077253 | |  |
| Hba2 | 360504 | 1.08 | 0.078331 | |  |
| Gmfg | 113940 | -2.95 | 0.078606 | |  |
| Raver1 | 298705 | 2.25 | 0.079417 | |  |
| Ctsz | 252929 | -1.11 | 0.079684 | |  |
| Serbp1 | 246303 | 0.77 | 0.079726 | |  |
| Calm3 | 24244 | 0.26 | 0.082538 | |  |
| Ncald | 553106 | -1.36 | 0.084373 | |  |
| Gbp2 | 171164 | -0.64 | 0.086240 | |  |
| Ppt1 | 29411 | -0.47 | 0.086604 | |  |
| Eif4e | 117045 | -0.45 | 0.089913 | |  |
| Gnao1 | 50664 | 1.15 | 0.092194 | |  |
| Syncrip | 363113 | -0.36 | 0.092311 | |  |
| Alpl | 25586 | -2.22 | 0.093379 | |  |
| Rbmxrtl | 307779 | -0.90 | 0.096585 | |  |
| Hspb1 | 24471 | 1.11 | 0.096643 | |  |
| Cct5 | 294864 | -0.43 | 0.097214 | |  |
| Arf5 | 79117 | -0.22 | 0.098562 | |  |
| Andpro | 25030 | 2.70 | 0.099775 | |  |
| Cndp2 | 291394 | 0.35 | 0.102047 | |  |
| Gmps | 295088 | 0.65 | 0.102747 | |  |
| Limd2 | 360646 | -3.93 | 0.103367 | |  |
| Rps28 | 691531 | -0.68 | 0.103369 | |  |
| Dcn | 29139 | 0.56 | 0.104287 | |  |
| C1qa | 298566 | 0.65 | 0.105134 | |  |
| Lbr | 89789 | -2.13 | 0.105335 | |  |
| LOC619574 | 619574 | -2.96 | 0.105834 | |  |
| Prpf19 | 246216 | -1.37 | 0.106789 | |  |
| Cadm3 | 360882 | 1.55 | 0.107698 | |  |
| Pcolce | 29569 | -0.72 | 0.108282 | |  |
| Afdn | 26955 | 0.37 | 0.109297 | |  |
| Cfd | 54249 | 0.92 | 0.109855 | |  |
| Hsd17b11 | 289456 | -0.72 | 0.111035 | |  |
| Stk24 | 361092 | -0.61 | 0.111524 | |  |
| Iah1 | 298917 | -0.54 | 0.111550 | |  |
| Srsf2 | 494445 | -0.63 | 0.112424 | |  |
| Lypla2 | 83510 | -0.70 | 0.113495 | |  |
| Rabggta | 58983 | -0.26 | 0.115613 | |  |
| Dynll1 | 58945 | -0.63 | 0.116414 | |  |
| LOC103690821 | 103690821 | -5.38 | 0.116555 | |  |
| Ddah2 | 294239 | 0.50 | 0.119324 | |  |
| Sptan1 | 64159 | 0.50 | 0.119658 | |  |
| Pycr3 | 300035 | -2.47 | 0.120115 | |  |
| LOC100911248 | 100911248 | -0.53 | 0.120469 | |  |
| Sdha | 157074 | -0.28 | 0.120567 | |  |
| Arpc5 | 360854 | -0.96 | 0.120907 | |  |
| Pcbd1 | 29700 | 2.77 | 0.121663 | |  |
| Sccpdh | 305021 | 0.56 | 0.121973 | |  |
| Gnb1 | 24400 | 0.46 | 0.122525 | |  |
| LOC684988 | 684988 | -0.69 | 0.123734 | |  |
| Lum | 81682 | 0.39 | 0.123888 | |  |
| S100a10 | 81778 | 1.13 | 0.124234 | |  |
| Aox3 | 493909 | 1.53 | 0.124551 | |  |
| Acadm | 24158 | -0.75 | 0.124567 | |  |
| Snap23 | 64630 | 0.42 | 0.124762 | |  |
| Myo1d | 25485 | 1.92 | 0.125068 | |  |
| Anxa2 | 56611 | 0.91 | 0.126984 | |  |
| Sae1 | 308384 | -1.38 | 0.127451 | |  |
| Tuba1c | 300218 | 2.14 | 0.127541 | |  |
| Acsl4 | 113976 | -2.66 | 0.127964 | |  |
| Rpl36 | 58927 | -0.75 | 0.129174 | |  |
| Coro1b | 29474 | -0.91 | 0.129983 | |  |
| Ddx39b | 114612 | -0.76 | 0.130371 | |  |
| Serpina3n | 24795 | 0.93 | 0.133059 | |  |
| Slc9a3r1 | 59114 | -1.43 | 0.133111 | |  |
| Ufc1 | 445268 | 0.96 | 0.133723 | |  |
| Hagh | 24439 | 0.56 | 0.133896 | |  |
| Set | 307947 | -2.75 | 0.134883 | |  |
| Abca2 | 79248 | 0.83 | 0.136241 | |  |
| Rpl10 | 81764 | -0.63 | 0.137193 | |  |
| Epcam | 171577 | -2.83 | 0.139533 | |  |
| Nln | 117041 | -1.75 | 0.140227 | |  |
| Rps21 | 81775 | -0.58 | 0.140256 | |  |
| Arpc5l | 296710 | -0.94 | 0.142448 | |  |
| Dpep1 | 94199 | 0.61 | 0.143621 | |  |
| Cdc42 | 64465 | -0.40 | 0.143971 | |  |
| Hmgb1 | 25459 | -2.74 | 0.145124 | |  |
| Prkcb | 25023 | -2.25 | 0.145733 | |  |
| Sirt2 | 361532 | 0.70 | 0.146533 | |  |
| Gsn | 296654 | 0.84 | 0.149709 | |  |
| Arg1 | 29221 | -3.18 | 0.150180 | |  |
| Arl8b | 500282 | -0.44 | 0.150719 | |  |
| Mybbp1a | 60571 | -2.43 | 0.152501 | |  |
| Oplah | 116684 | -1.84 | 0.152644 | |  |
| Bcat1 | 29592 | 0.70 | 0.154343 | |  |
| Dctn1 | 29167 | 2.23 | 0.155575 | |  |
| Ces1d | 113902 | 0.77 | 0.155957 | |  |
| Mcts1 | 302500 | 1.12 | 0.156172 | |  |
| Dctn4 | 84428 | -0.62 | 0.156189 | |  |
| Rps27l | 681429 | -1.15 | 0.156292 | |  |
| Pecr | 113956 | -1.79 | 0.157226 | |  |
| Tra2b | 117259 | -0.90 | 0.157773 | |  |
| Cpq | 58952 | 0.63 | 0.158239 | |  |
| Vim | 81818 | 0.71 | 0.158932 | |  |
| Coro1a | 155151 | -3.39 | 0.160831 | |  |
| Elavl1 | 363854 | -0.65 | 0.161132 | |  |
| Psmc4 | 117262 | -0.37 | 0.162250 | |  |
| Tubb4b | 296554 | 0.47 | 0.162807 | |  |
| Plvap | 56765 | 0.53 | 0.162951 | |  |
| Tpmt | 690050 | -0.80 | 0.164556 | |  |
| Gna13 | 303634 | -0.51 | 0.165043 | |  |
| Ace | 24310 | 0.92 | 0.165637 | |  |
| Coro7 | 192276 | -2.98 | 0.165684 | |  |
| Aldh9a1 | 64040 | 0.45 | 0.167003 | |  |
| Nid2 | 302248 | 0.76 | 0.167451 | |  |
| Sumo2 | 690244 | -0.74 | 0.167705 | |  |
| Ptbp3 | 83515 | -2.44 | 0.169287 | |  |
| Mvp | 64681 | -0.73 | 0.173324 | |  |
| Pfn1 | 64303 | -1.08 | 0.173499 | |  |
| Serpind1 | 79224 | 0.53 | 0.174777 | |  |
| Hnrnpf | 64200 | -1.02 | 0.176737 | |  |
| Apoc1 | 25292 | 3.77 | 0.177471 | |  |
| Cops2 | 261736 | -3.04 | 0.177771 | |  |
| Cct3 | 295230 | -0.51 | 0.179225 | |  |
| Aldh1a7 | 29651 | 4.10 | 0.179495 | |  |
| Pon1 | 84024 | -1.45 | 0.179907 | |  |
| Atp5j | 94271 | -0.25 | 0.180618 | |  |
| Rpl4 | 64302 | -0.78 | 0.180827 | |  |
| Hnrnpu | 117280 | -0.97 | 0.181579 | |  |
| Ufd1 | 84478 | -0.21 | 0.182866 | |  |
| Scgb1d4 | 293731 | 2.70 | 0.184442 | |  |
| Ndufs2 | 289218 | -0.42 | 0.185120 | |  |
| Calml3 | 307100 | 1.77 | 0.185649 | |  |
| Fabp3 | 79131 | 2.38 | 0.185749 | |  |
| Ppp3r1 | 29748 | -0.99 | 0.185763 | |  |
| Acox3 | 83522 | 0.75 | 0.187330 | |  |
| Cox4i1 | 29445 | -0.36 | 0.187695 | |  |
| Anp32a | 25379 | -1.35 | 0.187706 | |  |
| Plbd1 | 297694 | -1.59 | 0.190050 | |  |
| Kpnb1 | 24917 | -0.30 | 0.191047 | |  |
| Cndp1 | 307212 | 2.81 | 0.192754 | |  |
| Rpl35 | 296709 | -0.75 | 0.194182 | |  |
| Arl3 | 64664 | -0.35 | 0.194833 | |  |
| Psmb10 | 291983 | -1.33 | 0.195615 | |  |
| Tppp3 | 291966 | 0.55 | 0.196539 | |  |
| Ugt1a6 | 113992 | 0.90 | 0.197351 | |  |
| Hnrnpl | 80846 | -0.59 | 0.197806 | |  |
| Atp6v0c | 170667 | -0.42 | 0.197838 | |  |
| Arpc1b | 54227 | -1.25 | 0.197860 | |  |
| Cavin2 | 316384 | 1.21 | 0.198691 | |  |
| Gdi1 | 25183 | 0.33 | 0.199526 | |  |
| Uqcrh | 366448 | 0.29 | 0.201902 | |  |
| Ybx1 | 500538 | -0.83 | 0.202578 | |  |
| Itgb1 | 24511 | 0.59 | 0.203687 | |  |
| Anxa8 | 306283 | 0.82 | 0.203910 | |  |
| Tgfb1i1 | 84574 | 0.53 | 0.205149 | |  |
| Nsfl1c | 83809 | -0.39 | 0.205591 | |  |
| Slc2a4 | 25139 | 0.97 | 0.206215 | |  |
| Psmb9 | 24967 | -0.95 | 0.206320 | |  |
| Fubp1 | 654496 | -1.23 | 0.206714 | |  |
| Cap1 | 64185 | -0.94 | 0.207328 | |  |
| Gimap4 | 286938 | -1.53 | 0.208766 | |  |
| Cavin3 | 85332 | 1.06 | 0.210014 | |  |
| Rps15a | 117053 | -0.88 | 0.210580 | |  |
| Ecm1 | 116662 | 0.96 | 0.213777 | |  |
| Cd44 | 25406 | 3.24 | 0.216345 | |  |
| Tuba4a | 316531 | -0.63 | 0.217005 | |  |
| Khsrp | 171137 | -0.83 | 0.217712 | |  |
| Krt2 | 406228 | 0.20 | 0.221107 | |  |
| Irgm | 303090 | 2.07 | 0.224959 | |  |
| LOC501110 | 501110 | -0.31 | 0.225656 | |  |
| Rpl22 | 81768 | -0.43 | 0.226122 | |  |
| C4a | 24233 | 0.77 | 0.227035 | |  |
| Rgn | 25106 | 1.62 | 0.227052 | |  |
| Cpped1 | 302890 | 0.33 | 0.227974 | |  |
| Prg2 | 58826 | -1.79 | 0.227978 | |  |
| Pecam1 | 29583 | 0.59 | 0.230352 | |  |
| Hsd17b4 | 79244 | 0.69 | 0.230746 | |  |
| Mccc1 | 294972 | -0.21 | 0.230971 | |  |
| Ca5b | 302669 | 1.19 | 0.231316 | |  |
| Sncg | 64347 | 1.19 | 0.232565 | |  |
| Tufm | 293481 | -0.49 | 0.233099 | |  |
| Nap1l1 | 89825 | -0.41 | 0.233641 | |  |
| Plp2 | 302562 | 0.77 | 0.235671 | |  |
| Gphn | 64845 | 3.39 | 0.236104 | |  |
| Cst3 | 25307 | 0.27 | 0.236295 | |  |
| Hacl1 | 85255 | -0.97 | 0.236649 | |  |
| Slc25a5 | 25176 | -0.36 | 0.236974 | |  |
| Trap1 | 287069 | -0.30 | 0.237825 | |  |
| Hnrnpm | 116655 | -0.96 | 0.237910 | |  |
| Myo1c | 65261 | 0.56 | 0.238350 | |  |
| Enpp3 | 54410 | 0.17 | 0.238398 | |  |
| Gyg1 | 81675 | 0.45 | 0.238519 | |  |
| Erp29 | 117030 | -0.28 | 0.239909 | |  |
| Mpc2 | 100359982 | -0.50 | 0.239949 | |  |
| Dnaja1 | 65028 | -1.08 | 0.240466 | |  |
| Pir | 363465 | 0.47 | 0.241062 | |  |
| Qsox1 | 84491 | 1.93 | 0.242226 | |  |
| Psme2 | 29614 | -0.97 | 0.243823 | |  |
| Aimp2 | 288480 | -0.60 | 0.243916 | |  |
| F13a1 | 60327 | 0.45 | 0.244852 | |  |
| Uggt1 | 171129 | -0.53 | 0.245593 | |  |
| Car1 | 310218 | 1.39 | 0.246783 | |  |
| Psma1 | 29668 | -0.46 | 0.247564 | |  |
| Rpl27a | 293418 | -0.71 | 0.248685 | |  |
| Rgs18 | 289076 | 0.38 | 0.249498 | |  |

| **Supplementary Table 6:** GO terms enriched in WAT | | | | |
| --- | --- | --- | --- | --- |
| **goId** | **goName** | **countDE** | **countAll** | **pv_elim** |
| **Biological Process** |  |  |  |  |
| GO:0000381 | regulation of alternative mRNA splicing, via spliceosome | 6 | 12 | 0.0029 |
| GO:0032781 | positive regulation of ATPase activity | 6 | 12 | 0.0029 |
| GO:0032760 | positive regulation of tumor necrosis factor production | 5 | 10 | 0.0066 |
| GO:0042742 | defense response to bacterium | 6 | 14 | 0.0073 |
| GO:0043065 | positive regulation of apoptotic process | 18 | 73 | 0.0074 |
| GO:0000122 | negative regulation of transcription from RNA polymerase II promoter | 11 | 37 | 0.0083 |
| GO:0000077 | DNA damage checkpoint | 3 | 4 | 0.0093 |
| GO:0001960 | negative regulation of cytokine-mediated signaling pathway | 3 | 4 | 0.0093 |
| GO:0002828 | regulation of type 2 immune response | 3 | 4 | 0.0093 |
| GO:0010799 | regulation of peptidyl-threonine phosphorylation | 3 | 4 | 0.0093 |
| GO:0034142 | toll-like receptor 4 signaling pathway | 3 | 4 | 0.0093 |
| GO:0035767 | endothelial cell chemotaxis | 3 | 4 | 0.0093 |
| GO:0044319 | wound healing, spreading of cells | 3 | 4 | 0.0093 |
| GO:0051126 | negative regulation of actin nucleation | 3 | 4 | 0.0093 |
| GO:1990774 | tumor necrosis factor secretion | 3 | 4 | 0.0093 |
| GO:2000648 | positive regulation of stem cell proliferation | 3 | 4 | 0.0093 |
| GO:0033273 | response to vitamin | 10 | 25 | 0.0126 |
| GO:0051384 | response to glucocorticoid | 12 | 45 | 0.0148 |
| GO:0043388 | positive regulation of DNA binding | 4 | 8 | 0.0156 |
| GO:0046717 | acid secretion | 4 | 8 | 0.0156 |
| GO:0032374 | regulation of cholesterol transport | 5 | 12 | 0.0165 |
| GO:0000245 | spliceosomal complex assembly | 3 | 5 | 0.0209 |
| GO:0008209 | androgen metabolic process | 3 | 5 | 0.0209 |
| GO:0032369 | negative regulation of lipid transport | 3 | 5 | 0.0209 |
| GO:0033280 | response to vitamin D | 3 | 5 | 0.0209 |
| GO:0045581 | negative regulation of T cell differentiation | 3 | 5 | 0.0209 |
| GO:0051785 | positive regulation of nuclear division | 3 | 5 | 0.0209 |
| GO:2000279 | negative regulation of DNA biosynthetic process | 3 | 5 | 0.0209 |
| GO:2001020 | regulation of response to DNA damage stimulus | 6 | 17 | 0.021 |
| GO:0007346 | regulation of mitotic cell cycle | 10 | 37 | 0.0232 |
| GO:0034314 | Arp2/3 complex-mediated actin nucleation | 5 | 13 | 0.0239 |
| GO:0061515 | myeloid cell development | 4 | 9 | 0.0251 |
| GO:0051252 | regulation of RNA metabolic process | 44 | 195 | 0.0258 |
| GO:0032845 | negative regulation of homeostatic process | 8 | 28 | 0.0298 |
| GO:0045893 | positive regulation of transcription, DNA-templated | 17 | 78 | 0.031 |
| GO:0001935 | endothelial cell proliferation | 5 | 14 | 0.0331 |
| GO:0015718 | monocarboxylic acid transport | 5 | 14 | 0.0331 |
| GO:0051053 | negative regulation of DNA metabolic process | 6 | 11 | 0.0363 |
| GO:0015758 | glucose transport | 6 | 19 | 0.0364 |
| GO:0010506 | regulation of autophagy | 7 | 24 | 0.0371 |
| GO:0055081 | anion homeostasis | 4 | 10 | 0.0374 |
| GO:0030330 | DNA damage response, signal transduction by p53 class mediator | 3 | 6 | 0.0375 |
| GO:0048026 | positive regulation of mRNA splicing, via spliceosome | 3 | 6 | 0.0375 |
| GO:0048255 | mRNA stabilization | 3 | 6 | 0.0375 |
| GO:0008284 | positive regulation of cell proliferation | 20 | 86 | 0.0429 |
| GO:0030334 | regulation of cell migration | 18 | 87 | 0.0433 |
| GO:0014909 | smooth muscle cell migration | 5 | 15 | 0.0442 |
| GO:0034341 | response to interferon-gamma | 5 | 15 | 0.0442 |
| GO:0071222 | cellular response to lipopolysaccharide | 6 | 20 | 0.0462 |
| GO:0071384 | cellular response to corticosteroid stimulus | 6 | 20 | 0.0462 |
| **Molecular Function** | |  |  |  |
| GO:0070573 | metallodipeptidase activity | 3 | 3 | 0.0026 |
| GO:0019955 | cytokine binding | 4 | 6 | 0.0043 |
| GO:0051015 | actin filament binding | 12 | 40 | 0.0058 |
| GO:0060590 | ATPase regulator activity | 4 | 7 | 0.009 |
| GO:0005080 | protein kinase C binding | 6 | 15 | 0.0112 |
| GO:0003727 | single-stranded RNA binding | 6 | 16 | 0.0159 |
| GO:0004180 | carboxypeptidase activity | 4 | 9 | 0.0258 |
| GO:0036002 | pre-mRNA binding | 4 | 9 | 0.0258 |
| GO:0003697 | single-stranded DNA binding | 5 | 14 | 0.0341 |
| GO:0003725 | double-stranded RNA binding | 6 | 19 | 0.0376 |
| GO:0001530 | lipopolysaccharide binding | 3 | 6 | 0.0383 |
| GO:0008201 | heparin binding | 5 | 15 | 0.0455 |
| GO:0003682 | chromatin binding | 7 | 25 | 0.0475 |
| **Cellular Component** | | |  |  |
| GO:0071013 | catalytic step 2 spliceosome | 7 | 16 | 0.0032 |
| GO:0031528 | microvillus membrane | 4 | 6 | 0.0041 |
| GO:0005811 | lipid droplet | 8 | 24 | 0.0113 |
| GO:0005681 | spliceosomal complex | 10 | 22 | 0.0346 |
| GO:0030315 | T-tubule | 4 | 10 | 0.037 |
| GO:0005885 | Arp2/3 protein complex | 3 | 6 | 0.0372 |
| GO:0016604 | nuclear body | 11 | 46 | 0.0412 |
| GO:0000776 | kinetochore | 5 | 15 | 0.0437 |

| **Supplementary Table 7:** Pathways impacted in IWAT | |
| --- | --- |
| **pName** | **pv** |
| Spliceosome | 0.000141924 |
| Melanoma | 0.016101272 |
| Valine, leucine and isoleucine biosynthesis | 0.019168336 |
| ABC transporters | 0.019168336 |
| Phospholipase D signaling pathway | 0.021481217 |
| Pancreatic cancer | 0.024046645 |
| Cytokine-cytokine receptor interaction | 0.024280456 |
| HIF-1 signaling pathway | 0.027970102 |
| FoxO signaling pathway | 0.030910267 |
| PI3K-Akt signaling pathway | 0.031324199 |
| Fc gamma R-mediated phagocytosis | 0.032654499 |
| Drug metabolism - other enzymes | 0.038234041 |
| Non-small cell lung cancer | 0.04040074 |
| Gap junction | 0.047624149 |

| **Table 8.** List of nodes in protein-protein interaction network in BAT with exercise training | | | |
| --- | --- | --- | --- |
| **Id** | **Label** | **Degree** | **Betweenness** |
| ENSRNOP00000013462 | Rpl4 | 403 | 174197.3 |
| ENSRNOP00000019247 | Rpl27a | 368 | 142415.7 |
| ENSRNOP00000002194 | Rpl24 | 367 | 25541.66 |
| ENSRNOP00000000603 | Rpl10a | 290 | 103764.5 |
| ENSRNOP00000014849 | Rpl29 | 278 | 11315.43 |
| ENSRNOP00000040081 | Gfm1 | 138 | 11143.29 |
| ENSRNOP00000026710 | Gnai3 | 110 | 111426.7 |
| ENSRNOP00000028887 | Pcna | 94 | 116410.5 |
| ENSRNOP00000002533 | Mapk1 | 91 | 290801.3 |
| ENSRNOP00000025303 | Akt2 | 61 | 200959.2 |
| ENSRNOP00000025980 | Hnrnpk | 47 | 385423.2 |
| ENSRNOP00000020647 | Tpi1 | 36 | 47910.3 |
| ENSRNOP00000001911 | Gnb2 | 34 | 21856.2 |
| ENSRNOP00000057188 | Eif3b | 30 | 15939.55 |
| ENSRNOP00000019531 | Tcp1 | 29 | 39505.48 |
| ENSRNOP00000024609 | Uqcrfs1 | 27 | 11648.4 |
| ENSRNOP00000002732 | Atp5o | 25 | 14455.11 |
| ENSRNOP00000002484 | Eif4a2 | 25 | 9881.91 |
| ENSRNOP00000059076 | Dctn1 | 24 | 33858.5 |
| ENSRNOP00000025525 | Cox5a | 23 | 5885.71 |
| ENSRNOP00000003965 | Atp5b | 22 | 12328.27 |
| ENSRNOP00000022487 | Cox5b | 21 | 6761.27 |
| ENSRNOP00000032361 | Eif3d | 19 | 5751.89 |
| ENSRNOP00000007298 | Dlst | 18 | 19106.12 |
| ENSRNOP00000067815 | Atp5e | 18 | 4932.53 |
| ENSRNOP00000002044 | Napa | 17 | 19368 |
| ENSRNOP00000001958 | Mdh2 | 16 | 63676.24 |
| ENSRNOP00000008980 | Dld | 15 | 48405.47 |
| ENSRNOP00000025906 | Ilk | 14 | 25291.5 |
| ENSRNOP00000007558 | Csnk2a1 | 14 | 19795.87 |
| ENSRNOP00000002116 | Atp5j | 14 | 3417.8 |
| ENSRNOP00000024033 | Cox4i1 | 14 | 2422.6 |
| ENSRNOP00000011052 | Cdk5 | 13 | 106253.7 |
| ENSRNOP00000060140 | Acaa2 | 12 | 21722.5 |
| ENSRNOP00000015102 | Idh3a | 11 | 26421.81 |
| ENSRNOP00000046690 | Vps4a | 11 | 19340.5 |
| ENSRNOP00000050322 | Actg2 | 11 | 12135 |
| ENSRNOP00000016432 | Rab7a | 11 | 10926.5 |
| ENSRNOP00000019021 | Cps1 | 10 | 29996.3 |
| ENSRNOP00000043366 | Timeless | 10 | 9716.5 |
| ENSRNOP00000023256 | Slc2a4 | 9 | 77101.2 |
| ENSRNOP00000020322 | Idh1 | 9 | 30158.91 |
| ENSRNOP00000006141 | F11r | 9 | 9716 |
| ENSRNOP00000016520 | Samm50 | 9 | 9716 |
| ENSRNOP00000066894 | Khdrbs1 | 9 | 8497.22 |
| ENSRNOP00000025880 | Dpysl3 | 8 | 157067.4 |
| ENSRNOP00000015956 | Got2 | 8 | 113550.5 |
| ENSRNOP00000023554 | Hmgcs1 | 8 | 6685 |
| ENSRNOP00000017230 | RGD1565317 | 8 | 5039.53 |
| ENSRNOP00000040548 | RGD1563570 | 8 | 3738.91 |
| ENSRNOP00000051318 | LOC100359563 | 8 | 2390.52 |
| ENSRNOP00000023447 | Acly | 7 | 34806.19 |
| ENSRNOP00000005574 | Ndel1 | 7 | 32587.47 |
| ENSRNOP00000027073 | Uba52 | 7 | 31171 |
| ENSRNOP00000021514 | Uqcrc2 | 7 | 13424.48 |
| ENSRNOP00000044696 | Uqcrc1 | 7 | 13424.48 |
| ENSRNOP00000010593 | Sdhb | 7 | 8258.96 |
| ENSRNOP00000001625 | Pfkl | 7 | 7293 |
| ENSRNOP00000012114 | Pdcd6ip | 7 | 6080.5 |
| ENSRNOP00000010383 | LOC100911372 | 7 | 3603.5 |
| ENSRNOP00000026576 | Rps16 | 7 | 3603.5 |
| ENSRNOP00000026528 | Rps5 | 7 | 3542 |
| ENSRNOP00000061250 | Rps11 | 7 | 3491.37 |
| ENSRNOP00000009988 | RGD1560831 | 7 | 896.66 |
| ENSRNOP00000023935 | Rps3 | 7 | 896.66 |
| ENSRNOP00000046737 | Rps15al4 | 7 | 896.66 |
| ENSRNOP00000017239 | Wdr61 | 6 | 6080 |
| ENSRNOP00000063201 | RGD1561102 | 6 | 1680.08 |
| ENSRNOP00000056750 | LOC100364509 | 6 | 850.16 |
| ENSRNOP00000001518 | Rplp0 | 6 | 0.34 |
| ENSRNOP00000005471 | Rpl23 | 6 | 0.34 |
| ENSRNOP00000005511 | | 6 | 0.34 |
| ENSRNOP00000005588 | Rpl26 | 6 | 0.34 |
| ENSRNOP00000009431 | Rpl7 | 6 | 0.34 |
| ENSRNOP00000011314 | Rps20 | 6 | 0.34 |
| ENSRNOP00000021725 | Rpl12 | 6 | 0.34 |
| ENSRNOP00000022348 | Rps23 | 6 | 0.34 |
| ENSRNOP00000024678 | Rps15a | 6 | 0.34 |
| ENSRNOP00000025217 | Rpl17 | 6 | 0.34 |
| ENSRNOP00000030437 | RGD1563956 | 6 | 0.34 |
| ENSRNOP00000032635 | LOC100360449 | 6 | 0.34 |
| ENSRNOP00000034364 | Rpl17 | 6 | 0.34 |
| ENSRNOP00000034767 | RGD1359290 | 6 | 0.34 |
| ENSRNOP00000036391 | Rpl23a | 6 | 0.34 |
| ENSRNOP00000036514 | Rpl5 | 6 | 0.34 |
| ENSRNOP00000037110 | Rpl11 | 6 | 0.34 |
| ENSRNOP00000039111 | | 6 | 0.34 |
| ENSRNOP00000039774 | RGD1560017 | 6 | 0.34 |
| ENSRNOP00000046553 | Rpl8 | 6 | 0.34 |
| ENSRNOP00000066050 | LOC100364116 | 6 | 0.34 |
| ENSRNOP00000065886 | LOC100362684 | 6 | 0.34 |
| ENSRNOP00000064822 | RGD1565048 | 6 | 0.34 |
| ENSRNOP00000047328 | RGD1561333 | 6 | 0.34 |
| ENSRNOP00000066260 | LOC103692519 | 6 | 0.34 |
| ENSRNOP00000042242 | Rps15al4 | 6 | 0.34 |
| ENSRNOP00000045335 | | 6 | 0.34 |
| ENSRNOP00000066866 | LOC103690796 | 6 | 0.34 |
| ENSRNOP00000066750 | LOC100909911 | 6 | 0.34 |
| ENSRNOP00000042929 | LOC688981 | 6 | 0.34 |
| ENSRNOP00000067080 | LOC100360117 | 6 | 0.34 |
| ENSRNOP00000067881 | RGD1564378 | 6 | 0.34 |
| ENSRNOP00000049710 | | 6 | 0.34 |
| ENSRNOP00000067887 | LOC100910721 | 6 | 0.34 |
| ENSRNOP00000066077 | LOC100359951 | 6 | 0.34 |
| ENSRNOP00000064197 | Rpl12 | 6 | 0.34 |
| ENSRNOP00000040966 | Rpl10l | 6 | 0.34 |
| ENSRNOP00000065065 | LOC100910721 | 6 | 0.34 |
| ENSRNOP00000049652 | LOC689899 | 6 | 0.34 |
| ENSRNOP00000041817 | LOC100360449 | 6 | 0.34 |
| ENSRNOP00000043004 | RGD1564606 | 6 | 0.34 |
| ENSRNOP00000067446 | LOC100909911 | 6 | 0.34 |
| ENSRNOP00000065901 | | 6 | 0.34 |
| ENSRNOP00000040232 | | 6 | 0.34 |
| ENSRNOP00000064959 | LOC103692519 | 6 | 0.34 |
| ENSRNOP00000045458 | Rpl26-ps1 | 6 | 0.34 |
| ENSRNOP00000050700 | LOC690335 | 6 | 0.34 |
| ENSRNOP00000057758 | Rpl26-ps2 | 6 | 0.34 |
| ENSRNOP00000060662 | Rpl3 | 6 | 0.34 |
| ENSRNOP00000064566 | LOC100910370 | 6 | 0.34 |
| ENSRNOP00000050533 | RGD1563705 | 6 | 0.34 |
| ENSRNOP00000047749 | LOC680441 | 6 | 0.34 |
| ENSRNOP00000064524 | RGD1563124 | 6 | 0.34 |
| ENSRNOP00000046487 | RGD1562755 | 6 | 0.34 |
| ENSRNOP00000053082 | Rpl5l1 | 6 | 0.34 |
| ENSRNOP00000051016 | LOC100364191 | 6 | 0.34 |
| ENSRNOP00000049286 | Rps15al2 | 6 | 0.34 |
| ENSRNOP00000044111 | RGD1565170 | 6 | 0.34 |
| ENSRNOP00000065423 | | 6 | 0.34 |
| ENSRNOP00000041462 | LOC102555453 | 6 | 0.34 |
| ENSRNOP00000050047 | | 6 | 0.34 |
| ENSRNOP00000047840 | Tp53 | 5 | 98266.48 |
| ENSRNOP00000022892 | Atp5a1 | 5 | 18899.62 |
| ENSRNOP00000049879 | Taf3 | 5 | 18899.62 |
| ENSRNOP00000008477 | Vdac1 | 5 | 14868.26 |
| ENSRNOP00000063666 | Hspd1 | 5 | 3410.54 |
| ENSRNOP00000025446 | Echs1 | 5 | 3040 |
| ENSRNOP00000020670 | Atp5d | 5 | 2925.58 |
| ENSRNOP00000022897 | Rps27 | 5 | 1355.98 |
| ENSRNOP00000048624 | RGD1565415 | 5 | 0.34 |
| ENSRNOP00000055298 | | 5 | 0.34 |
| ENSRNOP00000063004 | | 5 | 0.34 |
| ENSRNOP00000045344 | RGD1559972 | 5 | 0.34 |
| ENSRNOP00000044949 | RGD1560633 | 5 | 0.34 |
| ENSRNOP00000048422 | RGD1562402 | 5 | 0.34 |
| ENSRNOP00000004583 | | 5 | 0.24 |
| ENSRNOP00000005089 | Mrps7 | 5 | 0.24 |
| ENSRNOP00000019508 | Rps2 | 5 | 0.24 |
| ENSRNOP00000019660 | Rpl3l | 5 | 0.24 |
| ENSRNOP00000021803 | Rpl7l1 | 5 | 0.24 |
| ENSRNOP00000036343 | LOC688473 | 5 | 0.24 |
| ENSRNOP00000039003 | | 5 | 0.24 |
| ENSRNOP00000057658 | Rps2-ps6 | 5 | 0.24 |
| ENSRNOP00000046157 | RGD1564469 | 5 | 0.24 |
| ENSRNOP00000045798 | LOC367195 | 5 | 0.24 |
| ENSRNOP00000056260 | Rps14 | 5 | 0.24 |
| ENSRNOP00000044837 | | 5 | 0.24 |
| ENSRNOP00000066548 | | 5 | 0.24 |
| ENSRNOP00000058859 | | 5 | 0.24 |
| ENSRNOP00000066792 | LOC100364509 | 5 | 0.24 |
| ENSRNOP00000047760 | | 5 | 0.24 |
| ENSRNOP00000044563 | LOC680646 | 5 | 0.24 |
| ENSRNOP00000055288 | RGD1562399 | 5 | 0.24 |
| ENSRNOP00000001265 | Rpl21 | 5 | 0 |
| ENSRNOP00000002177 | | 5 | 0 |
| ENSRNOP00000004213 | | 5 | 0 |
| ENSRNOP00000004303 | RGD1559951 | 5 | 0 |
| ENSRNOP00000005872 | Rps27a | 5 | 0 |
| ENSRNOP00000006359 | Rpl19 | 5 | 0 |
| ENSRNOP00000006754 | Rpl7a | 5 | 0 |
| ENSRNOP00000009046 | Rpl34 | 5 | 0 |
| ENSRNOP00000010759 | Rpl15 | 5 | 0 |
| ENSRNOP00000011244 | LOC688684 | 5 | 0 |
| ENSRNOP00000012255 | LOC690096 | 5 | 0 |
| ENSRNOP00000014493 | Rpl32 | 5 | 0 |
| ENSRNOP00000014905 | LOC100360057 | 5 | 0 |
| ENSRNOP00000015408 | RGD1565894 | 5 | 0 |
| ENSRNOP00000015756 | Rpl22l1 | 5 | 0 |
| ENSRNOP00000015893 | | 5 | 0 |
| ENSRNOP00000016329 | Rps3a | 5 | 0 |
| ENSRNOP00000018820 | Rplp1 | 5 | 0 |
| ENSRNOP00000019162 | Rpl35 | 5 | 0 |
| ENSRNOP00000059662 | LOC103693375 | 5 | 0 |
| ENSRNOP00000049014 | Rpl36al | 5 | 0 |
| ENSRNOP00000051203 | | 5 | 0 |
| ENSRNOP00000046409 | | 5 | 0 |
| ENSRNOP00000061874 | | 5 | 0 |
| ENSRNOP00000057262 | LOC690384 | 5 | 0 |
| ENSRNOP00000054703 | Rpl34-ps1 | 5 | 0 |
| ENSRNOP00000034657 | Fau | 5 | 0 |
| ENSRNOP00000037396 | LOC100359986 | 5 | 0 |
| ENSRNOP00000055726 | Rpl28 | 5 | 0 |
| ENSRNOP00000042031 | LOC100360647 | 5 | 0 |
| ENSRNOP00000044605 | LOC100361060 | 5 | 0 |
| ENSRNOP00000039287 | LOC102550668 | 5 | 0 |
| ENSRNOP00000042920 | Rpl22l2 | 5 | 0 |
| ENSRNOP00000048019 | RGD1563145 | 5 | 0 |
| ENSRNOP00000025421 | Rpl18a | 5 | 0 |
| ENSRNOP00000055334 | RGD1565767 | 5 | 0 |
| ENSRNOP00000041435 | | 5 | 0 |
| ENSRNOP00000066592 | | 5 | 0 |
| ENSRNOP00000039099 | Rpl35al1 | 5 | 0 |
| ENSRNOP00000051482 | Rpl31l3 | 5 | 0 |
| ENSRNOP00000041263 | | 5 | 0 |
| ENSRNOP00000041209 | Rpl35al1 | 5 | 0 |
| ENSRNOP00000051427 | RGD1563958 | 5 | 0 |
| ENSRNOP00000054497 | LOC100912027 | 5 | 0 |
| ENSRNOP00000058614 | RGD1564095 | 5 | 0 |
| ENSRNOP00000051332 | | 5 | 0 |
| ENSRNOP00000064082 | LOC100360491 | 5 | 0 |
| ENSRNOP00000047010 | | 5 | 0 |
| ENSRNOP00000045739 | | 5 | 0 |
| ENSRNOP00000067354 | LOC100361079 | 5 | 0 |
| ENSRNOP00000039179 | | 5 | 0 |
| ENSRNOP00000050175 | | 5 | 0 |
| ENSRNOP00000051188 | Rpl35a | 5 | 0 |
| ENSRNOP00000060476 | LOC680579 | 5 | 0 |
| ENSRNOP00000042127 | RGD1566373 | 5 | 0 |
| ENSRNOP00000049635 | LOC102550734 | 5 | 0 |
| ENSRNOP00000063355 | LOC100361259 | 5 | 0 |
| ENSRNOP00000046515 | | 5 | 0 |
| ENSRNOP00000053991 | | 5 | 0 |
| ENSRNOP00000046070 | | 5 | 0 |
| ENSRNOP00000044275 | LOC100910017 | 5 | 0 |
| ENSRNOP00000038214 | LOC100911575 | 5 | 0 |
| ENSRNOP00000047513 | Rpl37a | 5 | 0 |
| ENSRNOP00000048003 | LOC100912182 | 5 | 0 |
| ENSRNOP00000054398 | Rpl30 | 5 | 0 |
| ENSRNOP00000041966 | Rpl21 | 5 | 0 |
| ENSRNOP00000033369 | | 5 | 0 |
| ENSRNOP00000060629 | RGD1561870 | 5 | 0 |
| ENSRNOP00000040611 | Rpl35a | 5 | 0 |
| ENSRNOP00000054699 | RGD1560069 | 5 | 0 |
| ENSRNOP00000048252 | RGD1564617 | 5 | 0 |
| ENSRNOP00000041774 | RGD1564730 | 5 | 0 |
| ENSRNOP00000065371 | RGD1564617 | 5 | 0 |
| ENSRNOP00000064270 | RGD1561137 | 5 | 0 |
| ENSRNOP00000049416 | RGD1563835 | 5 | 0 |
| ENSRNOP00000046600 | LOC306079 | 5 | 0 |
| ENSRNOP00000067572 | Rps6 | 5 | 0 |
| ENSRNOP00000046301 | RGD1564839 | 5 | 0 |
| ENSRNOP00000040955 | LOC100361079 | 5 | 0 |
| ENSRNOP00000048495 | | 5 | 0 |
| ENSRNOP00000054048 | LOC100912182 | 5 | 0 |
| ENSRNOP00000048999 | Rpl31l4 | 5 | 0 |
| ENSRNOP00000064745 | | 5 | 0 |
| ENSRNOP00000020635 | LOC100359922 | 5 | 0 |
| ENSRNOP00000065066 | LOC100365839 | 5 | 0 |
| ENSRNOP00000047511 | | 5 | 0 |
| ENSRNOP00000053986 | RGD1565183 | 5 | 0 |
| ENSRNOP00000046281 | LOC686074 | 5 | 0 |
| ENSRNOP00000051114 | | 5 | 0 |
| ENSRNOP00000049709 | | 5 | 0 |
| ENSRNOP00000041625 | LOC100362751 | 5 | 0 |
| ENSRNOP00000042633 | | 5 | 0 |
| ENSRNOP00000050328 | RGD1562055 | 5 | 0 |
| ENSRNOP00000043092 | RGD1561317 | 5 | 0 |
| ENSRNOP00000038065 | Rpl6 | 5 | 0 |
| ENSRNOP00000051743 | | 5 | 0 |
| ENSRNOP00000047759 | | 5 | 0 |
| ENSRNOP00000051312 | Rpl21 | 5 | 0 |
| ENSRNOP00000041191 | | 5 | 0 |
| ENSRNOP00000040073 | LOC103691563 | 5 | 0 |
| ENSRNOP00000051134 | | 5 | 0 |
| ENSRNOP00000049054 | Rpl35a | 5 | 0 |
| ENSRNOP00000055671 | | 5 | 0 |
| ENSRNOP00000022184 | Rps12 | 5 | 0 |
| ENSRNOP00000059772 | RGD1565566 | 5 | 0 |
| ENSRNOP00000049666 | | 5 | 0 |
| ENSRNOP00000028481 | Fau | 5 | 0 |
| ENSRNOP00000053160 | | 5 | 0 |
| ENSRNOP00000042277 | | 5 | 0 |
| ENSRNOP00000046578 | | 5 | 0 |
| ENSRNOP00000058934 | Rpl36 | 5 | 0 |
| ENSRNOP00000041199 | | 5 | 0 |
| ENSRNOP00000046953 | LOC102554602 | 5 | 0 |
| ENSRNOP00000042068 | | 5 | 0 |
| ENSRNOP00000048808 | Rpl21 | 5 | 0 |
| ENSRNOP00000028555 | Rpl18 | 5 | 0 |
| ENSRNOP00000065877 | LOC102548369 | 5 | 0 |
| ENSRNOP00000054740 | LOC498555 | 5 | 0 |
| ENSRNOP00000041530 | | 5 | 0 |
| ENSRNOP00000042454 | | 5 | 0 |
| ENSRNOP00000049831 | | 5 | 0 |
| ENSRNOP00000031078 | Rpl31 | 5 | 0 |
| ENSRNOP00000067596 | Rpl30l1 | 5 | 0 |
| ENSRNOP00000053863 | LOC100911575 | 5 | 0 |
| ENSRNOP00000066420 | LOC100361143 | 5 | 0 |
| ENSRNOP00000051135 | Rpl6-ps1 | 5 | 0 |
| ENSRNOP00000066016 | LOC100912027 | 5 | 0 |
| ENSRNOP00000067411 | | 5 | 0 |
| ENSRNOP00000046669 | | 5 | 0 |
| ENSRNOP00000055393 | | 5 | 0 |
| ENSRNOP00000041638 | Rpl32 | 5 | 0 |
| ENSRNOP00000042567 | | 5 | 0 |
| ENSRNOP00000045849 | RGD1561195 | 5 | 0 |
| ENSRNOP00000039786 | | 5 | 0 |
| ENSRNOP00000045195 | LOC100360439 | 5 | 0 |
| ENSRNOP00000061747 | Rpl14 | 5 | 0 |
| ENSRNOP00000027976 | Rpl13a | 5 | 0 |
| ENSRNOP00000021161 | | 5 | 0 |
| ENSRNOP00000028060 | Rpl27 | 5 | 0 |
| ENSRNOP00000048311 | RGD1563157 | 5 | 0 |
| ENSRNOP00000042560 | | 5 | 0 |
| ENSRNOP00000052049 | Eftud2 | 4 | 352858 |
| ENSRNOP00000000733 | Fyn | 4 | 165535.3 |
| ENSRNOP00000027999 | Acadsb | 4 | 25159 |
| ENSRNOP00000019059 | Idh2 | 4 | 14033.68 |
| ENSRNOP00000044732 | Prkaca | 4 | 13570.3 |
| ENSRNOP00000009681 | Idh3B | 4 | 10942.57 |
| ENSRNOP00000027911 | Lipe | 4 | 2449.78 |
| ENSRNOP00000060568 | Rps28 | 4 | 1452.04 |
| ENSRNOP00000018227 | Pgam2 | 4 | 1178 |
| ENSRNOP00000035156 | Rps15 | 4 | 327.19 |
| ENSRNOP00000062631 | Rps15-ps2 | 4 | 327.19 |
| ENSRNOP00000008337 | Aco1 | 4 | 16.83 |
| ENSRNOP00000015278 | LOC680700 | 4 | 0.24 |
| ENSRNOP00000036943 | | 4 | 0.24 |
| ENSRNOP00000039797 | LOC103690888 | 4 | 0.24 |
| ENSRNOP00000047911 | RGD1563352 | 4 | 0.24 |
| ENSRNOP00000052873 | LOC100911847 | 4 | 0.24 |
| ENSRNOP00000004278 | Rps4x | 4 | 0 |
| ENSRNOP00000004836 | Atp5h | 4 | 0 |
| ENSRNOP00000005815 | Mrpl13 | 4 | 0 |
| ENSRNOP00000006662 | RGD1566369 | 4 | 0 |
| ENSRNOP00000007683 | | 4 | 0 |
| ENSRNOP00000011333 | Rps7 | 4 | 0 |
| ENSRNOP00000013868 | LOC100361180 | 4 | 0 |
| ENSRNOP00000017067 | Cyc1 | 4 | 0 |
| ENSRNOP00000017280 | Mrpl3 | 4 | 0 |
| ENSRNOP00000020451 | Mrps5 | 4 | 0 |
| ENSRNOP00000021920 | LOC100911417 | 4 | 0 |
| ENSRNOP00000023456 | Imp3 | 4 | 0 |
| ENSRNOP00000024326 | Mrto4 | 4 | 0 |
| ENSRNOP00000024380 | Mrpl2 | 4 | 0 |
| ENSRNOP00000027029 | Mrps12 | 4 | 0 |
| ENSRNOP00000027091 | mrpl11 | 4 | 0 |
| ENSRNOP00000028517 | Mrpl16 | 4 | 0 |
| ENSRNOP00000029336 | Mrpl22 | 4 | 0 |
| ENSRNOP00000034042 | mrpl24 | 4 | 0 |
| ENSRNOP00000040969 | Atp5l | 4 | 0 |
| ENSRNOP00000048498 | LOC500350 | 4 | 0 |
| ENSRNOP00000065147 | Atp5f1 | 4 | 0 |
| ENSRNOP00000067503 | LOC691427 | 4 | 0 |
| ENSRNOP00000045007 | Mrpl17 | 4 | 0 |
| ENSRNOP00000043988 | Rps4x | 4 | 0 |
| ENSRNOP00000033162 | LOC500594 | 4 | 0 |
| ENSRNOP00000036690 | Rps13 | 4 | 0 |
| ENSRNOP00000046067 | Mrps10 | 4 | 0 |
| ENSRNOP00000033144 | Rps25 | 4 | 0 |
| ENSRNOP00000067129 | Rps27l | 4 | 0 |
| ENSRNOP00000051848 | Mrpl12 | 4 | 0 |
| ENSRNOP00000021625 | Npsr1 | 4 | 0 |
| ENSRNOP00000065173 | | 4 | 0 |
| ENSRNOP00000066863 | RGD1560821 | 4 | 0 |
| ENSRNOP00000049546 | Rps4y2 | 4 | 0 |
| ENSRNOP00000025224 | Rpsa | 4 | 0 |
| ENSRNOP00000066808 | LOC100910336 | 4 | 0 |
| ENSRNOP00000067470 | LOC683961 | 4 | 0 |
| ENSRNOP00000065487 | LOC100363452 | 4 | 0 |
| ENSRNOP00000042941 | LOC500148 | 4 | 0 |
| ENSRNOP00000042092 | Rsl1d1 | 4 | 0 |
| ENSRNOP00000041853 | LOC297756 | 4 | 0 |
| ENSRNOP00000066362 | LOC100362987 | 4 | 0 |
| ENSRNOP00000049713 | LOC103693375 | 4 | 0 |
| ENSRNOP00000045098 | LOC103690821 | 4 | 0 |
| ENSRNOP00000050353 | LOC682793 | 4 | 0 |
| ENSRNOP00000055790 | LOC103692831 | 4 | 0 |
| ENSRNOP00000042286 | LOC680512 | 4 | 0 |
| ENSRNOP00000041329 | LOC103694404 | 4 | 0 |
| ENSRNOP00000044553 | LOC103694404 | 4 | 0 |
| ENSRNOP00000027226 | LOC100360573 | 4 | 0 |
| ENSRNOP00000049665 | LOC103692785 | 4 | 0 |
| ENSRNOP00000047281 | Rps27a-ps6 | 4 | 0 |
| ENSRNOP00000063142 | | 4 | 0 |
| ENSRNOP00000061370 | | 4 | 0 |
| ENSRNOP00000044116 | RGD1561310 | 4 | 0 |
| ENSRNOP00000050202 | Rpl38 | 4 | 0 |
| ENSRNOP00000066511 | | 4 | 0 |
| ENSRNOP00000056331 | Rpl37-ps1 | 4 | 0 |
| ENSRNOP00000045213 | RGD1561636 | 4 | 0 |
| ENSRNOP00000048664 | Rps27a | 4 | 0 |
| ENSRNOP00000042288 | LOC685963 | 4 | 0 |
| ENSRNOP00000065827 | | 4 | 0 |
| ENSRNOP00000049028 | LOC680353 | 4 | 0 |
| ENSRNOP00000025888 | Rps17 | 4 | 0 |
| ENSRNOP00000039155 | Rpl37 | 4 | 0 |
| ENSRNOP00000048903 | LOC100363469 | 4 | 0 |
| ENSRNOP00000064320 | Rps17 | 4 | 0 |
| ENSRNOP00000054474 | LOC100360654 | 4 | 0 |
| ENSRNOP00000042022 | LOC685963 | 4 | 0 |
| ENSRNOP00000023368 | Rpl38 | 4 | 0 |
| ENSRNOP00000067793 | | 4 | 0 |
| ENSRNOP00000045912 | | 4 | 0 |
| ENSRNOP00000041920 | RGD1559955 | 4 | 0 |
| ENSRNOP00000041458 | RGD1562381 | 4 | 0 |
| ENSRNOP00000031121 | LOC100362366 | 4 | 0 |
| ENSRNOP00000062764 | | 4 | 0 |
| ENSRNOP00000059382 | Rps24 | 4 | 0 |
| ENSRNOP00000049205 | Rpl37 | 4 | 0 |
| ENSRNOP00000045390 | RGD1562265 | 4 | 0 |
| ENSRNOP00000064461 | | 4 | 0 |
| ENSRNOP00000040295 | Rpl39l | 4 | 0 |
| ENSRNOP00000048620 | RGD1561453 | 4 | 0 |
| ENSRNOP00000058757 | | 4 | 0 |
| ENSRNOP00000027086 | Cnbd2 | 4 | 0 |
| ENSRNOP00000044063 | LOC686066 | 4 | 0 |
| ENSRNOP00000065074 | Uqcrq | 4 | 0 |
| ENSRNOP00000046414 | Mt-co2 | 4 | 0 |
| ENSRNOP00000048723 | Cox6b1 | 4 | 0 |
| ENSRNOP00000026908 | Cox6a2 | 4 | 0 |
| ENSRNOP00000030997 | Cox6b1 | 4 | 0 |
| ENSRNOP00000049222 | Cox7c | 4 | 0 |
| ENSRNOP00000012739 | Src | 3 | 217843 |
| ENSRNOP00000030030 | Cad | 3 | 114054.3 |
| ENSRNOP00000003867 | Gsk3b | 3 | 77508.73 |
| ENSRNOP00000007621 | Ppp2ca | 3 | 41511.36 |
| ENSRNOP00000040878 | Gapdh | 3 | 26447.65 |
| ENSRNOP00000001292 | Aacs | 3 | 23538.18 |
| ENSRNOP00000022862 | Ppp2r5d | 3 | 20403.85 |
| ENSRNOP00000013249 | Bckdhb | 3 | 17389.33 |
| ENSRNOP00000043928 | Pxn | 3 | 13288 |
| ENSRNOP00000026920 | Hsp90ab1 | 3 | 6046.07 |
| ENSRNOP00000062228 | Prkacb | 3 | 1206.5 |
| ENSRNOP00000038369 | Akt1 | 3 | 737 |
| ENSRNOP00000034921 | Cs | 3 | 29.22 |
| ENSRNOP00000010573 | Acat1 | 3 | 6 |
| ENSRNOP00000005786 | LOC100911110 | 3 | 1.22 |
| ENSRNOP00000063484 | Eif3a | 3 | 1.22 |
| ENSRNOP00000000072 | Atp5i | 3 | 0 |
| ENSRNOP00000000617 | Mapk14 | 3 | 0 |
| ENSRNOP00000000700 | | 3 | 0 |
| ENSRNOP00000001545 | Cox6a1 | 3 | 0 |
| ENSRNOP00000002834 | Mrpl1 | 3 | 0 |
| ENSRNOP00000004114 | LOC690271 | 3 | 0 |
| ENSRNOP00000007194 | | 3 | 0 |
| ENSRNOP00000009325 | Mapk11 | 3 | 0 |
| ENSRNOP00000009815 | Atp5g1 | 3 | 0 |
| ENSRNOP00000010418 | Cox4i2 | 3 | 0 |
| ENSRNOP00000011619 | Mrpl15 | 3 | 0 |
| ENSRNOP00000013548 | Mrps2 | 3 | 0 |
| ENSRNOP00000014058 | LOC100363502 | 3 | 0 |
| ENSRNOP00000014407 | Cox6c | 3 | 0 |
| ENSRNOP00000017602 | LOC100359763 | 3 | 0 |
| ENSRNOP00000017738 | | 3 | 0 |
| ENSRNOP00000020675 | Atp5g2 | 3 | 0 |
| ENSRNOP00000025007 | Mrps11 | 3 | 0 |
| ENSRNOP00000026373 | Gnao1 | 3 | 0 |
| ENSRNOP00000029426 | Atp5j2 | 3 | 0 |
| ENSRNOP00000036682 | LOC100359687 | 3 | 0 |
| ENSRNOP00000049769 | Mt-atp6 | 3 | 0 |
| ENSRNOP00000055032 | Atp5g3 | 3 | 0 |
| ENSRNOP00000042935 | Rps21-ps1 | 3 | 0 |
| ENSRNOP00000049519 | Efl1 | 3 | 0 |
| ENSRNOP00000021899 | Mrps9 | 3 | 0 |
| ENSRNOP00000045893 | Mrpl4 | 3 | 0 |
| ENSRNOP00000056140 | | 3 | 0 |
| ENSRNOP00000043087 | Gfm2 | 3 | 0 |
| ENSRNOP00000041821 | Eef2 | 3 | 0 |
| ENSRNOP00000064782 | | 3 | 0 |
| ENSRNOP00000044874 | RGD1559877 | 3 | 0 |
| ENSRNOP00000042164 | LOC100359671 | 3 | 0 |
| ENSRNOP00000065999 | LOC100911337 | 3 | 0 |
| ENSRNOP00000065281 | LOC100911337 | 3 | 0 |
| ENSRNOP00000048847 | | 3 | 0 |
| ENSRNOP00000061442 | LOC100362339 | 3 | 0 |
| ENSRNOP00000045516 | Wdr31 | 3 | 0 |
| ENSRNOP00000042902 | LOC100360843 | 3 | 0 |
| ENSRNOP00000063451 | RGD1559724 | 3 | 0 |
| ENSRNOP00000048979 | Rps27a-ps5 | 3 | 0 |
| ENSRNOP00000030289 | | 3 | 0 |
| ENSRNOP00000041612 | | 3 | 0 |
| ENSRNOP00000061911 | | 3 | 0 |
| ENSRNOP00000044806 | RGD1565117 | 3 | 0 |
| ENSRNOP00000027780 | Rps27a-ps12 | 3 | 0 |
| ENSRNOP00000064853 | LOC100911371 | 3 | 0 |
| ENSRNOP00000040306 | RGD1563613 | 3 | 0 |
| ENSRNOP00000047999 | | 3 | 0 |
| ENSRNOP00000056689 | | 3 | 0 |
| ENSRNOP00000039845 | Rps19l1 | 3 | 0 |
| ENSRNOP00000047391 | LOC100912210 | 3 | 0 |
| ENSRNOP00000065157 | | 3 | 0 |
| ENSRNOP00000051317 | | 3 | 0 |
| ENSRNOP00000048658 | LOC102554992 | 3 | 0 |
| ENSRNOP00000041744 | RGD1564138 | 3 | 0 |
| ENSRNOP00000048116 | LOC100361854 | 3 | 0 |
| ENSRNOP00000044301 | LOC688899 | 3 | 0 |
| ENSRNOP00000043543 | LOC103690015 | 3 | 0 |
| ENSRNOP00000031049 | RGD1563300 | 3 | 0 |
| ENSRNOP00000027246 | Rps19 | 3 | 0 |
| ENSRNOP00000064904 | | 3 | 0 |
| ENSRNOP00000067306 | | 3 | 0 |
| ENSRNOP00000048289 | | 3 | 0 |
| ENSRNOP00000027054 | Ogdhl | 3 | 0 |
| ENSRNOP00000054026 | Ogdh | 3 | 0 |
| ENSRNOP00000050941 | RGD1564325 | 3 | 0 |
| ENSRNOP00000033706 | Uqcrb | 3 | 0 |
| ENSRNOP00000048883 | Uqcrb | 3 | 0 |
| ENSRNOP00000039048 | Mt-co1 | 3 | 0 |
| ENSRNOP00000039818 | RGD1562758 | 2 | 24087.5 |
| ENSRNOP00000026224 | Stx4 | 2 | 20434 |
| ENSRNOP00000043542 | Bcl2l1 | 2 | 15861.62 |
| ENSRNOP00000000628 | Cdkn1a | 2 | 12658.67 |
| ENSRNOP00000050173 | Cdkn1b | 2 | 12658.67 |
| ENSRNOP00000060736 | Acacb | 2 | 12194.92 |
| ENSRNOP00000049438 | Acaca | 2 | 12194.92 |
| ENSRNOP00000000157 | Pik3r3 | 2 | 11603.06 |
| ENSRNOP00000025687 | Pik3r1 | 2 | 11603.06 |
| ENSRNOP00000038073 | Hadha | 2 | 11390.5 |
| ENSRNOP00000066388 | LOC100911186 | 2 | 11390.5 |
| ENSRNOP00000025281 | Tomm40 | 2 | 10890 |
| ENSRNOP00000003768 | Bcl2 | 2 | 9947.43 |
| ENSRNOP00000013831 | Raf1 | 2 | 9947.43 |
| ENSRNOP00000016221 | Tsc2 | 2 | 9947.43 |
| ENSRNOP00000016885 | Eif4ebp1 | 2 | 9947.43 |
| ENSRNOP00000018326 | Creb1 | 2 | 9947.43 |
| ENSRNOP00000061683 | Pdpk1 | 2 | 9947.43 |
| ENSRNOP00000020267 | Dbt | 2 | 8922.67 |
| ENSRNOP00000003696 | Pafah1b1 | 2 | 8899.91 |
| ENSRNOP00000008120 | Dctn2 | 2 | 8899.91 |
| ENSRNOP00000054753 | Disc1 | 2 | 8899.91 |
| ENSRNOP00000029515 | Gpi | 2 | 8484 |
| ENSRNOP00000020663 | Ppp2cb | 2 | 7796.79 |
| ENSRNOP00000023741 | Ctr9 | 2 | 7278 |
| ENSRNOP00000022779 | Actr1b | 2 | 6040 |
| ENSRNOP00000026792 | Actr1a | 2 | 6040 |
| ENSRNOP00000011226 | Chek1 | 2 | 5440.5 |
| ENSRNOP00000022231 | Mcm2 | 2 | 5440.5 |
| ENSRNOP00000013720 | Chmp4c | 2 | 3636 |
| ENSRNOP00000045686 | Chmp4bl1 | 2 | 3636 |
| ENSRNOP00000026470 | Jund | 2 | 3537.56 |
| ENSRNOP00000011732 | Jun | 2 | 3537.56 |
| ENSRNOP00000007100 | Ywhae | 2 | 3297.28 |
| ENSRNOP00000004797 | Tuba4a | 2 | 2639.59 |
| ENSRNOP00000013863 | Tubb4b | 2 | 2639.59 |
| ENSRNOP00000065633 | Tubb4a | 2 | 2639.59 |
| ENSRNOP00000009556 | LOC103692716 | 2 | 1931.55 |
| ENSRNOP00000001415 | Elavl1 | 2 | 1218 |
| ENSRNOP00000052160 | Hnrnpa1 | 2 | 1208.67 |
| ENSRNOP00000027057 | Xrcc1 | 2 | 965.17 |
| ENSRNOP00000028328 | Bax | 2 | 165.64 |
| ENSRNOP00000022309 | Got1 | 2 | 53.48 |
| ENSRNOP00000018177 | Pgk2 | 2 | 9.95 |
| ENSRNOP00000065201 | | 2 | 9.95 |
| ENSRNOP00000024106 | Eno1 | 2 | 9.95 |
| ENSRNOP00000014637 | Hadhb | 2 | 6 |
| ENSRNOP00000000621 | Mapk13 | 2 | 0 |
| ENSRNOP00000005389 | Adcy3 | 2 | 0 |
| ENSRNOP00000006789 | Adcy8 | 2 | 0 |
| ENSRNOP00000008011 | Gngt2 | 2 | 0 |
| ENSRNOP00000011429 | Mdh1 | 2 | 0 |
| ENSRNOP00000012274 | Dpysl5 | 2 | 0 |
| ENSRNOP00000012996 | Dpysl2 | 2 | 0 |
| ENSRNOP00000013375 | Eif2s1 | 2 | 0 |
| ENSRNOP00000013695 | Eif5 | 2 | 0 |
| ENSRNOP00000014219 | Gngt1 | 2 | 0 |
| ENSRNOP00000014817 | Eif3l | 2 | 0 |
| ENSRNOP00000014871 | Gnb4 | 2 | 0 |
| ENSRNOP00000017017 | Pmpcb | 2 | 0 |
| ENSRNOP00000017409 | Eif3m | 2 | 0 |
| ENSRNOP00000017718 | LOC100912445 | 2 | 0 |
| ENSRNOP00000018336 | Sdha | 2 | 0 |
| ENSRNOP00000020700 | Adcy7 | 2 | 0 |
| ENSRNOP00000021017 | Eif3f | 2 | 0 |
| ENSRNOP00000021480 | Gnb3 | 2 | 0 |
| ENSRNOP00000021671 | Parva | 2 | 0 |
| ENSRNOP00000022437 | Eif3j | 2 | 0 |
| ENSRNOP00000025782 | Eif3c | 2 | 0 |
| ENSRNOP00000027986 | Eif3g | 2 | 0 |
| ENSRNOP00000029790 | Eif3el1 | 2 | 0 |
| ENSRNOP00000030371 | Rps18 | 2 | 0 |
| ENSRNOP00000040056 | RGD1561919 | 2 | 0 |
| ENSRNOP00000043254 | Eif4h | 2 | 0 |
| ENSRNOP00000049629 | Eif4g1 | 2 | 0 |
| ENSRNOP00000038994 | Adcy2 | 2 | 0 |
| ENSRNOP00000046455 | Mapk12 | 2 | 0 |
| ENSRNOP00000066965 | LOC100912034 | 2 | 0 |
| ENSRNOP00000022550 | Gnai2 | 2 | 0 |
| ENSRNOP00000046488 | Adcy5 | 2 | 0 |
| ENSRNOP00000016417 | Prkcb | 2 | 0 |
| ENSRNOP00000065598 | Gng4 | 2 | 0 |
| ENSRNOP00000004699 | Prkca | 2 | 0 |
| ENSRNOP00000027719 | Adcy4 | 2 | 0 |
| ENSRNOP00000065327 | | 2 | 0 |
| ENSRNOP00000047355 | | 2 | 0 |
| ENSRNOP00000049626 | | 2 | 0 |
| ENSRNOP00000039429 | | 2 | 0 |
| ENSRNOP00000047767 | | 2 | 0 |
| ENSRNOP00000063940 | | 2 | 0 |
| ENSRNOP00000045954 | | 2 | 0 |
| ENSRNOP00000063128 | Rpl7a | 2 | 0 |
| ENSRNOP00000049619 | | 2 | 0 |
| ENSRNOP00000046036 | | 2 | 0 |
| ENSRNOP00000046427 | | 2 | 0 |
| ENSRNOP00000042800 | | 2 | 0 |
| ENSRNOP00000045940 | | 2 | 0 |
| ENSRNOP00000048713 | | 2 | 0 |
| ENSRNOP00000036381 | Nras | 2 | 0 |
| ENSRNOP00000044340 | Gnb1 | 2 | 0 |
| ENSRNOP00000065820 | | 2 | 0 |
| ENSRNOP00000008736 | Atp6v0c | 2 | 0 |
| ENSRNOP00000026704 | Atp6v0b | 2 | 0 |
| ENSRNOP00000048392 | RGD1559629 | 2 | 0 |
| ENSRNOP00000060229 | Sucla2 | 2 | 0 |
| ENSRNOP00000059952 | Dhtkd1 | 2 | 0 |
| ENSRNOP00000032890 | Dlat | 2 | 0 |
| ENSRNOP00000010545 | Pdhb | 2 | 0 |
| ENSRNOP00000067239 | | 2 | 0 |
| ENSRNOP00000064678 | LOC100912024 | 2 | 0 |
| ENSRNOP00000047371 | Rps18l1 | 2 | 0 |
| ENSRNOP00000040282 | RGD1565912 | 2 | 0 |
| ENSRNOP00000046090 | LOC100362298 | 2 | 0 |
| ENSRNOP00000063671 | Crmp1 | 2 | 0 |
| ENSRNOP00000035591 | Cox6b2 | 2 | 0 |
| ENSRNOP00000051326 | Mt-cox3 | 2 | 0 |
| ENSRNOP00000061674 | Pik3cd | 2 | 0 |
| ENSRNOP00000035786 | LOC100910021 | 2 | 0 |
| ENSRNOP00000061066 | Eif3k | 2 | 0 |
| ENSRNOP00000067288 | | 2 | 0 |
| ENSRNOP00000000078 | Ppp2r5a | 1 | 0 |
| ENSRNOP00000000102 | LOC103691556 | 1 | 0 |
| ENSRNOP00000000174 | Chm | 1 | 0 |
| ENSRNOP00000000249 | Grm6 | 1 | 0 |
| ENSRNOP00000000327 | Foxo3 | 1 | 0 |
| ENSRNOP00000000553 | Pfdn6 | 1 | 0 |
| ENSRNOP00000000576 | Bak1 | 1 | 0 |
| ENSRNOP00000000627 | Srsf3 | 1 | 0 |
| ENSRNOP00000000783 | Cdk1 | 1 | 0 |
| ENSRNOP00000000904 | Chmp2b | 1 | 0 |
| ENSRNOP00000000907 | Htr1f | 1 | 0 |
| ENSRNOP00000001054 | Gja1 | 1 | 0 |
| ENSRNOP00000001123 | Csnk2b | 1 | 0 |
| ENSRNOP00000001154 | Rbmx | 1 | 0 |
| ENSRNOP00000001227 | Cct6a | 1 | 0 |
| ENSRNOP00000001417 | Rac1 | 1 | 0 |
| ENSRNOP00000001444 | Rfc3 | 1 | 0 |
| ENSRNOP00000001498 | Rfc5 | 1 | 0 |
| ENSRNOP00000001539 | Srsf9 | 1 | 0 |
| ENSRNOP00000001556 | Acads | 1 | 0 |
| ENSRNOP00000001605 | Pwp2 | 1 | 0 |
| ENSRNOP00000001654 | Gna12 | 1 | 0 |
| ENSRNOP00000001685 | Clip1 | 1 | 0 |
| ENSRNOP00000001779 | Gnaz | 1 | 0 |
| ENSRNOP00000001825 | Mcm7 | 1 | 0 |
| ENSRNOP00000001989 | Rfc2 | 1 | 0 |
| ENSRNOP00000002053 | Snrpa | 1 | 0 |
| ENSRNOP00000002064 | RGD1559534 | 1 | 0 |
| ENSRNOP00000002072 | Pdk1 | 1 | 0 |
| ENSRNOP00000002134 | Mtx2 | 1 | 0 |
| ENSRNOP00000002169 | Cct8 | 1 | 0 |
| ENSRNOP00000002174 | Atf2 | 1 | 0 |
| ENSRNOP00000002247 | Ets2 | 1 | 0 |
| ENSRNOP00000002299 | Chaf1b | 1 | 0 |
| ENSRNOP00000002323 | Dvl3 | 1 | 0 |
| ENSRNOP00000002410 | Ehhadh | 1 | 0 |
| ENSRNOP00000002487 | Rfc4 | 1 | 0 |
| ENSRNOP00000002510 | Mcm4 | 1 | 0 |
| ENSRNOP00000002907 | Aasdh | 1 | 0 |
| ENSRNOP00000003092 | Casr | 1 | 0 |
| ENSRNOP00000003188 | RGD1566265 | 1 | 0 |
| ENSRNOP00000003460 | Mrps14 | 1 | 0 |
| ENSRNOP00000003715 | Vps4b | 1 | 0 |
| ENSRNOP00000003735 | Sstr2 | 1 | 0 |
| ENSRNOP00000003840 | Phlpp1 | 1 | 0 |
| ENSRNOP00000003907 | Rfc1 | 1 | 0 |
| ENSRNOP00000003954 | Ccnb3 | 1 | 0 |
| ENSRNOP00000004228 | Sdhc | 1 | 0 |
| ENSRNOP00000004229 | Rpa1 | 1 | 0 |
| ENSRNOP00000004232 | Parp1 | 1 | 0 |
| ENSRNOP00000004406 | Cxcr3 | 1 | 0 |
| ENSRNOP00000004495 | Cdc73 | 1 | 0 |
| ENSRNOP00000004499 | Mcm9 | 1 | 0 |
| ENSRNOP00000004570 | Gcdh | 1 | 0 |
| ENSRNOP00000004602 | Adora1 | 1 | 0 |
| ENSRNOP00000004947 | Mrpl27 | 1 | 0 |
| ENSRNOP00000004969 | Mcm6 | 1 | 0 |
| ENSRNOP00000005039 | Rilp | 1 | 0 |
| ENSRNOP00000005076 | Rgs9 | 1 | 0 |
| ENSRNOP00000005143 | Cxcr4 | 1 | 0 |
| ENSRNOP00000005226 | Rps6kb1 | 1 | 0 |
| ENSRNOP00000005337 | Rptor | 1 | 0 |
| ENSRNOP00000005346 | Pfdn2 | 1 | 0 |
| ENSRNOP00000005347 | Grb2 | 1 | 0 |
| ENSRNOP00000005379 | Chmp6 | 1 | 0 |
| ENSRNOP00000005535 | LOC100911625 | 1 | 0 |
| ENSRNOP00000005576 | Dtl | 1 | 0 |
| ENSRNOP00000005577 | Rps29 | 1 | 0 |
| ENSRNOP00000005601 | LOC100911625 | 1 | 0 |
| ENSRNOP00000005612 | Eno3 | 1 | 0 |
| ENSRNOP00000005641 | Pdk2 | 1 | 0 |
| ENSRNOP00000005835 | Pole2 | 1 | 0 |
| ENSRNOP00000005953 | Bdkrb1 | 1 | 0 |
| ENSRNOP00000006004 | Dpys | 1 | 0 |
| ENSRNOP00000006389 | Plcb1 | 1 | 0 |
| ENSRNOP00000006470 | Gen1 | 1 | 0 |
| ENSRNOP00000006527 | Strn | 1 | 0 |
| ENSRNOP00000006741 | Sh3kbp1 | 1 | 0 |
| ENSRNOP00000006852 | Hus1 | 1 | 0 |
| ENSRNOP00000006900 | Snrpb2 | 1 | 0 |
| ENSRNOP00000006953 | Dync2li1 | 1 | 0 |
| ENSRNOP00000007117 | S1pr4 | 1 | 0 |
| ENSRNOP00000007572 | Grm3 | 1 | 0 |
| ENSRNOP00000007583 | Srsf5 | 1 | 0 |
| ENSRNOP00000007595 | Ppp2r5c | 1 | 0 |
| ENSRNOP00000007624 | Suclg1 | 1 | 0 |
| ENSRNOP00000007649 | Traf3ip3 | 1 | 0 |
| ENSRNOP00000007698 | Gadd45a | 1 | 0 |
| ENSRNOP00000007738 | Pde4b | 1 | 0 |
| ENSRNOP00000007828 | Dlg5 | 1 | 0 |
| ENSRNOP00000008269 | Ccr9 | 1 | 0 |
| ENSRNOP00000008294 | Cxcr6 | 1 | 0 |
| ENSRNOP00000008300 | Pde11a | 1 | 0 |
| ENSRNOP00000008355 | Sf3a1 | 1 | 0 |
| ENSRNOP00000008427 | Srsf6 | 1 | 0 |
| ENSRNOP00000008772 | Ccr1 | 1 | 0 |
| ENSRNOP00000008783 | Ccr1l1 | 1 | 0 |
| ENSRNOP00000008809 | Ccr3 | 1 | 0 |
| ENSRNOP00000008959 | Spo11 | 1 | 0 |
| ENSRNOP00000009124 | Cry1 | 1 | 0 |
| ENSRNOP00000009207 | Mrps16 | 1 | 0 |
| ENSRNOP00000009317 | Htr5a | 1 | 0 |
| ENSRNOP00000009612 | Sstr3 | 1 | 0 |
| ENSRNOP00000009840 | Trib3 | 1 | 0 |
| ENSRNOP00000009944 | Cct6b | 1 | 0 |
| ENSRNOP00000009985 | Cyp51 | 1 | 0 |
| ENSRNOP00000010032 | Npbwr1 | 1 | 0 |
| ENSRNOP00000010142 | Cry2 | 1 | 0 |
| ENSRNOP00000010197 | Rab11b | 1 | 0 |
| ENSRNOP00000010253 | Chmp3 | 1 | 0 |
| ENSRNOP00000010255 | Oprk1 | 1 | 0 |
| ENSRNOP00000010850 | Cnr1 | 1 | 0 |
| ENSRNOP00000010935 | Usp1 | 1 | 0 |
| ENSRNOP00000011123 | Rpa3 | 1 | 0 |
| ENSRNOP00000011155 | Vps39 | 1 | 0 |
| ENSRNOP00000011784 | Eci1 | 1 | 0 |
| ENSRNOP00000011883 | Mtnr1b | 1 | 0 |
| ENSRNOP00000011907 | Chmp5 | 1 | 0 |
| ENSRNOP00000011963 | Gnb5 | 1 | 0 |
| ENSRNOP00000012279 | Dynll2 | 1 | 0 |
| ENSRNOP00000012322 | Adra2c | 1 | 0 |
| ENSRNOP00000012342 | Cnr2 | 1 | 0 |
| ENSRNOP00000012379 | Aplnr | 1 | 0 |
| ENSRNOP00000012400 | Fermt2 | 1 | 0 |
| ENSRNOP00000012432 | Hck | 1 | 0 |
| ENSRNOP00000012492 | Orc1 | 1 | 0 |
| ENSRNOP00000012597 | Cdk6 | 1 | 0 |
| ENSRNOP00000012600 | Tbc1d4 | 1 | 0 |
| ENSRNOP00000012847 | Cct4 | 1 | 0 |
| ENSRNOP00000012936 | Lck | 1 | 0 |
| ENSRNOP00000013058 | Nos3 | 1 | 0 |
| ENSRNOP00000013070 | Rad52 | 1 | 0 |
| ENSRNOP00000013176 | Apex1 | 1 | 0 |
| ENSRNOP00000013184 | Dync1i1 | 1 | 0 |
| ENSRNOP00000013301 | Srsf4 | 1 | 0 |
| ENSRNOP00000013510 | Leo1 | 1 | 0 |
| ENSRNOP00000013618 | Htr1a | 1 | 0 |
| ENSRNOP00000013705 | Ccr4 | 1 | 0 |
| ENSRNOP00000013919 | H2afz | 1 | 0 |
| ENSRNOP00000014020 | Tlr4 | 1 | 0 |
| ENSRNOP00000014034 | Ptger3 | 1 | 0 |
| ENSRNOP00000014084 | Oprd1 | 1 | 0 |
| ENSRNOP00000014167 | Mtor | 1 | 0 |
| ENSRNOP00000014611 | Smptb | 1 | 0 |
| ENSRNOP00000014658 | Hadh | 1 | 0 |
| ENSRNOP00000014701 | Fabp4 | 1 | 0 |
| ENSRNOP00000014747 | Ednrb | 1 | 0 |
| ENSRNOP00000014785 | Itgb1 | 1 | 0 |
| ENSRNOP00000014841 | Fpr1 | 1 | 0 |
| ENSRNOP00000015019 | Ppp2r1a | 1 | 0 |
| ENSRNOP00000015152 | Hnrnpa2b1 | 1 | 0 |
| ENSRNOP00000015179 | Vcl | 1 | 0 |
| ENSRNOP00000015440 | Echdc1 | 1 | 0 |
| ENSRNOP00000015498 | Pde3b | 1 | 0 |
| ENSRNOP00000015518 | Hnrnph2 | 1 | 0 |
| ENSRNOP00000015875 | Ybx1 | 1 | 0 |
| ENSRNOP00000015886 | Cct5 | 1 | 0 |
| ENSRNOP00000015971 | Hnrnpr | 1 | 0 |
| ENSRNOP00000016032 | Fes | 1 | 0 |
| ENSRNOP00000016047 | Htr1d | 1 | 0 |
| ENSRNOP00000016189 | Poli | 1 | 0 |
| ENSRNOP00000016220 | Mapre1 | 1 | 0 |
| ENSRNOP00000016580 | Cxcr5 | 1 | 0 |
| ENSRNOP00000016742 | LOC103694902 | 1 | 0 |
| ENSRNOP00000016751 | Uqcrh | 1 | 0 |
| ENSRNOP00000016904 | Tsc1 | 1 | 0 |
| ENSRNOP00000016946 | Plk4 | 1 | 0 |
| ENSRNOP00000017081 | Mcm3 | 1 | 0 |
| ENSRNOP00000017109 | Lpxn | 1 | 0 |
| ENSRNOP00000017353 | Ehd2 | 1 | 0 |
| ENSRNOP00000017411 | Htr1b | 1 | 0 |
| ENSRNOP00000017421 | Magoh | 1 | 0 |
| ENSRNOP00000017549 | Rpa2 | 1 | 0 |
| ENSRNOP00000017607 | Grm2 | 1 | 0 |
| ENSRNOP00000017794 | Pfdn5 | 1 | 0 |
| ENSRNOP00000017900 | Ireb2 | 1 | 0 |
| ENSRNOP00000017972 | Casp9 | 1 | 0 |
| ENSRNOP00000018147 | Pola1 | 1 | 0 |
| ENSRNOP00000018190 | Rala | 1 | 0 |
| ENSRNOP00000018244 | Foxo1 | 1 | 0 |
| ENSRNOP00000018251 | Gadd45g | 1 | 0 |
| ENSRNOP00000018278 | Kif26a | 1 | 0 |
| ENSRNOP00000018449 | Msh3 | 1 | 0 |
| ENSRNOP00000018556 | Pde7b | 1 | 0 |
| ENSRNOP00000018584 | Adra2b | 1 | 0 |
| ENSRNOP00000018600 | P2ry12 | 1 | 0 |
| ENSRNOP00000018646 | Snrpd1 | 1 | 0 |
| ENSRNOP00000018923 | Cdt1 | 1 | 0 |
| ENSRNOP00000018934 | Acat2l1 | 1 | 0 |
| ENSRNOP00000018952 | Npy1r | 1 | 0 |
| ENSRNOP00000018967 | Nmur2 | 1 | 0 |
| ENSRNOP00000018976 | Npy5r | 1 | 0 |
| ENSRNOP00000019109 | Cxcr2 | 1 | 0 |
| ENSRNOP00000019126 | Sf3b1 | 1 | 0 |
| ENSRNOP00000019288 | Pold2 | 1 | 0 |
| ENSRNOP00000019473 | S1pr3 | 1 | 0 |
| ENSRNOP00000019529 | Hnrnpf | 1 | 0 |
| ENSRNOP00000019561 | Dctn3 | 1 | 0 |
| ENSRNOP00000019579 | Irs1 | 1 | 0 |
| ENSRNOP00000019767 | Fahd1 | 1 | 0 |
| ENSRNOP00000019799 | Lig1 | 1 | 0 |
| ENSRNOP00000019810 | Dhx38 | 1 | 0 |
| ENSRNOP00000020544 | Etfa | 1 | 0 |
| ENSRNOP00000020656 | Lpar3 | 1 | 0 |
| ENSRNOP00000021073 | Tpm4 | 1 | 0 |
| ENSRNOP00000021221 | Snrpd2 | 1 | 0 |
| ENSRNOP00000021289 | Sorbs1 | 1 | 0 |
| ENSRNOP00000021392 | U2af2 | 1 | 0 |
| ENSRNOP00000021475 | Chmp1a | 1 | 0 |
| ENSRNOP00000021538 | Msh2 | 1 | 0 |
| ENSRNOP00000021657 | Pccb | 1 | 0 |
| ENSRNOP00000021745 | Phlpp2 | 1 | 0 |
| ENSRNOP00000021923 | Msh6 | 1 | 0 |
| ENSRNOP00000022179 | Pik3cb | 1 | 0 |
| ENSRNOP00000022190 | Map3k8 | 1 | 0 |
| ENSRNOP00000022256 | Ns5atp9 | 1 | 0 |
| ENSRNOP00000022400 | Galr1 | 1 | 0 |
| ENSRNOP00000022401 | Tln1 | 1 | 0 |
| ENSRNOP00000022744 | Hrh4 | 1 | 0 |
| ENSRNOP00000022963 | Pgm2l1 | 1 | 0 |
| ENSRNOP00000023137 | Poll | 1 | 0 |
| ENSRNOP00000023586 | Chrm4 | 1 | 0 |
| ENSRNOP00000023786 | Eif2s2 | 1 | 0 |
| ENSRNOP00000023854 | Sf3b3 | 1 | 0 |
| ENSRNOP00000024137 | Drd4 | 1 | 0 |
| ENSRNOP00000024375 | Mutyh | 1 | 0 |
| ENSRNOP00000024406 | Ube2i | 1 | 0 |
| ENSRNOP00000024493 | Tpm1 | 1 | 0 |
| ENSRNOP00000024529 | Rbm5 | 1 | 0 |
| ENSRNOP00000024557 | Rad1 | 1 | 0 |
| ENSRNOP00000024875 | Pold3 | 1 | 0 |
| ENSRNOP00000024932 | Per3 | 1 | 0 |
| ENSRNOP00000025024 | Nmur1 | 1 | 0 |
| ENSRNOP00000025203 | Tufm | 1 | 0 |
| ENSRNOP00000025451 | Sstr5 | 1 | 0 |
| ENSRNOP00000025507 | Pold4 | 1 | 0 |
| ENSRNOP00000025564 | Mchr1 | 1 | 0 |
| ENSRNOP00000025615 | Eif1b | 1 | 0 |
| ENSRNOP00000026049 | Polh | 1 | 0 |
| ENSRNOP00000026122 | Hmgcs2 | 1 | 0 |
| ENSRNOP00000026139 | Chtf18 | 1 | 0 |
| ENSRNOP00000026457 | Pde4c | 1 | 0 |
| ENSRNOP00000026558 | Ackr3 | 1 | 0 |
| ENSRNOP00000026586 | Pde2a | 1 | 0 |
| ENSRNOP00000026696 | Hspa9 | 1 | 0 |
| ENSRNOP00000026797 | Pold1 | 1 | 0 |
| ENSRNOP00000026871 | Gadd45b | 1 | 0 |
| ENSRNOP00000027370 | Tubg1 | 1 | 0 |
| ENSRNOP00000027445 | Myl9 | 1 | 0 |
| ENSRNOP00000027507 | Per2 | 1 | 0 |
| ENSRNOP00000027842 | Fen1 | 1 | 0 |
| ENSRNOP00000028141 | Cdc25a | 1 | 0 |
| ENSRNOP00000028411 | Ccnd1 | 1 | 0 |
| ENSRNOP00000029646 | Dync1li2 | 1 | 0 |
| ENSRNOP00000038229 | Chmp2a | 1 | 0 |
| ENSRNOP00000041459 | Eif1 | 1 | 0 |
| ENSRNOP00000041515 | | 1 | 0 |
| ENSRNOP00000043252 | Dync2h1 | 1 | 0 |
| ENSRNOP00000043608 | Hsd17b10 | 1 | 0 |
| ENSRNOP00000044473 | Tpm2 | 1 | 0 |
| ENSRNOP00000045992 | Myl12b | 1 | 0 |
| ENSRNOP00000046345 | Eif1a | 1 | 0 |
| ENSRNOP00000047300 | Rasa1 | 1 | 0 |
| ENSRNOP00000049419 | Eif4a1 | 1 | 0 |
| ENSRNOP00000053093 | Yes1 | 1 | 0 |
| ENSRNOP00000058234 | Clasp1 | 1 | 0 |
| ENSRNOP00000063624 | Prmt1 | 1 | 0 |
| ENSRNOP00000053643 | Chek2 | 1 | 0 |
| ENSRNOP00000035212 | Pfkfb1 | 1 | 0 |
| ENSRNOP00000024863 | Taldo1 | 1 | 0 |
| ENSRNOP00000023252 | Pfkp | 1 | 0 |
| ENSRNOP00000005729 | Pfkfb2 | 1 | 0 |
| ENSRNOP00000061383 | Pfkfb4 | 1 | 0 |
| ENSRNOP00000062965 | Pfkfb3 | 1 | 0 |
| ENSRNOP00000026237 | Gnat2 | 1 | 0 |
| ENSRNOP00000030270 | Gng13 | 1 | 0 |
| ENSRNOP00000067190 | Gng12 | 1 | 0 |
| ENSRNOP00000023791 | Gnat1 | 1 | 0 |
| ENSRNOP00000007032 | Gnat3 | 1 | 0 |
| ENSRNOP00000026539 | Gng3 | 1 | 0 |
| ENSRNOP00000020707 | Gng10 | 1 | 0 |
| ENSRNOP00000026893 | Gng7 | 1 | 0 |
| ENSRNOP00000022441 | Gng8 | 1 | 0 |
| ENSRNOP00000006162 | Cacna1b | 1 | 0 |
| ENSRNOP00000051938 | Gna13 | 1 | 0 |
| ENSRNOP00000051845 | Gcgr | 1 | 0 |
| ENSRNOP00000013244 | Me1 | 1 | 0 |
| ENSRNOP00000023329 | Me3 | 1 | 0 |
| ENSRNOP00000041737 | Me2 | 1 | 0 |
| ENSRNOP00000024471 | Ndufab1 | 1 | 0 |
| ENSRNOP00000027700 | Pklr | 1 | 0 |
| ENSRNOP00000015331 | Pkm | 1 | 0 |
| ENSRNOP00000004917 | Fh | 1 | 0 |
| ENSRNOP00000035601 | Aldh5a1 | 1 | 0 |
| ENSRNOP00000026316 | Pc | 1 | 0 |
| ENSRNOP00000021318 | Stx7 | 1 | 0 |
| ENSRNOP00000025327 | Sec22b | 1 | 0 |
| ENSRNOP00000020693 | Ykt6 | 1 | 0 |
| ENSRNOP00000028535 | Stx3 | 1 | 0 |
| ENSRNOP00000005204 | Stx8 | 1 | 0 |
| ENSRNOP00000054114 | Vamp2 | 1 | 0 |
| ENSRNOP00000007641 | Stx17 | 1 | 0 |
| ENSRNOP00000017301 | Vamp8 | 1 | 0 |
| ENSRNOP00000014810 | Bet1 | 1 | 0 |
| ENSRNOP00000011065 | Vamp7 | 1 | 0 |
| ENSRNOP00000058953 | Vti1a | 1 | 0 |
| ENSRNOP00000040699 | Stx1a | 1 | 0 |
| ENSRNOP00000048364 | Vamp3 | 1 | 0 |
| ENSRNOP00000017227 | Stx12 | 1 | 0 |
| ENSRNOP00000007998 | Snap25 | 1 | 0 |
| ENSRNOP00000025664 | Stx5 | 1 | 0 |
| ENSRNOP00000039276 | LOC100359503 | 1 | 0 |
| ENSRNOP00000044197 | | 1 | 0 |
| ENSRNOP00000067492 | LOC100912571 | 1 | 0 |
| ENSRNOP00000020155 | Pdcd4 | 1 | 0 |
| ENSRNOP00000013719 | Eif4e3 | 1 | 0 |
| ENSRNOP00000062967 | Eif4g2 | 1 | 0 |
| ENSRNOP00000059390 | Eif4g3 | 1 | 0 |
| ENSRNOP00000064962 | Pea15 | 1 | 0 |
| ENSRNOP00000016328 | Dusp4 | 1 | 0 |
| ENSRNOP00000006401 | Ptprr | 1 | 0 |
| ENSRNOP00000019465 | Stat4 | 1 | 0 |
| ENSRNOP00000014604 | Braf | 1 | 0 |
| ENSRNOP00000009151 | Dusp16 | 1 | 0 |
| ENSRNOP00000026207 | Arrb2 | 1 | 0 |
| ENSRNOP00000026760 | Stat3 | 1 | 0 |
| ENSRNOP00000011130 | Lyn | 1 | 0 |
| ENSRNOP00000037346 | Smad3 | 1 | 0 |
| ENSRNOP00000005400 | Dusp10 | 1 | 0 |
| ENSRNOP00000018021 | Iqgap1 | 1 | 0 |
| ENSRNOP00000059867 | Ptpn6 | 1 | 0 |
| ENSRNOP00000046497 | Dusp8 | 1 | 0 |
| ENSRNOP00000007833 | Ptpn7 | 1 | 0 |
| ENSRNOP00000013342 | Spry2 | 1 | 0 |
| ENSRNOP00000044350 | Mitf | 1 | 0 |
| ENSRNOP00000027809 | Rras | 1 | 0 |
| ENSRNOP00000008938 | Rps6ka3 | 1 | 0 |
| ENSRNOP00000019737 | Clta | 1 | 0 |
| ENSRNOP00000036595 | Creb5 | 1 | 0 |
| ENSRNOP00000039672 | Mknk2 | 1 | 0 |
| ENSRNOP00000026354 | Stat5b | 1 | 0 |
| ENSRNOP00000048329 | Smad2 | 1 | 0 |
| ENSRNOP00000043407 | Hmg1l1 | 1 | 0 |
| ENSRNOP00000013522 | Elk1 | 1 | 0 |
| ENSRNOP00000013933 | Map2k1 | 1 | 0 |
| ENSRNOP00000062321 | Camk2g | 1 | 0 |
| ENSRNOP00000060054 | Rps6ka1 | 1 | 0 |
| ENSRNOP00000041940 | Camk2a | 1 | 0 |
| ENSRNOP00000018549 | Dusp2 | 1 | 0 |
| ENSRNOP00000017809 | Rps6ka2 | 1 | 0 |
| ENSRNOP00000010712 | Fos | 1 | 0 |
| ENSRNOP00000047030 | Hsf1 | 1 | 0 |
| ENSRNOP00000016026 | Camk2d | 1 | 0 |
| ENSRNOP00000016704 | Smad5 | 1 | 0 |
| ENSRNOP00000022363 | Hras | 1 | 0 |
| ENSRNOP00000027684 | Erf | 1 | 0 |
| ENSRNOP00000046069 | Arrb1 | 1 | 0 |
| ENSRNOP00000003512 | Rps6ka6 | 1 | 0 |
| ENSRNOP00000014770 | Dusp7 | 1 | 0 |
| ENSRNOP00000052065 | Dusp3 | 1 | 0 |
| ENSRNOP00000018860 | Ptpn5 | 1 | 0 |
| ENSRNOP00000025079 | Smad1 | 1 | 0 |
| ENSRNOP00000018889 | Dusp5 | 1 | 0 |
| ENSRNOP00000054682 | | 1 | 0 |
| ENSRNOP00000026662 | Stat5a | 1 | 0 |
| ENSRNOP00000061535 | Mknk1 | 1 | 0 |
| ENSRNOP00000059567 | Elk4 | 1 | 0 |
| ENSRNOP00000022303 | Mbp | 1 | 0 |
| ENSRNOP00000021310 | Ap2s1 | 1 | 0 |
| ENSRNOP00000006188 | Myc | 1 | 0 |
| ENSRNOP00000032969 | Dusp6 | 1 | 0 |
| ENSRNOP00000005383 | Dusp1 | 1 | 0 |
| ENSRNOP00000041486 | | 1 | 0 |
| ENSRNOP00000027272 | Map2k2 | 1 | 0 |
| ENSRNOP00000007472 | Frs2 | 1 | 0 |
| ENSRNOP00000003630 | Pla2g4a | 1 | 0 |
| ENSRNOP00000043144 | Esr2 | 1 | 0 |
| ENSRNOP00000012616 | Ndufb9 | 1 | 0 |
| ENSRNOP00000018644 | Ndc80 | 1 | 0 |
| ENSRNOP00000042114 | Bub1 | 1 | 0 |
| ENSRNOP00000048964 | Pard3 | 1 | 0 |
| ENSRNOP00000028440 | Cgn | 1 | 0 |
| ENSRNOP00000012924 | Prkci | 1 | 0 |
| ENSRNOP00000024404 | Itgal | 1 | 0 |
| ENSRNOP00000021285 | Prkcz | 1 | 0 |
| ENSRNOP00000029044 | Mllt4 | 1 | 0 |
| ENSRNOP00000051321 | Mpdz | 1 | 0 |
| ENSRNOP00000014988 | Tjp1 | 1 | 0 |
| ENSRNOP00000030279 | Pdha1 | 1 | 0 |
| ENSRNOP00000015217 | Ndufs4 | 1 | 0 |
| ENSRNOP00000027995 | Bckdha | 1 | 0 |
| ENSRNOP00000060990 | Suclg2 | 1 | 0 |
| ENSRNOP00000035059 | Sec63 | 1 | 0 |
| ENSRNOP00000026439 | Dvl1 | 1 | 0 |
| ENSRNOP00000051745 | Nap1l4 | 1 | 0 |
| ENSRNOP00000009894 | Nfkbia | 1 | 0 |
| ENSRNOP00000012022 | Ssrp1 | 1 | 0 |
| ENSRNOP00000024348 | Dvl2 | 1 | 0 |
| ENSRNOP00000015913 | Slc25a5 | 1 | 0 |
| ENSRNOP00000009552 | Pdhx | 1 | 0 |
| ENSRNOP00000016965 | Ndufv2 | 1 | 0 |
| ENSRNOP00000015851 | Ndufs1 | 1 | 0 |
| ENSRNOP00000055942 | Sdhd | 1 | 0 |
| ENSRNOP00000012617 | Abl1 | 1 | 0 |
| ENSRNOP00000011604 | Nefh | 1 | 0 |
| ENSRNOP00000054180 | Cdk5r2 | 1 | 0 |
| ENSRNOP00000044534 | LOC100909750 | 1 | 0 |
| ENSRNOP00000059045 | Dlg4 | 1 | 0 |
| ENSRNOP00000062473 | Cdk5r1 | 1 | 0 |
| ENSRNOP00000034614 | Ppp1r1b | 1 | 0 |
| ENSRNOP00000019735 | Pdcd6 | 1 | 0 |
| ENSRNOP00000022133 | Cep55 | 1 | 0 |
| ENSRNOP00000018194 | Tsg101 | 1 | 0 |
| ENSRNOP00000066181 | Naca | 1 | 0 |
| ENSRNOP00000018455 | Sec61a1 | 1 | 0 |
| ENSRNOP00000036212 | Sec61a2 | 1 | 0 |
| ENSRNOP00000017851 | Ldhc | 1 | 0 |
| ENSRNOP00000017965 | Ldhb | 1 | 0 |
| ENSRNOP00000017468 | Ldha | 1 | 0 |
| ENSRNOP00000059463 | Mfn2 | 1 | 0 |
| ENSRNOP00000024952 | Gdi2 | 1 | 0 |
| ENSRNOP00000024966 | Mon1a | 1 | 0 |
| ENSRNOP00000052539 | Mfn2 | 1 | 0 |
| ENSRNOP00000018961 | Ntrk1 | 1 | 0 |
| ENSRNOP00000061267 | Mtx1 | 1 | 0 |
| ENSRNOP00000027088 | Tomm20 | 1 | 0 |
| ENSRNOP00000051091 | Immt | 1 | 0 |
| ENSRNOP00000060340 | Tomm7 | 1 | 0 |
| ENSRNOP00000019323 | Tomm22 | 1 | 0 |
| ENSRNOP00000018001 | Chchd3 | 1 | 0 |
| ENSRNOP00000058722 | Ttc37 | 1 | 0 |
| ENSRNOP00000026778 | Paf1 | 1 | 0 |
| ENSRNOP00000040635 | Impdh2 | 1 | 0 |
| ENSRNOP00000029334 | Dpysl4 | 1 | 0 |
| ENSRNOP00000031981 | Stk24 | 1 | 0 |
| ENSRNOP00000062778 | Stk3 | 1 | 0 |
| ENSRNOP00000025824 | Cct3 | 1 | 0 |
| ENSRNOP00000023452 | Tubb3 | 1 | 0 |
| ENSRNOP00000024947 | Tubb6 | 1 | 0 |
| ENSRNOP00000024487 | Stk25 | 1 | 0 |
| ENSRNOP00000023611 | Tubb2a | 1 | 0 |
| ENSRNOP00000049998 | Tuba3b | 1 | 0 |
| ENSRNOP00000020932 | LOC100909441 | 1 | 0 |
| ENSRNOP00000044296 | Actb | 1 | 0 |
| ENSRNOP00000021030 | Cct7 | 1 | 0 |
| ENSRNOP00000023582 | Tubb2b | 1 | 0 |
| ENSRNOP00000029234 | Cct2 | 1 | 0 |
| ENSRNOP00000021976 | Strn4 | 1 | 0 |
| ENSRNOP00000066907 | LOC103690168 | 1 | 0 |
| ENSRNOP00000029144 | Aco2 | 1 | 0 |
| ENSRNOP00000044007 | | 1 | 0 |
| ENSRNOP00000028518 | Gapdhs | 1 | 0 |
| ENSRNOP00000065406 | | 1 | 0 |
| ENSRNOP00000043492 | | 1 | 0 |
| ENSRNOP00000055701 | | 1 | 0 |
| ENSRNOP00000062809 | | 1 | 0 |
| ENSRNOP00000061570 | | 1 | 0 |
| ENSRNOP00000054584 | | 1 | 0 |
| ENSRNOP00000043166 | | 1 | 0 |
| ENSRNOP00000044449 | | 1 | 0 |
| ENSRNOP00000064899 | | 1 | 0 |
| ENSRNOP00000063070 | Eno4 | 1 | 0 |
| ENSRNOP00000053537 | | 1 | 0 |
| ENSRNOP00000022610 | Pnmal2 | 1 | 0 |
| ENSRNOP00000041672 | | 1 | 0 |
| ENSRNOP00000033769 | | 1 | 0 |
| ENSRNOP00000035891 | | 1 | 0 |
| ENSRNOP00000050213 | Gapdh-ps2 | 1 | 0 |
| ENSRNOP00000064549 | | 1 | 0 |
| ENSRNOP00000040641 | | 1 | 0 |
| ENSRNOP00000042858 | | 1 | 0 |
| ENSRNOP00000044014 | | 1 | 0 |
| ENSRNOP00000064663 | | 1 | 0 |
| ENSRNOP00000039874 | LOC291543 | 1 | 0 |
| ENSRNOP00000063376 | LOC688739 | 1 | 0 |
| ENSRNOP00000067292 | | 1 | 0 |
| ENSRNOP00000041521 | Cycs | 1 | 0 |
| ENSRNOP00000022747 | Uqcr11 | 1 | 0 |
| ENSRNOP00000065234 | Rab10 | 1 | 0 |
| ENSRNOP00000025649 | Rab14 | 1 | 0 |
| ENSRNOP00000063646 | Oxct1 | 1 | 0 |
| ENSRNOP00000050691 | Acaa1b | 1 | 0 |
| ENSRNOP00000031191 | Fdps | 1 | 0 |
| ENSRNOP00000059561 | Acat2 | 1 | 0 |
| ENSRNOP00000047954 | Mt-cyb | 1 | 0 |
| ENSRNOP00000063449 | Mdm2 | 1 | 0 |
| ENSRNOP00000067118 | Foxg1 | 1 | 0 |
| ENSRNOP00000037374 | Tcl1a | 1 | 0 |
| ENSRNOP00000051338 | Map3k5 | 1 | 0 |
| ENSRNOP00000032902 | Hspb1 | 1 | 0 |
| ENSRNOP00000027677 | Gsk3a | 1 | 0 |
| ENSRNOP00000029993 | Rps6kb2 | 1 | 0 |
| ENSRNOP00000042383 | Foxo4 | 1 | 0 |
| ENSRNOP00000030885 | Ywhaz | 1 | 0 |
| ENSRNOP00000061371 | Inppl1 | 1 | 0 |
| ENSRNOP00000059428 | Ppp2r1b | 1 | 0 |
| ENSRNOP00000025851 | Ikbkb | 1 | 0 |
| ENSRNOP00000030782 | Rictor | 1 | 0 |
| ENSRNOP00000028260 | Them4 | 1 | 0 |
| ENSRNOP00000028143 | Pten | 1 | 0 |
| ENSRNOP00000027478 | Akt1s1 | 1 | 0 |
| ENSRNOP00000034134 | Chuk | 1 | 0 |
| ENSRNOP00000026210 | Pik3r2 | 1 | 0 |
| ENSRNOP00000057770 | Lims2 | 1 | 0 |
| ENSRNOP00000062647 | Lims1 | 1 | 0 |
| ENSRNOP00000027295 | Ilkap | 1 | 0 |
| ENSRNOP00000054688 | Akt3 | 1 | 0 |
| ENSRNOP00000051863 | Tgfb1i1 | 1 | 0 |
| ENSRNOP00000039298 | Snrpb | 1 | 0 |
| ENSRNOP00000033029 | Fus | 1 | 0 |
| ENSRNOP00000026202 | Sf3a2 | 1 | 0 |
| ENSRNOP00000046783 | Hnrnpu | 1 | 0 |
| ENSRNOP00000048698 | Ybx1-ps3 | 1 | 0 |
| ENSRNOP00000043202 | Srsf11 | 1 | 0 |
| ENSRNOP00000057257 | Hnrnpc | 1 | 0 |
| ENSRNOP00000035155 | Srsf7 | 1 | 0 |
| ENSRNOP00000065463 | Lsm2 | 1 | 0 |
| ENSRNOP00000064264 | Vav1 | 1 | 0 |
| ENSRNOP00000028176 | Snrnp70 | 1 | 0 |
| ENSRNOP00000028074 | Cpsf7 | 1 | 0 |
| ENSRNOP00000061368 | Dhx9 | 1 | 0 |
| ENSRNOP00000055962 | Hnrnph1 | 1 | 0 |
| ENSRNOP00000064933 | Srsf1 | 1 | 0 |
| ENSRNOP00000032108 | Hnrnpm | 1 | 0 |
| ENSRNOP00000046491 | Hnrnpd | 1 | 0 |
| ENSRNOP00000026297 | Nudt21 | 1 | 0 |
| ENSRNOP00000042416 | Snrpep2 | 1 | 0 |
| ENSRNOP00000027425 | Hnrnpl | 1 | 0 |
| ENSRNOP00000064990 | Bdkrb2 | 1 | 0 |
| ENSRNOP00000042428 | Fpr3 | 1 | 0 |
| ENSRNOP00000058977 | Lpar2 | 1 | 0 |
| ENSRNOP00000067147 | Npy2r | 1 | 0 |
| ENSRNOP00000051290 | Oprm1 | 1 | 0 |
| ENSRNOP00000064709 | Agtr2 | 1 | 0 |
| ENSRNOP00000032501 | Rxfp3 | 1 | 0 |
| ENSRNOP00000067355 | Sstr1 | 1 | 0 |
| ENSRNOP00000057815 | Pde4a | 1 | 0 |
| ENSRNOP00000034328 | Mtnr1a | 1 | 0 |
| ENSRNOP00000028034 | S1pr2 | 1 | 0 |
| ENSRNOP00000032046 | Gpr17 | 1 | 0 |
| ENSRNOP00000060834 | Pde10a | 1 | 0 |
| ENSRNOP00000047532 | P2ry13 | 1 | 0 |
| ENSRNOP00000043652 | Lpar1 | 1 | 0 |
| ENSRNOP00000028720 | Plcb3 | 1 | 0 |
| ENSRNOP00000052627 | S1pr1 | 1 | 0 |
| ENSRNOP00000028380 | S1pr5 | 1 | 0 |
| ENSRNOP00000045972 | Plcb4 | 1 | 0 |
| ENSRNOP00000043759 | Drd2 | 1 | 0 |
| ENSRNOP00000060812 | Grm4 | 1 | 0 |
| ENSRNOP00000060777 | Pde7a | 1 | 0 |
| ENSRNOP00000047053 | Oprl1 | 1 | 0 |
| ENSRNOP00000066242 | Adra2a | 1 | 0 |
| ENSRNOP00000066690 | Gnb5 | 1 | 0 |
| ENSRNOP00000064558 | C5ar1 | 1 | 0 |
| ENSRNOP00000066231 | Sstr4 | 1 | 0 |
| ENSRNOP00000061809 | Plin1 | 1 | 0 |
| ENSRNOP00000028997 | Ube2u | 1 | 0 |
| ENSRNOP00000061228 | LOC100911727 | 1 | 0 |
| ENSRNOP00000051355 | Dntt | 1 | 0 |
| ENSRNOP00000039931 | Ccna1 | 1 | 0 |
| ENSRNOP00000053270 | Rad51 | 1 | 0 |
| ENSRNOP00000063994 | Chaf1a | 1 | 0 |
| ENSRNOP00000063831 | Dnmt1 | 1 | 0 |
| ENSRNOP00000033080 | Sprtn | 1 | 0 |
| ENSRNOP00000032177 | Cdc6 | 1 | 0 |
| ENSRNOP00000054053 | Rev3l | 1 | 0 |
| ENSRNOP00000060925 | Apex2 | 1 | 0 |
| ENSRNOP00000062102 | Ube2v2 | 1 | 0 |
| ENSRNOP00000062763 | Kmt5a | 1 | 0 |
| ENSRNOP00000053707 | Ung | 1 | 0 |
| ENSRNOP00000034638 | Poln | 1 | 0 |
| ENSRNOP00000056107 | Ercc5 | 1 | 0 |
| ENSRNOP00000053576 | LOC100362927 | 1 | 0 |
| ENSRNOP00000065285 | RGD1561853 | 1 | 0 |
| ENSRNOP00000061834 | Mlh1 | 1 | 0 |
| ENSRNOP00000067214 | Zfpl1 | 1 | 0 |
| ENSRNOP00000036568 | Rbbp4 | 1 | 0 |
| ENSRNOP00000053086 | Tyms | 1 | 0 |
| ENSRNOP00000058920 | Pole | 1 | 0 |
| ENSRNOP00000062524 | Mcm5 | 1 | 0 |
| ENSRNOP00000058174 | Ube2a | 1 | 0 |
| ENSRNOP00000064704 | Ccnd3 | 1 | 0 |
| ENSRNOP00000059094 | Cdc7 | 1 | 0 |
| ENSRNOP00000059807 | Rev1 | 1 | 0 |
| ENSRNOP00000032191 | Cdk2 | 1 | 0 |
| ENSRNOP00000055596 | Wrn | 1 | 0 |
| ENSRNOP00000063400 | Rad18 | 1 | 0 |
| ENSRNOP00000028898 | Mcm8 | 1 | 0 |
| ENSRNOP00000034754 | Cdk4 | 1 | 0 |
| ENSRNOP00000040703 | Rps29 | 1 | 0 |
| ENSRNOP00000043270 | Rps29 | 1 | 0 |
| ENSRNOP00000044909 | Rps29 | 1 | 0 |
| ENSRNOP00000059833 | Tipin | 1 | 0 |
| ENSRNOP00000053083 | Tipinl1 | 1 | 0 |
| ENSRNOP00000066206 | Cdc45 | 1 | 0 |
| ENSRNOP00000053964 | Per1 | 1 | 0 |
| ENSRNOP00000061074 | Vta1 | 1 | 0 |
| ENSRNOP00000063822 | Calm2 | 1 | 0 |
| ENSRNOP00000060919 | Dync1i2 | 1 | 0 |
| ENSRNOP00000066312 | Dync1h1 | 1 | 0 |
| ENSRNOP00000059841 | Dnah1 | 1 | 0 |
| ENSRNOP00000061342 | Dynll1 | 1 | 0 |

| **Table 9.** GO enrichment of BAT protein-protein interaction network | | | | | |
| --- | --- | --- | --- | --- | --- |
| **Pathway** | **Total** | **Expected** | **Hits** | **P.Value** | **FDR** |
| **Biological Process** | |  |  |  |  |
| Chromatin assembly or disassembly | 248 | 9.1 | 77 | 4.48E-51 | 3.02E-48 |
| Nucleosome assembly | 42 | 1.54 | 14 | 1.35E-10 | 4.55E-08 |
| Developmental growth | 31 | 1.14 | 12 | 3.84E-10 | 8.65E-08 |
| Transcription, DNA_dependent | 21 | 0.771 | 9 | 2.21E-08 | 3.73E-06 |
| Sensory perception of taste | 5 | 0.183 | 5 | 6.51E-08 | 8.02E-06 |
| Regulation of transcription, DNA_dependent | 434 | 15.9 | 40 | 7.13E-08 | 8.02E-06 |
| Hemostasis | 80 | 2.94 | 14 | 1.07E-06 | 0.000103 |
| Keratinocyte differentiation | 345 | 12.7 | 32 | 1.33E-06 | 0.000112 |
| Cellular localization | 12 | 0.44 | 6 | 1.81E-06 | 0.00013 |
| Negative regulation of DNA metabolic process | 25 | 0.917 | 8 | 1.92E-06 | 0.00013 |
| Secretion by cell | 20 | 0.734 | 7 | 4.37E-06 | 0.000268 |
| N_acetylglucosamine metabolic process | 14 | 0.514 | 6 | 5.52E-06 | 0.00031 |
| Glucosamine metabolic process | 70 | 2.57 | 12 | 7.90E-06 | 0.00041 |
| Cytokine biosynthetic process | 151 | 5.54 | 18 | 1.03E-05 | 0.000499 |
| Regulation of cyclin_dependent protein kinase activity | 17 | 0.624 | 6 | 2.07E-05 | 0.000931 |
| DNA damage response, signal transduction by p53 class mediator | 11 | 0.404 | 5 | 2.50E-05 | 0.00106 |
| Heterophilic cell_cell adhesion | 199 | 7.3 | 20 | 4.29E-05 | 0.0017 |
| Anatomical structure formation involved in morphogenesis | 38 | 1.39 | 8 | 5.72E-05 | 0.00214 |
| DNA_dependent transcription, initiation | 99 | 3.63 | 13 | 6.39E-05 | 0.00227 |
| Negative regulation of MAP kinase activity | 102 | 3.74 | 13 | 8.75E-05 | 0.00295 |
| Protein complex disassembly | 4 | 0.147 | 3 | 0.000191 | 0.00614 |
| Xenobiotic metabolic process | 191 | 7.01 | 18 | 0.000232 | 0.00712 |
| Multicellular organismal development | 17 | 0.624 | 5 | 0.000279 | 0.00819 |
| Establishment of organelle localization | 29 | 1.06 | 6 | 0.000546 | 0.0154 |
| Tyrosine phosphorylation of STAT protein | 41 | 1.5 | 7 | 0.000651 | 0.0176 |
| Regulation of transcription from RNA polymerase II promoter | 86 | 3.16 | 10 | 0.00117 | 0.0303 |
| Regulation of actin filament length | 35 | 1.28 | 6 | 0.00155 | 0.0387 |
| Muscle organ development | 36 | 1.32 | 6 | 0.0018 | 0.0412 |
| Interleukin_1 secretion | 36 | 1.32 | 6 | 0.0018 | 0.0412 |
| DNA damage checkpoint | 62 | 2.28 | 8 | 0.00183 | 0.0412 |
| **Molecular Function** | |  |  |  |  |
| RNA binding | 270 | 10.2 | 88 | 1.89E-59 | 6.26E-57 |
| Steroid dehydrogenase activity | 334 | 12.7 | 45 | 7.97E-14 | 1.32E-11 |
| RNA helicase activity | 49 | 1.86 | 17 | 1.08E-12 | 1.19E-10 |
| Transferase activity, transferring acyl groups | 253 | 9.59 | 35 | 2.52E-11 | 2.09E-09 |
| Neuropeptide hormone activity | 1270 | 48.3 | 92 | 4.53E-10 | 3.00E-08 |
| DNA_directed DNA polymerase activity | 26 | 0.986 | 11 | 9.51E-10 | 5.24E-08 |
| Transcription cofactor activity | 356 | 13.5 | 36 | 7.27E-08 | 3.44E-06 |
| Cation_transporting ATPase activity | 64 | 2.43 | 13 | 6.31E-07 | 2.61E-05 |
| Antioxidant activity | 72 | 2.73 | 13 | 2.58E-06 | 9.50E-05 |
| Exopeptidase activity | 103 | 3.91 | 15 | 7.25E-06 | 0.00024 |
| DNA helicase activity | 15 | 0.569 | 6 | 1.07E-05 | 0.000323 |
| Nuclease activity | 225 | 8.53 | 23 | 1.47E-05 | 0.000406 |
| Lipase activity | 69 | 2.62 | 11 | 5.14E-05 | 0.00131 |
| Ion binding | 21 | 0.796 | 6 | 9.59E-05 | 0.00227 |
| Translation initiation factor activity | 162 | 6.14 | 17 | 0.000138 | 0.00306 |
| G_protein coupled receptor binding | 53 | 2.01 | 9 | 0.00015 | 0.0031 |
| Nucleotide binding | 273 | 10.4 | 23 | 0.000289 | 0.00541 |
| Damaged DNA binding | 46 | 1.74 | 8 | 0.000294 | 0.00541 |
| Carbon_carbon lyase activity | 17 | 0.645 | 5 | 0.000325 | 0.00566 |
| Endonuclease activity | 279 | 10.6 | 23 | 0.000394 | 0.00652 |
| Transcription corepressor activity | 101 | 3.83 | 12 | 0.000425 | 0.0067 |
| Hydrolase activity, hydrolyzing O_glycosyl compounds | 92 | 3.49 | 11 | 0.000694 | 0.0102 |
| Nucleobase_containing compound transmembrane transporter activity | 291 | 11 | 23 | 0.00071 | 0.0102 |
| Kinase activity | 212 | 8.04 | 18 | 0.00118 | 0.0163 |
| Protein binding, bridging | 62 | 2.35 | 8 | 0.00225 | 0.0298 |
| Organic anion transmembrane transporter activity | 65 | 2.46 | 8 | 0.00304 | 0.0387 |
| Ubiquitin binding | 17 | 0.645 | 4 | 0.00328 | 0.0402 |
| Hydrolase activity, acting on acid anhydrides | 512 | 19.4 | 32 | 0.00376 | 0.0438 |
| Low_density lipoprotein particle binding | 9 | 0.341 | 3 | 0.00383 | 0.0438 |
| Regulation of DNA_dependent transcription, elongation | 41 | 1.55 | 6 | 0.00419 | 0.0462 |
| **Cellular component** | |  |  |  |  |
| Nucleolus | 1280 | 38.6 | 113 | 1.77E-26 | 3.11E-24 |
| Microtubule organizing center | 109 | 3.3 | 29 | 9.09E-20 | 8.00E-18 |
| Mitochondrial envelope | 235 | 7.11 | 40 | 3.74E-19 | 2.20E-17 |
| Centrosome | 1180 | 35.7 | 92 | 1.32E-17 | 5.83E-16 |
| Vesicle membrane | 1670 | 50.5 | 102 | 1.65E-12 | 5.81E-11 |
| Nucleus | 3830 | 116 | 179 | 3.00E-11 | 8.79E-10 |
| Nucleoplasm | 1450 | 44 | 89 | 6.19E-11 | 1.56E-09 |
| Spindle | 13 | 0.394 | 8 | 7.48E-10 | 1.65E-08 |
| Integral to organelle membrane | 411 | 12.4 | 37 | 3.23E-09 | 6.32E-08 |
| Chromosome | 4070 | 123 | 179 | 5.85E-09 | 1.03E-07 |
| Nuclear lumen | 71 | 2.15 | 14 | 2.21E-08 | 3.54E-07 |
| Cell_cell junction | 307 | 9.29 | 29 | 6.05E-08 | 8.61E-07 |
| Endomembrane system | 14 | 0.424 | 7 | 6.36E-08 | 8.61E-07 |
| Golgi stack | 376 | 11.4 | 32 | 1.43E-07 | 1.80E-06 |
| Eukaryotic translation initiation factor 3 complex | 191 | 5.78 | 20 | 1.43E-06 | 1.67E-05 |
| Clathrin_coated vesicle | 85 | 2.57 | 13 | 1.54E-06 | 1.69E-05 |
| Pore complex | 40 | 1.21 | 9 | 2.34E-06 | 2.42E-05 |
| Vesicle coat | 292 | 8.84 | 25 | 3.05E-06 | 2.98E-05 |
| Cell surface | 128 | 3.87 | 13 | 0.000135 | 0.00123 |
| Mitochondrial respiratory chain | 112 | 3.39 | 12 | 0.000146 | 0.00123 |
| Membrane coat | 222 | 6.72 | 18 | 0.000147 | 0.00123 |
| Mitochondrial matrix | 18 | 0.545 | 5 | 0.000154 | 0.00123 |
| Transport vesicle | 96 | 2.91 | 10 | 0.000646 | 0.00494 |
| Cell leading edge | 260 | 7.87 | 18 | 0.000982 | 0.00698 |
| Extracellular matrix | 68 | 2.06 | 8 | 0.000998 | 0.00698 |
| Apical junction complex | 467 | 14.1 | 27 | 0.00103 | 0.00698 |
| Golgi_associated vesicle | 90 | 2.72 | 9 | 0.00159 | 0.0104 |
| U12_type spliceosomal complex | 612 | 18.5 | 32 | 0.00192 | 0.0121 |
| Mitochondrion | 89 | 2.69 | 8 | 0.00551 | 0.0334 |
| Mitochondrial inner membrane | 39 | 1.18 | 5 | 0.00613 | 0.036 |
| Kinetochore | 73 | 2.21 | 7 | 0.00649 | 0.0369 |
| Anchored to membrane | 112 | 3.39 | 9 | 0.00693 | 0.0381 |
| Endosome | 96 | 2.91 | 8 | 0.00862 | 0.0456 |
| Cortical cytoskeleton | 137 | 4.15 | 10 | 0.00881 | 0.0456 |
| Sarcomere | 43 | 1.3 | 5 | 0.00929 | 0.0467 |

| **Table 10.** List of nodes in protein-protein interaction network in WAT with exercise training | | | |
| --- | --- | --- | --- |
| **Id** | **Label** | **Degree** | **Betweenness** |
| ENSRNOP00000013462 | Rpl4 | 403 | 77156.57 |
| ENSRNOP00000019247 | Rpl27a | 368 | 48351.63 |
| ENSRNOP00000014849 | Rpl29 | 278 | 19304.72 |
| ENSRNOP00000060568 | Rps28 | 249 | 20954.5 |
| ENSRNOP00000026462 | Psmb10 | 60 | 42504.83 |
| ENSRNOP00000017421 | Magoh | 45 | 29814 |
| ENSRNOP00000026279 | Psme2 | 33 | 17171.42 |
| ENSRNOP00000059076 | Dctn1 | 24 | 10456.5 |
| ENSRNOP00000020748 | Rab8a | 14 | 18059.5 |
| ENSRNOP00000032902 | Hspb1 | 12 | 32101.83 |
| ENSRNOP00000023256 | Slc2a4 | 9 | 22219.5 |
| ENSRNOP00000027073 | Uba52 | 6 | 39925.52 |
| ENSRNOP00000041134 | Usp7 | 6 | 2935 |
| ENSRNOP00000059012 | Srsf2 | 5 | 2350 |
| ENSRNOP00000004867 | Sumo2 | 4 | 1764 |
| ENSRNOP00000000603 | Rpl10a | 4 | 10.4 |
| ENSRNOP00000001265 | Rpl21 | 4 | 10.4 |
| ENSRNOP00000002194 | Rpl24 | 4 | 10.4 |
| ENSRNOP00000004303 | RGD1559951 | 4 | 10.4 |
| ENSRNOP00000005471 | Rpl23 | 4 | 10.4 |
| ENSRNOP00000005872 | Rps27a | 4 | 10.4 |
| ENSRNOP00000006359 | Rpl19 | 4 | 10.4 |
| ENSRNOP00000006754 | Rpl7a | 4 | 10.4 |
| ENSRNOP00000007683 | | 4 | 10.4 |
| ENSRNOP00000009988 | RGD1560831 | 4 | 10.4 |
| ENSRNOP00000010383 | LOC100911372 | 4 | 10.4 |
| ENSRNOP00000010759 | Rpl15 | 4 | 10.4 |
| ENSRNOP00000011314 | Rps20 | 4 | 10.4 |
| ENSRNOP00000011333 | Rps7 | 4 | 10.4 |
| ENSRNOP00000013868 | LOC100361180 | 4 | 10.4 |
| ENSRNOP00000015893 | | 4 | 10.4 |
| ENSRNOP00000016329 | Rps3a | 4 | 10.4 |
| ENSRNOP00000017230 | RGD1565317 | 4 | 10.4 |
| ENSRNOP00000018820 | Rplp1 | 4 | 10.4 |
| ENSRNOP00000020635 | LOC100359922 | 4 | 10.4 |
| ENSRNOP00000021161 | | 4 | 10.4 |
| ENSRNOP00000022184 | Rps12 | 4 | 10.4 |
| ENSRNOP00000022348 | Rps23 | 4 | 10.4 |
| ENSRNOP00000023368 | Rpl38 | 4 | 10.4 |
| ENSRNOP00000023935 | Rps3 | 4 | 10.4 |
| ENSRNOP00000024678 | Rps15a | 4 | 10.4 |
| ENSRNOP00000025217 | Rpl17 | 4 | 10.4 |
| ENSRNOP00000025888 | Rps17 | 4 | 10.4 |
| ENSRNOP00000026576 | Rps16 | 4 | 10.4 |
| ENSRNOP00000027086 | Cnbd2 | 4 | 10.4 |
| ENSRNOP00000027226 | LOC100360573 | 4 | 10.4 |
| ENSRNOP00000028060 | Rpl27 | 4 | 10.4 |
| ENSRNOP00000028481 | LOC100360647 | 4 | 10.4 |
| ENSRNOP00000028555 | Rpl18 | 4 | 10.4 |
| ENSRNOP00000031121 | LOC100362366 | 4 | 10.4 |
| ENSRNOP00000032635 | LOC100360449 | 4 | 10.4 |
| ENSRNOP00000033369 | | 4 | 10.4 |
| ENSRNOP00000034364 | Rpl17 | 4 | 10.4 |
| ENSRNOP00000034657 | LOC687780 | 4 | 10.4 |
| ENSRNOP00000034767 | RGD1359290 | 4 | 10.4 |
| ENSRNOP00000036391 | Rpl23a | 4 | 10.4 |
| ENSRNOP00000036514 | Rpl5 | 4 | 10.4 |
| ENSRNOP00000037110 | Rpl11 | 4 | 10.4 |
| ENSRNOP00000038065 | Rpl6 | 4 | 10.4 |
| ENSRNOP00000039111 | | 4 | 10.4 |
| ENSRNOP00000039179 | | 4 | 10.4 |
| ENSRNOP00000039287 | LOC102550668 | 4 | 10.4 |
| ENSRNOP00000039774 | RGD1560017 | 4 | 10.4 |
| ENSRNOP00000039786 | | 4 | 10.4 |
| ENSRNOP00000040548 | RGD1563570 | 4 | 10.4 |
| ENSRNOP00000040955 | LOC103691423 | 4 | 10.4 |
| ENSRNOP00000040966 | Rpl10l | 4 | 10.4 |
| ENSRNOP00000041191 | | 4 | 10.4 |
| ENSRNOP00000041199 | | 4 | 10.4 |
| ENSRNOP00000041263 | | 4 | 10.4 |
| ENSRNOP00000041435 | | 4 | 10.4 |
| ENSRNOP00000041458 | RGD1562381 | 4 | 10.4 |
| ENSRNOP00000041530 | | 4 | 10.4 |
| ENSRNOP00000041774 | RGD1564730 | 4 | 10.4 |
| ENSRNOP00000041817 | Rpl9 | 4 | 10.4 |
| ENSRNOP00000041920 | RGD1559955 | 4 | 10.4 |
| ENSRNOP00000041966 | Rpl21 | 4 | 10.4 |
| ENSRNOP00000042022 | LOC690468 | 4 | 10.4 |
| ENSRNOP00000042031 | LOC100360647 | 4 | 10.4 |
| ENSRNOP00000042242 | LOC100909878 | 4 | 10.4 |
| ENSRNOP00000042277 | | 4 | 10.4 |
| ENSRNOP00000042286 | LOC680512 | 4 | 10.4 |
| ENSRNOP00000042288 | LOC682793 | 4 | 10.4 |
| ENSRNOP00000042454 | | 4 | 10.4 |
| ENSRNOP00000042560 | | 4 | 10.4 |
| ENSRNOP00000042567 | | 4 | 10.4 |
| ENSRNOP00000042941 | LOC500148 | 4 | 10.4 |
| ENSRNOP00000044063 | LOC686066 | 4 | 10.4 |
| ENSRNOP00000044605 | LOC100361060 | 4 | 10.4 |
| ENSRNOP00000045195 | LOC100360439 | 4 | 10.4 |
| ENSRNOP00000045213 | RGD1561636 | 4 | 10.4 |
| ENSRNOP00000045335 | | 4 | 10.4 |
| ENSRNOP00000045390 | RGD1562265 | 4 | 10.4 |
| ENSRNOP00000045739 | | 4 | 10.4 |
| ENSRNOP00000045912 | | 4 | 10.4 |
| ENSRNOP00000046070 | | 4 | 10.4 |
| ENSRNOP00000046409 | | 4 | 10.4 |
| ENSRNOP00000046578 | | 4 | 10.4 |
| ENSRNOP00000046600 | LOC306079 | 4 | 10.4 |
| ENSRNOP00000046669 | | 4 | 10.4 |
| ENSRNOP00000046737 | LOC691716 | 4 | 10.4 |
| ENSRNOP00000047281 | Rps27a-ps6 | 4 | 10.4 |
| ENSRNOP00000047511 | | 4 | 10.4 |
| ENSRNOP00000047513 | Rpl37a | 4 | 10.4 |
| ENSRNOP00000048019 | RGD1563145 | 4 | 10.4 |
| ENSRNOP00000048495 | | 4 | 10.4 |
| ENSRNOP00000048620 | RGD1561453 | 4 | 10.4 |
| ENSRNOP00000048664 | Rps27a | 4 | 10.4 |
| ENSRNOP00000048808 | Rpl21 | 4 | 10.4 |
| ENSRNOP00000048903 | LOC100363469 | 4 | 10.4 |
| ENSRNOP00000049028 | LOC680353 | 4 | 10.4 |
| ENSRNOP00000049286 | Rps15al2 | 4 | 10.4 |
| ENSRNOP00000049416 | RGD1563835 | 4 | 10.4 |
| ENSRNOP00000049709 | | 4 | 10.4 |
| ENSRNOP00000049710 | | 4 | 10.4 |
| ENSRNOP00000049831 | | 4 | 10.4 |
| ENSRNOP00000050047 | | 4 | 10.4 |
| ENSRNOP00000050202 | LOC682793 | 4 | 10.4 |
| ENSRNOP00000050353 | LOC682793 | 4 | 10.4 |
| ENSRNOP00000050533 | RGD1563705 | 4 | 10.4 |
| ENSRNOP00000050941 | Rps24 | 4 | 10.4 |
| ENSRNOP00000051016 | LOC100364191 | 4 | 10.4 |
| ENSRNOP00000051135 | Rpl6-ps1 | 4 | 10.4 |
| ENSRNOP00000051203 | | 4 | 10.4 |
| ENSRNOP00000051312 | Rpl21 | 4 | 10.4 |
| ENSRNOP00000051318 | LOC100359563 | 4 | 10.4 |
| ENSRNOP00000051332 | | 4 | 10.4 |
| ENSRNOP00000051743 | | 4 | 10.4 |
| ENSRNOP00000053082 | Rpl5l1 | 4 | 10.4 |
| ENSRNOP00000053160 | | 4 | 10.4 |
| ENSRNOP00000053991 | | 4 | 10.4 |
| ENSRNOP00000054497 | LOC100912027 | 4 | 10.4 |
| ENSRNOP00000054699 | RGD1560069 | 4 | 10.4 |
| ENSRNOP00000055334 | RGD1565767 | 4 | 10.4 |
| ENSRNOP00000055393 | | 4 | 10.4 |
| ENSRNOP00000055671 | | 4 | 10.4 |
| ENSRNOP00000058757 | | 4 | 10.4 |
| ENSRNOP00000058934 | Rpl36 | 4 | 10.4 |
| ENSRNOP00000059382 | Rps24 | 4 | 10.4 |
| ENSRNOP00000060476 | LOC680579 | 4 | 10.4 |
| ENSRNOP00000065066 | LOC100365839 | 4 | 10.4 |
| ENSRNOP00000066016 | LOC100912027 | 4 | 10.4 |
| ENSRNOP00000064959 | LOC103692519 | 4 | 10.4 |
| ENSRNOP00000065901 | | 4 | 10.4 |
| ENSRNOP00000067446 | LOC100909911 | 4 | 10.4 |
| ENSRNOP00000061370 | | 4 | 10.4 |
| ENSRNOP00000067411 | | 4 | 10.4 |
| ENSRNOP00000067793 | | 4 | 10.4 |
| ENSRNOP00000066511 | | 4 | 10.4 |
| ENSRNOP00000064524 | RGD1563124 | 4 | 10.4 |
| ENSRNOP00000066077 | LOC100362684 | 4 | 10.4 |
| ENSRNOP00000065886 | LOC100359951 | 4 | 10.4 |
| ENSRNOP00000063201 | RGD1561102 | 4 | 10.4 |
| ENSRNOP00000065423 | | 4 | 10.4 |
| ENSRNOP00000064082 | LOC100360491 | 4 | 10.4 |
| ENSRNOP00000060629 | RGD1561870 | 4 | 10.4 |
| ENSRNOP00000064320 | LOC100365810 | 4 | 10.4 |
| ENSRNOP00000066866 | LOC103690796 | 4 | 10.4 |
| ENSRNOP00000062764 | | 4 | 10.4 |
| ENSRNOP00000064745 | | 4 | 10.4 |
| ENSRNOP00000061747 | Rpl14 | 4 | 10.4 |
| ENSRNOP00000064822 | RGD1565048 | 4 | 10.4 |
| ENSRNOP00000064270 | RGD1561137 | 4 | 10.4 |
| ENSRNOP00000066050 | LOC100364116 | 4 | 10.4 |
| ENSRNOP00000063355 | LOC100361259 | 4 | 10.4 |
| ENSRNOP00000067572 | Rps6 | 4 | 10.4 |
| ENSRNOP00000066863 | RGD1560821 | 4 | 10.4 |
| ENSRNOP00000064461 | | 4 | 10.4 |
| ENSRNOP00000065877 | LOC102548369 | 4 | 10.4 |
| ENSRNOP00000067354 | LOC100361079 | 4 | 10.4 |
| ENSRNOP00000066750 | LOC100909911 | 4 | 10.4 |
| ENSRNOP00000065827 | | 4 | 10.4 |
| ENSRNOP00000066260 | LOC103692519 | 4 | 10.4 |
| ENSRNOP00000066592 | | 4 | 10.4 |
| ENSRNOP00000063142 | | 4 | 10.4 |
| ENSRNOP00000052049 | Eftud2 | 3 | 28944 |
| ENSRNOP00000013997 | Psmc5 | 3 | 15870 |
| ENSRNOP00000025819 | Psmc4 | 3 | 15870 |
| ENSRNOP00000047840 | Tp53 | 3 | 4668 |
| ENSRNOP00000004278 | Rps4x | 3 | 8.54 |
| ENSRNOP00000004583 | | 3 | 8.54 |
| ENSRNOP00000006662 | RGD1566369 | 3 | 8.54 |
| ENSRNOP00000007194 | | 3 | 8.54 |
| ENSRNOP00000017602 | LOC100359763 | 3 | 8.54 |
| ENSRNOP00000017738 | | 3 | 8.54 |
| ENSRNOP00000019508 | Rps2 | 3 | 8.54 |
| ENSRNOP00000022897 | Rps27 | 3 | 8.54 |
| ENSRNOP00000026528 | Rps5 | 3 | 8.54 |
| ENSRNOP00000027246 | LOC100910336 | 3 | 8.54 |
| ENSRNOP00000033144 | Rps25 | 3 | 8.54 |
| ENSRNOP00000033162 | LOC500594 | 3 | 8.54 |
| ENSRNOP00000036343 | LOC688473 | 3 | 8.54 |
| ENSRNOP00000036690 | LOC684988 | 3 | 8.54 |
| ENSRNOP00000036943 | | 3 | 8.54 |
| ENSRNOP00000039003 | | 3 | 8.54 |
| ENSRNOP00000039845 | Rps19l1 | 3 | 8.54 |
| ENSRNOP00000040306 | RGD1563613 | 3 | 8.54 |
| ENSRNOP00000041853 | LOC297756 | 3 | 8.54 |
| ENSRNOP00000042092 | Rsl1d1 | 3 | 8.54 |
| ENSRNOP00000042902 | LOC100360843 | 3 | 8.54 |
| ENSRNOP00000043543 | LOC103690015 | 3 | 8.54 |
| ENSRNOP00000043988 | Rps4x-ps9 | 3 | 8.54 |
| ENSRNOP00000044301 | LOC688899 | 3 | 8.54 |
| ENSRNOP00000044563 | LOC680646 | 3 | 8.54 |
| ENSRNOP00000045516 | Wdr31 | 3 | 8.54 |
| ENSRNOP00000047391 | RGD1564597 | 3 | 8.54 |
| ENSRNOP00000047760 | | 3 | 8.54 |
| ENSRNOP00000049546 | Rps4y2 | 3 | 8.54 |
| ENSRNOP00000052873 | LOC100911847 | 3 | 8.54 |
| ENSRNOP00000055288 | RGD1562399 | 3 | 8.54 |
| ENSRNOP00000056260 | LOC100911847 | 3 | 8.54 |
| ENSRNOP00000056750 | LOC100364509 | 3 | 8.54 |
| ENSRNOP00000057658 | Rps2-ps6 | 3 | 8.54 |
| ENSRNOP00000065281 | LOC685085 | 3 | 8.54 |
| ENSRNOP00000066792 | LOC100364509 | 3 | 8.54 |
| ENSRNOP00000065999 | LOC100911337 | 3 | 8.54 |
| ENSRNOP00000061442 | LOC100362339 | 3 | 8.54 |
| ENSRNOP00000067129 | Rps27l | 3 | 8.54 |
| ENSRNOP00000064853 | LOC100911337 | 3 | 8.54 |
| ENSRNOP00000066362 | LOC100362987 | 3 | 8.54 |
| ENSRNOP00000067470 | LOC683961 | 3 | 8.54 |
| ENSRNOP00000066548 | | 3 | 8.54 |
| ENSRNOP00000065487 | LOC100363452 | 3 | 8.54 |
| ENSRNOP00000065173 | | 3 | 8.54 |
| ENSRNOP00000063451 | RGD1559724 | 3 | 8.54 |
| ENSRNOP00000066808 | LOC100910336 | 3 | 8.54 |
| ENSRNOP00000025224 | Rpsa | 3 | 6.31 |
| ENSRNOP00000044806 | RGD1565117 | 3 | 6.31 |
| ENSRNOP00000048116 | LOC100361854 | 3 | 6.31 |
| ENSRNOP00000027780 | Rps27a-ps12 | 3 | 5.96 |
| ENSRNOP00000035156 | Rps15 | 3 | 5.96 |
| ENSRNOP00000048979 | Rps27a-ps5 | 3 | 5.96 |
| ENSRNOP00000062631 | Rps15-ps2 | 3 | 5.96 |
| ENSRNOP00000001518 | Rplp0 | 3 | 0 |
| ENSRNOP00000002177 | | 3 | 0 |
| ENSRNOP00000004213 | | 3 | 0 |
| ENSRNOP00000005511 | | 3 | 0 |
| ENSRNOP00000005588 | Rpl26 | 3 | 0 |
| ENSRNOP00000009046 | Rpl34 | 3 | 0 |
| ENSRNOP00000009431 | Rpl7 | 3 | 0 |
| ENSRNOP00000011244 | LOC688684 | 3 | 0 |
| ENSRNOP00000012255 | LOC690096 | 3 | 0 |
| ENSRNOP00000014493 | Rpl32 | 3 | 0 |
| ENSRNOP00000014905 | LOC100360057 | 3 | 0 |
| ENSRNOP00000015408 | RGD1565894 | 3 | 0 |
| ENSRNOP00000015756 | Rpl22l1 | 3 | 0 |
| ENSRNOP00000019162 | Rpl35 | 3 | 0 |
| ENSRNOP00000044553 | LOC103694169 | 3 | 0 |
| ENSRNOP00000038214 | LOC100911575 | 3 | 0 |
| ENSRNOP00000048252 | LOC100911426 | 3 | 0 |
| ENSRNOP00000059662 | LOC103690821 | 3 | 0 |
| ENSRNOP00000045098 | LOC103694169 | 3 | 0 |
| ENSRNOP00000061874 | | 3 | 0 |
| ENSRNOP00000039155 | Rpl37 | 3 | 0 |
| ENSRNOP00000043092 | RGD1561317 | 3 | 0 |
| ENSRNOP00000054740 | LOC100362751 | 3 | 0 |
| ENSRNOP00000050175 | | 3 | 0 |
| ENSRNOP00000067881 | RGD1564378 | 3 | 0 |
| ENSRNOP00000067080 | LOC100360117 | 3 | 0 |
| ENSRNOP00000044275 | LOC100910017 | 3 | 0 |
| ENSRNOP00000055790 | LOC103692785 | 3 | 0 |
| ENSRNOP00000050700 | LOC690335 | 3 | 0 |
| ENSRNOP00000041638 | Rpl32 | 3 | 0 |
| ENSRNOP00000054398 | LOC100362027 | 3 | 0 |
| ENSRNOP00000048999 | Rpl31l4 | 3 | 0 |
| ENSRNOP00000049665 | LOC103690821 | 3 | 0 |
| ENSRNOP00000049713 | LOC103693375 | 3 | 0 |
| ENSRNOP00000051188 | Rpl35al1 | 3 | 0 |
| ENSRNOP00000065371 | LOC100911426 | 3 | 0 |
| ENSRNOP00000058614 | RGD1564095 | 3 | 0 |
| ENSRNOP00000042633 | | 3 | 0 |
| ENSRNOP00000059772 | RGD1565566 | 3 | 0 |
| ENSRNOP00000041209 | Rpl35a | 3 | 0 |
| ENSRNOP00000043004 | RGD1564606 | 3 | 0 |
| ENSRNOP00000039099 | Rpl35a | 3 | 0 |
| ENSRNOP00000053986 | RGD1565183 | 3 | 0 |
| ENSRNOP00000037396 | LOC100359986 | 3 | 0 |
| ENSRNOP00000046553 | LOC100360117 | 3 | 0 |
| ENSRNOP00000046515 | | 3 | 0 |
| ENSRNOP00000064197 | RGD1564883 | 3 | 0 |
| ENSRNOP00000047749 | LOC680441 | 3 | 0 |
| ENSRNOP00000031078 | Rpl31 | 3 | 0 |
| ENSRNOP00000049652 | LOC689899 | 3 | 0 |
| ENSRNOP00000065065 | Rpl26-ps3 | 3 | 0 |
| ENSRNOP00000021725 | Rpl12 | 3 | 0 |
| ENSRNOP00000041329 | LOC103693375 | 3 | 0 |
| ENSRNOP00000064566 | LOC100910370 | 3 | 0 |
| ENSRNOP00000067887 | LOC100910721 | 3 | 0 |
| ENSRNOP00000025421 | Rpl18a | 3 | 0 |
| ENSRNOP00000057262 | LOC690384 | 3 | 0 |
| ENSRNOP00000046301 | RGD1564839 | 3 | 0 |
| ENSRNOP00000054048 | Rpl36al | 3 | 0 |
| ENSRNOP00000048311 | RGD1563157 | 3 | 0 |
| ENSRNOP00000042127 | RGD1566373 | 3 | 0 |
| ENSRNOP00000051134 | | 3 | 0 |
| ENSRNOP00000041462 | LOC102555453 | 3 | 0 |
| ENSRNOP00000053863 | Rplp2 | 3 | 0 |
| ENSRNOP00000049014 | LOC100912182 | 3 | 0 |
| ENSRNOP00000055726 | Rpl28 | 3 | 0 |
| ENSRNOP00000057758 | Rpl26-ps2 | 3 | 0 |
| ENSRNOP00000044116 | RGD1561310 | 3 | 0 |
| ENSRNOP00000048003 | LOC100912182 | 3 | 0 |
| ENSRNOP00000054703 | Rpl34-ps1 | 3 | 0 |
| ENSRNOP00000044111 | RGD1565170 | 3 | 0 |
| ENSRNOP00000040232 | | 3 | 0 |
| ENSRNOP00000049054 | Rpl35al1 | 3 | 0 |
| ENSRNOP00000045849 | RGD1561195 | 3 | 0 |
| ENSRNOP00000042068 | | 3 | 0 |
| ENSRNOP00000040295 | Rpl39l | 3 | 0 |
| ENSRNOP00000045458 | Rpl26-ps1 | 3 | 0 |
| ENSRNOP00000040611 | Rpl35al1 | 3 | 0 |
| ENSRNOP00000047759 | | 3 | 0 |
| ENSRNOP00000030437 | RGD1563956 | 3 | 0 |
| ENSRNOP00000047328 | RGD1561333 | 3 | 0 |
| ENSRNOP00000050328 | RGD1562055 | 3 | 0 |
| ENSRNOP00000047010 | | 3 | 0 |
| ENSRNOP00000042920 | Rpl22l2 | 3 | 0 |
| ENSRNOP00000049205 | LOC100360841 | 3 | 0 |
| ENSRNOP00000046953 | LOC102554602 | 3 | 0 |
| ENSRNOP00000049635 | LOC102550734 | 3 | 0 |
| ENSRNOP00000027976 | Rpl13a | 3 | 0 |
| ENSRNOP00000056331 | Rpl37-ps1 | 3 | 0 |
| ENSRNOP00000042929 | LOC688981 | 3 | 0 |
| ENSRNOP00000046487 | RGD1562755 | 3 | 0 |
| ENSRNOP00000046281 | LOC686074 | 3 | 0 |
| ENSRNOP00000060662 | Rpl3 | 3 | 0 |
| ENSRNOP00000051427 | RGD1563958 | 3 | 0 |
| ENSRNOP00000051482 | Rpl31l3 | 3 | 0 |
| ENSRNOP00000066420 | LOC100361143 | 3 | 0 |
| ENSRNOP00000067596 | Rpl30l1 | 3 | 0 |
| ENSRNOP00000049666 | | 3 | 0 |
| ENSRNOP00000040073 | LOC103691563 | 3 | 0 |
| ENSRNOP00000041625 | LOC100362751 | 3 | 0 |
| ENSRNOP00000051114 | | 3 | 0 |
| ENSRNOP00000054474 | LOC100360654 | 3 | 0 |
| ENSRNOP00000025649 | Rab14 | 2 | 18381 |
| ENSRNOP00000025303 | Akt2 | 2 | 11234 |
| ENSRNOP00000038369 | Akt1 | 2 | 11234 |
| ENSRNOP00000018455 | Sec61a1 | 2 | 7772.48 |
| ENSRNOP00000036212 | Sec61a2 | 2 | 7772.48 |
| ENSRNOP00000064933 | Srsf1 | 2 | 2925 |
| ENSRNOP00000063941 | Ranbp2 | 2 | 2344 |
| ENSRNOP00000004797 | Tuba4a | 2 | 1792.33 |
| ENSRNOP00000013863 | Tubb4b | 2 | 1792.33 |
| ENSRNOP00000026792 | Actr1a | 2 | 1792.33 |
| ENSRNOP00000027370 | Tubg1 | 2 | 1792.33 |
| ENSRNOP00000065633 | Tubb4a | 2 | 1792.33 |
| ENSRNOP00000066312 | Dync1h1 | 2 | 1792.33 |
| ENSRNOP00000001415 | Elavl1 | 2 | 589 |
| ENSRNOP00000030371 | Rps18 | 2 | 4.44 |
| ENSRNOP00000039429 | | 2 | 4.44 |
| ENSRNOP00000040056 | RGD1561919 | 2 | 4.44 |
| ENSRNOP00000040282 | RGD1565912 | 2 | 4.44 |
| ENSRNOP00000042800 | | 2 | 4.44 |
| ENSRNOP00000042935 | Rps21-ps1 | 2 | 4.44 |
| ENSRNOP00000045940 | | 2 | 4.44 |
| ENSRNOP00000045954 | | 2 | 4.44 |
| ENSRNOP00000046036 | | 2 | 4.44 |
| ENSRNOP00000046090 | LOC100362298 | 2 | 4.44 |
| ENSRNOP00000046427 | | 2 | 4.44 |
| ENSRNOP00000047355 | | 2 | 4.44 |
| ENSRNOP00000047371 | Rps18l1 | 2 | 4.44 |
| ENSRNOP00000047767 | | 2 | 4.44 |
| ENSRNOP00000047911 | RGD1563352 | 2 | 4.44 |
| ENSRNOP00000048713 | | 2 | 4.44 |
| ENSRNOP00000049619 | | 2 | 4.44 |
| ENSRNOP00000049626 | | 2 | 4.44 |
| ENSRNOP00000056140 | | 2 | 4.44 |
| ENSRNOP00000065327 | | 2 | 4.44 |
| ENSRNOP00000063940 | | 2 | 4.44 |
| ENSRNOP00000067239 | | 2 | 4.44 |
| ENSRNOP00000064678 | LOC100912024 | 2 | 4.44 |
| ENSRNOP00000061250 | Rps11 | 2 | 4.44 |
| ENSRNOP00000063128 | Rpl7a | 2 | 4.44 |
| ENSRNOP00000000528 | Psmb8 | 2 | 0 |
| ENSRNOP00000000532 | Psmb9 | 2 | 0 |
| ENSRNOP00000000628 | Cdkn1a | 2 | 0 |
| ENSRNOP00000002037 | Psmb1 | 2 | 0 |
| ENSRNOP00000002358 | Psmd2 | 2 | 0 |
| ENSRNOP00000002834 | Mrpl1 | 2 | 0 |
| ENSRNOP00000004114 | LOC690271 | 2 | 0 |
| ENSRNOP00000005089 | Mrps7 | 2 | 0 |
| ENSRNOP00000005815 | Mrpl13 | 2 | 0 |
| ENSRNOP00000009249 | Psmd6 | 2 | 0 |
| ENSRNOP00000009649 | Psmc6 | 2 | 0 |
| ENSRNOP00000009666 | Psma6 | 2 | 0 |
| ENSRNOP00000010753 | Psma3 | 2 | 0 |
| ENSRNOP00000015278 | LOC680700 | 2 | 0 |
| ENSRNOP00000015618 | Psmb2 | 2 | 0 |
| ENSRNOP00000015747 | | 2 | 0 |
| ENSRNOP00000015757 | Psmc3 | 2 | 0 |
| ENSRNOP00000015946 | Psma1 | 2 | 0 |
| ENSRNOP00000016450 | Psmc2 | 2 | 0 |
| ENSRNOP00000016876 | Psmb7 | 2 | 0 |
| ENSRNOP00000017280 | Mrpl3 | 2 | 0 |
| ENSRNOP00000018005 | Psmb5 | 2 | 0 |
| ENSRNOP00000018173 | Psma4 | 2 | 0 |
| ENSRNOP00000019104 | Psmd7 | 2 | 0 |
| ENSRNOP00000019781 | Smurf2 | 2 | 0 |
| ENSRNOP00000024306 | Psmd1 | 2 | 0 |
| ENSRNOP00000025887 | Psme1 | 2 | 0 |
| ENSRNOP00000046157 | RGD1564469 | 2 | 0 |
| ENSRNOP00000027029 | Mrps12 | 2 | 0 |
| ENSRNOP00000031049 | RGD1563300 | 2 | 0 |
| ENSRNOP00000041612 | | 2 | 0 |
| ENSRNOP00000021899 | Mrps9 | 2 | 0 |
| ENSRNOP00000024326 | Mrto4 | 2 | 0 |
| ENSRNOP00000051848 | Mrpl12 | 2 | 0 |
| ENSRNOP00000044874 | RGD1559877 | 2 | 0 |
| ENSRNOP00000064904 | | 2 | 0 |
| ENSRNOP00000030289 | | 2 | 0 |
| ENSRNOP00000044949 | RGD1560633 | 2 | 0 |
| ENSRNOP00000065157 | | 2 | 0 |
| ENSRNOP00000064782 | | 2 | 0 |
| ENSRNOP00000023456 | Imp3 | 2 | 0 |
| ENSRNOP00000020451 | Mrps5 | 2 | 0 |
| ENSRNOP00000041821 | Eef2 | 2 | 0 |
| ENSRNOP00000055298 | | 2 | 0 |
| ENSRNOP00000036682 | Mrpl1 | 2 | 0 |
| ENSRNOP00000039797 | LOC103690888 | 2 | 0 |
| ENSRNOP00000048422 | RGD1562402 | 2 | 0 |
| ENSRNOP00000045798 | LOC367195 | 2 | 0 |
| ENSRNOP00000044837 | | 2 | 0 |
| ENSRNOP00000043087 | Gfm2 | 2 | 0 |
| ENSRNOP00000049519 | Efl1 | 2 | 0 |
| ENSRNOP00000019660 | Rpl3l | 2 | 0 |
| ENSRNOP00000048289 | | 2 | 0 |
| ENSRNOP00000034042 | mrpl24 | 2 | 0 |
| ENSRNOP00000027091 | mrpl11 | 2 | 0 |
| ENSRNOP00000045344 | RGD1559972 | 2 | 0 |
| ENSRNOP00000028517 | Mrpl16 | 2 | 0 |
| ENSRNOP00000048847 | | 2 | 0 |
| ENSRNOP00000041744 | RGD1564138 | 2 | 0 |
| ENSRNOP00000048624 | RGD1565415 | 2 | 0 |
| ENSRNOP00000021625 | Npsr1 | 2 | 0 |
| ENSRNOP00000042164 | LOC100359671 | 2 | 0 |
| ENSRNOP00000058859 | | 2 | 0 |
| ENSRNOP00000045007 | Mrpl17 | 2 | 0 |
| ENSRNOP00000048658 | LOC102554992 | 2 | 0 |
| ENSRNOP00000047999 | | 2 | 0 |
| ENSRNOP00000046067 | Mrps10 | 2 | 0 |
| ENSRNOP00000056689 | | 2 | 0 |
| ENSRNOP00000025007 | Mrps11 | 2 | 0 |
| ENSRNOP00000040081 | Gfm1 | 2 | 0 |
| ENSRNOP00000061911 | | 2 | 0 |
| ENSRNOP00000063004 | | 2 | 0 |
| ENSRNOP00000024380 | Mrpl2 | 2 | 0 |
| ENSRNOP00000021803 | Rpl7l1 | 2 | 0 |
| ENSRNOP00000029336 | Mrpl22 | 2 | 0 |
| ENSRNOP00000067306 | | 2 | 0 |
| ENSRNOP00000051317 | | 2 | 0 |
| ENSRNOP00000031927 | Smurf1 | 2 | 0 |
| ENSRNOP00000050173 | Cdkn1b | 2 | 0 |
| ENSRNOP00000026928 | Psma5 | 2 | 0 |
| ENSRNOP00000042447 | Psma8 | 2 | 0 |
| ENSRNOP00000028484 | Psmb4 | 2 | 0 |
| ENSRNOP00000026507 | Psmb6 | 2 | 0 |
| ENSRNOP00000028589 | Psmd4 | 2 | 0 |
| ENSRNOP00000062146 | Psmd11 | 2 | 0 |
| ENSRNOP00000000529 | Tap1 | 1 | 0 |
| ENSRNOP00000000559 | Daxx | 1 | 0 |
| ENSRNOP00000000612 | Srpk1 | 1 | 0 |
| ENSRNOP00000000627 | Srsf3 | 1 | 0 |
| ENSRNOP00000000750 | Ube2d1 | 1 | 0 |
| ENSRNOP00000000783 | Cdk1 | 1 | 0 |
| ENSRNOP00000001115 | Ddx39b | 1 | 0 |
| ENSRNOP00000001154 | Rbmx | 1 | 0 |
| ENSRNOP00000001402 | Snrnp35 | 1 | 0 |
| ENSRNOP00000001539 | Srsf9 | 1 | 0 |
| ENSRNOP00000001685 | Clip1 | 1 | 0 |
| ENSRNOP00000001817 | Mapkapk5 | 1 | 0 |
| ENSRNOP00000001954 | Ywhag | 1 | 0 |
| ENSRNOP00000003060 | Hspbap1 | 1 | 0 |
| ENSRNOP00000003696 | Pafah1b1 | 1 | 0 |
| ENSRNOP00000004283 | Psmd12 | 1 | 0 |
| ENSRNOP00000004947 | Mrpl27 | 1 | 0 |
| ENSRNOP00000005016 | Prpf8 | 1 | 0 |
| ENSRNOP00000005267 | Ocrl | 1 | 0 |
| ENSRNOP00000005329 | Psmc1 | 1 | 0 |
| ENSRNOP00000005577 | Rps29 | 1 | 0 |
| ENSRNOP00000005832 | Klhl12 | 1 | 0 |
| ENSRNOP00000006148 | Traf6 | 1 | 0 |
| ENSRNOP00000006953 | Dync2li1 | 1 | 0 |
| ENSRNOP00000007100 | Ywhae | 1 | 0 |
| ENSRNOP00000007258 | Rab3ip | 1 | 0 |
| ENSRNOP00000007583 | Srsf5 | 1 | 0 |
| ENSRNOP00000007620 | Cul1 | 1 | 0 |
| ENSRNOP00000007676 | Skp1 | 1 | 0 |
| ENSRNOP00000007963 | Cdc27 | 1 | 0 |
| ENSRNOP00000008120 | Dctn2 | 1 | 0 |
| ENSRNOP00000008355 | Sf3a1 | 1 | 0 |
| ENSRNOP00000008427 | Srsf6 | 1 | 0 |
| ENSRNOP00000008492 | Aurkb | 1 | 0 |
| ENSRNOP00000011619 | Mrpl15 | 1 | 0 |
| ENSRNOP00000011653 | Ube2e1 | 1 | 0 |
| ENSRNOP00000012279 | Dynll2 | 1 | 0 |
| ENSRNOP00000012984 | Mdm4 | 1 | 0 |
| ENSRNOP00000013184 | Dync1i1 | 1 | 0 |
| ENSRNOP00000013204 | Casc3 | 1 | 0 |
| ENSRNOP00000013301 | Srsf4 | 1 | 0 |
| ENSRNOP00000015152 | Hnrnpa2b1 | 1 | 0 |
| ENSRNOP00000015518 | Hnrnph2 | 1 | 0 |
| ENSRNOP00000015813 | Tnks | 1 | 0 |
| ENSRNOP00000016220 | Mapre1 | 1 | 0 |
| ENSRNOP00000016946 | Plk4 | 1 | 0 |
| ENSRNOP00000017353 | Ehd2 | 1 | 0 |
| ENSRNOP00000017718 | LOC100912445 | 1 | 0 |
| ENSRNOP00000018278 | Kif26a | 1 | 0 |
| ENSRNOP00000019561 | Dctn3 | 1 | 0 |
| ENSRNOP00000019642 | Psmd13 | 1 | 0 |
| ENSRNOP00000020065 | Mapkapk3 | 1 | 0 |
| ENSRNOP00000020323 | Ube2c | 1 | 0 |
| ENSRNOP00000021528 | Cul3 | 1 | 0 |
| ENSRNOP00000022779 | Actr1b | 1 | 0 |
| ENSRNOP00000023342 | Ide | 1 | 0 |
| ENSRNOP00000025433 | Psmd5 | 1 | 0 |
| ENSRNOP00000028176 | Snrnp70 | 1 | 0 |
| ENSRNOP00000029646 | Dync1li2 | 1 | 0 |
| ENSRNOP00000029790 | Eif3el1 | 1 | 0 |
| ENSRNOP00000032361 | Eif3d | 1 | 0 |
| ENSRNOP00000038586 | Klhdc10 | 1 | 0 |
| ENSRNOP00000040703 | Rps29 | 1 | 0 |
| ENSRNOP00000043252 | Dync2h1 | 1 | 0 |
| ENSRNOP00000043254 | Eif4h | 1 | 0 |
| ENSRNOP00000043270 | Rps29 | 1 | 0 |
| ENSRNOP00000044909 | Rps29 | 1 | 0 |
| ENSRNOP00000054753 | Disc1 | 1 | 0 |
| ENSRNOP00000057188 | Eif3b | 1 | 0 |
| ENSRNOP00000058234 | Clasp1 | 1 | 0 |
| ENSRNOP00000053643 | Chek2 | 1 | 0 |
| ENSRNOP00000050029 | Rangap1 | 1 | 0 |
| ENSRNOP00000024406 | Ube2i | 1 | 0 |
| ENSRNOP00000020402 | Sae1 | 1 | 0 |
| ENSRNOP00000013548 | Mrps2 | 1 | 0 |
| ENSRNOP00000066181 | Naca | 1 | 0 |
| ENSRNOP00000035155 | Srsf7 | 1 | 0 |
| ENSRNOP00000039298 | Snrpb | 1 | 0 |
| ENSRNOP00000019529 | Hnrnpf | 1 | 0 |
| ENSRNOP00000057257 | LOC100911576 | 1 | 0 |
| ENSRNOP00000052955 | Srrm1 | 1 | 0 |
| ENSRNOP00000026033 | Nxf1 | 1 | 0 |
| ENSRNOP00000051838 | Alyref | 1 | 0 |
| ENSRNOP00000042416 | Snrpep2 | 1 | 0 |
| ENSRNOP00000065463 | Lsm2 | 1 | 0 |
| ENSRNOP00000048598 | Snrnp200 | 1 | 0 |
| ENSRNOP00000061368 | Dhx9 | 1 | 0 |
| ENSRNOP00000060194 | Upf3a | 1 | 0 |
| ENSRNOP00000052160 | Hnrnpa1 | 1 | 0 |
| ENSRNOP00000021221 | Snrpd2 | 1 | 0 |
| ENSRNOP00000066302 | Eif4a3 | 1 | 0 |
| ENSRNOP00000019126 | Sf3b1 | 1 | 0 |
| ENSRNOP00000055962 | Hnrnph1 | 1 | 0 |
| ENSRNOP00000018646 | Snrpd1 | 1 | 0 |
| ENSRNOP00000023854 | Sf3b3 | 1 | 0 |
| ENSRNOP00000046491 | Hnrnpd | 1 | 0 |
| ENSRNOP00000028807 | Rbm8a | 1 | 0 |
| ENSRNOP00000025980 | Hnrnpk | 1 | 0 |
| ENSRNOP00000027425 | Hnrnpl | 1 | 0 |
| ENSRNOP00000058923 | Cdc40 | 1 | 0 |
| ENSRNOP00000032108 | Hnrnpm | 1 | 0 |
| ENSRNOP00000019810 | Dhx38 | 1 | 0 |
| ENSRNOP00000029696 | Upf3b | 1 | 0 |
| ENSRNOP00000038996 | LOC688526 | 1 | 0 |
| ENSRNOP00000061492 | Polr2a | 1 | 0 |
| ENSRNOP00000024529 | Rbm5 | 1 | 0 |
| ENSRNOP00000045893 | Mrpl4 | 1 | 0 |
| ENSRNOP00000021837 | Ckap5 | 1 | 0 |
| ENSRNOP00000045795 | Optn | 1 | 0 |
| ENSRNOP00000033018 | Tbc1d1 | 1 | 0 |
| ENSRNOP00000065234 | Rab10 | 1 | 0 |
| ENSRNOP00000040878 | Gapdh | 1 | 0 |
| ENSRNOP00000026224 | Stx4 | 1 | 0 |
| ENSRNOP00000039818 | RGD1562758 | 1 | 0 |
| ENSRNOP00000054528 | Psmd9 | 1 | 0 |
| ENSRNOP00000032191 | Cdk2 | 1 | 0 |
| ENSRNOP00000065348 | Psmb11 | 1 | 0 |
| ENSRNOP00000037928 | Psmd3 | 1 | 0 |
| ENSRNOP00000066229 | | 1 | 0 |
| ENSRNOP00000051977 | Aurka | 1 | 0 |
| ENSRNOP00000066950 | Psma2 | 1 | 0 |
| ENSRNOP00000055036 | RGD1564425 | 1 | 0 |
| ENSRNOP00000032953 | RGD1562029 | 1 | 0 |
| ENSRNOP00000051863 | Tgfb1i1 | 1 | 0 |
| ENSRNOP00000057786 | Mapkapk2 | 1 | 0 |
| ENSRNOP00000063449 | Mdm2 | 1 | 0 |
| ENSRNOP00000063831 | Dnmt1 | 1 | 0 |
| ENSRNOP00000067005 | U2af1 | 1 | 0 |
| ENSRNOP00000060919 | Dync1i2 | 1 | 0 |
| ENSRNOP00000059841 | Dnah1 | 1 | 0 |
| ENSRNOP00000061342 | Dynll1 | 1 | 0 |
| ENSRNOP00000061642 | Rps10 | 1 | 0 |

| **Table 11.** GO enrichment of WAT protein-protein interaction network | | | | | |
| --- | --- | --- | --- | --- | --- |
| **Pathway** | **Total** | **Expected** | **Hits** | **P.Value** | **FDR** |
| **Biological Process** | |  |  |  |  |
| Chromatin assembly or disassembly | 248 | 2.85 | 74 | 5.83E-91 | 3.93E-88 |
| Sensory perception of taste | 5 | 0.0575 | 5 | 1.87E-10 | 6.31E-08 |
| Ras protein signal transduction | 63 | 0.725 | 10 | 2.21E-09 | 4.97E-07 |
| Positive regulation of DNA metabolic process | 90 | 1.04 | 11 | 5.94E-09 | 1.00E-06 |
| Regulation of organelle organization | 51 | 0.587 | 6 | 2.44E-05 | 0.00329 |
| Chromatin remodeling | 38 | 0.437 | 5 | 6.91E-05 | 0.00778 |
| **Molecular Function** | |  |  |  |  |
| RNA binding | 270 | 3.65 | 87 | 1.53E-104 | 5.06E-102 |
| Transcription cofactor activity | 356 | 4.81 | 33 | 7.17E-19 | 1.19E-16 |
| Nucleotide binding | 273 | 3.69 | 23 | 1.82E-12 | 2.01E-10 |
| Transcription corepressor activity | 101 | 1.36 | 14 | 6.98E-11 | 5.78E-09 |
| RNA helicase activity | 49 | 0.662 | 5 | 0.000499 | 0.033 |
| **Cellular Component** | | |  |  |  |
| Microtubule organizing center | 109 | 1.03 | 26 | 1.02E-29 | 1.80E-27 |
| Proteasome complex | 44 | 0.416 | 13 | 1.12E-16 | 9.81E-15 |
| Endomembrane system | 14 | 0.132 | 7 | 1.89E-11 | 1.11E-09 |
| Clathrin_coated vesicle | 85 | 0.803 | 10 | 7.09E-09 | 3.12E-07 |
| Vesicle membrane | 1670 | 15.8 | 39 | 5.26E-08 | 1.85E-06 |
| Mitochondrial matrix | 18 | 0.17 | 5 | 5.46E-07 | 1.60E-05 |
| Nucleus | 3830 | 36.2 | 63 | 7.37E-07 | 1.85E-05 |
| Golgi stack | 376 | 3.55 | 15 | 2.74E-06 | 6.04E-05 |
| Transcription factor TFIID complex | 62 | 0.586 | 6 | 2.55E-05 | 0.000499 |
| U12_type spliceosomal complex | 612 | 5.78 | 17 | 6.63E-05 | 0.00117 |
| Apical plasma membrane | 122 | 1.15 | 7 | 0.000158 | 0.00252 |
| Nucleoplasm | 1450 | 13.7 | 26 | 0.00107 | 0.0157 |
| Synapse | 23 | 0.217 | 3 | 0.00127 | 0.0173 |
| Adherens junction | 91 | 0.86 | 5 | 0.00171 | 0.0214 |
| Nucleolus | 1280 | 12.1 | 23 | 0.00192 | 0.0226 |
| Kinesin complex | 29 | 0.274 | 3 | 0.00252 | 0.0278 |
